# Identifying high-performance catalytic conditions for carbon dioxide reduction to dimethoxymethane by multivariate modelling†
†Electronic supplementary information (ESI) available: Synthetic procedures, multivariate modelling procedure, additional tables, catalysis data, R-code. See DOI: 10.1039/c9sc04591k


**DOI:** 10.1039/c9sc04591k

**Published:** 2019-10-24

**Authors:** Max Siebert, Gerhard Krennrich, Max Seibicke, Alexander F. Siegle, Oliver Trapp

**Affiliations:** a Department Chemie , Ludwig-Maximilians-Universität München , Butenandtstr. 5-13 , 81377 München , Germany . Email: oliver.trapp@cup.uni-muenchen.de

## Abstract

In times of a warming climate due to excessive carbon dioxide production, catalytic conversion of carbon dioxide to formaldehyde is not only a process of great industrial interest, but it could also serve as a means for meeting our climate goals. Currently, formaldehyde is produced in an energetically unfavourable and atom-inefficient process. A much needed solution remains academically challenging. Here we present an algorithmic workflow to improve the ruthenium-catalysed transformation of carbon dioxide to the formaldehyde derivative dimethoxymethane. Catalytic processes are typically optimised by comprehensive screening of catalysts, substrates, reaction parameters and additives to enhance activity and selectivity. The common problem of the multidimensionality of the parameter space, leading to only incremental improvement in laborious physical investigations, was overcome by combining elements from machine learning, optimisation and experimental design, tripling the turnover number of 786 to 2761. The optimised conditions were then used in a new reaction setup tailored to the process parameters leading to a turnover number of 3874, exceeding by far those of known processes.

## Abstract 2

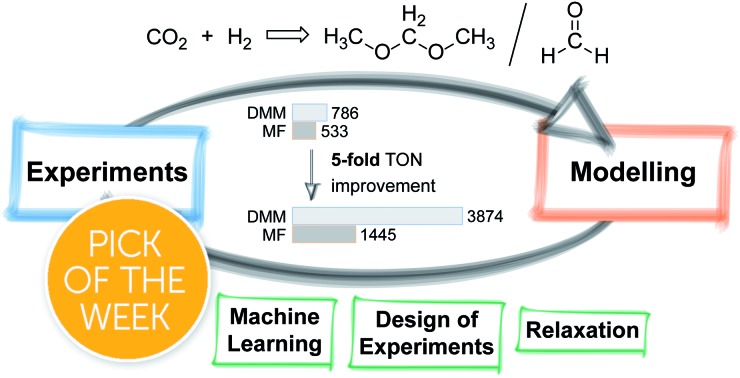
An efficient algorithmic workflow was developed to optimize seven process parameters of a homogeneous catalytic system with minimal experimental effort.

## Introduction

In this century, our ecosystem faces severe problems such as global warming, environmental pollution and resource depletion. The negative impact of humankind as well as its responsibility in solving these problems can no longer be ignored.^1^,^2^ Besides the necessity for future-oriented global politics,^3^,^4^ both the scientific community and the chemical industry must provide answers to crucial questions regarding sustainable process development, alternative energies as well as recycling of waste and pollutants.^5^–^9^ In this context, more efficient techniques must be developed and applied in research to reduce time, cost and resources.^10^–^12^

In catalytic investigations, system optimisation is typically approached by one-factor-at-a-time (OFAT) methods, successively screening along one parameter axis. Once optimised, a parameter is kept constant for the subsequent experiments. In this univariate analysis, variables are treated as being independent of each other. Beside the vast number of experiments that must be performed, local maxima with higher performances might be missed. Consequently, algorithm-based screening and optimisation techniques have been among the fastest growing research areas in recent years.^13^–^24^ Considering the interactions of parameters, optimised results can be achieved with minimal experimental effort.^25^,^26^

**Scheme 1:**
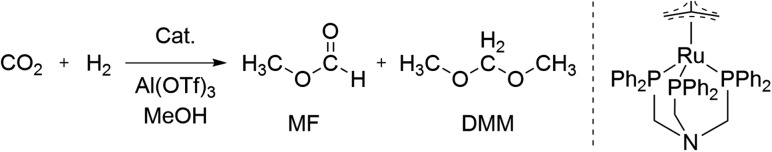
Reductive transformation of CO_2_ towards the formaldehyde oxidation level yielding methyl formate (MF) and dimethoxymethane (DMM).

Recently, we applied a univariate optimisation approach including several hundred catalytic reactions to improve the selective ruthenium-catalysed transformation of carbon dioxide to dimethoxymethane (DMM) reaching a turnover number (TON) of 786 (Scheme 1).^27^,^28^ The product DMM itself is a high value feedstock for biofuels, but can also be hydrolysed yielding formaldehyde and methanol or directly employed as a formaldehyde synthon.^29^,^30^ Beside the desired product, only methyl formate (MF) was formed with TONs of up to 1290 (Scheme 1).^27^ Previously, two studies on the selective hydrogenation of CO_2_ by the group of Klankermayer showed the formation of DMM and MF by using a homogeneous ruthenium catalyst^31^ with TONs of 214 and 104 or a cobalt catalyst^32^ with TONs of 157 and 37, respectively. Further selective reductions toward the formaldehyde oxidation state were reported utilising hydroboration,^33^–^37^ hydrosilylation,^38^,^39^ and frustrated Lewis pairs,^40^,^41^ however, being mainly of academic interest due to the stoichiometric use of reducing reagents.

**Fig. 1:**
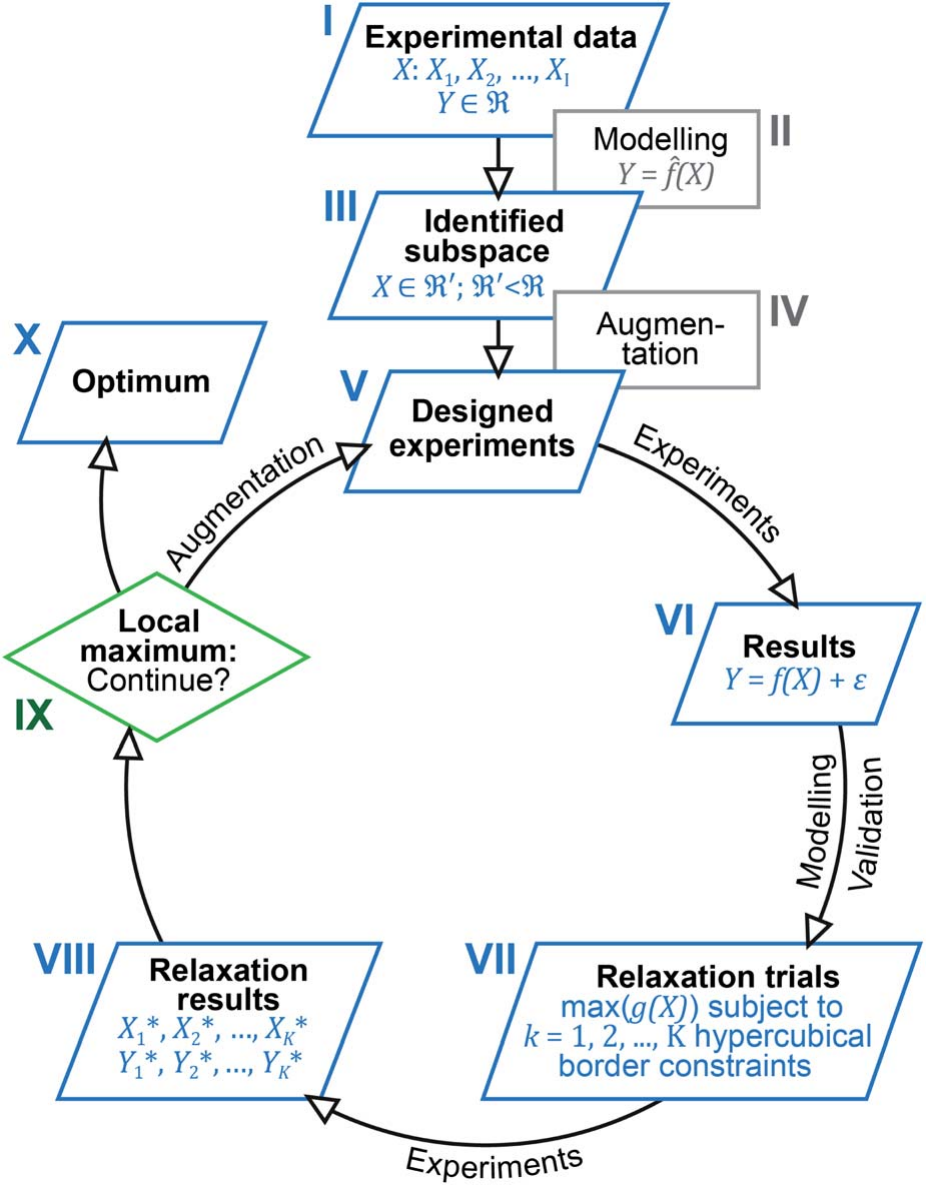
Outline of the algorithm-based workflow combining experimental data analysis, DoE and optimisation.

Inspired by previous reports, which underline the importance of a holistic view on the results obtained,^13^,^19^ we now utilised multivariate analysis^42^,^43^ to further optimise the investigated catalysis. Based on the large amount of experimental data and the dependency of the catalytic system on seven different parameters, we envisioned to increase its performance by modelling and predicting conditions optimally accounting for parameter interaction. For this purpose, we devised a multi-step process combining elements from machine learning, optimisation and experimental design (design of experiments, DoE). Fig. 1 gives a schematic overview of the easy-to-use algorithmic workflow, which was developed in this work and is applicable for any catalytic screening. Experimental data (Fig. 1(I)) was modelled using the random forest (RF) algorithm (Fig. 1(II)) to identify promising subspaces with high catalytic performance (Fig. 1(III)). To better understand the origin of the exceptional activity and to extend the amount of data for further modelling, the subspace was augmented (Fig. 1(IV)) by additional experiments (Fig. 1(V)) that were based on experimental design. Starting from these first experiments, an iterative workflow – consisting of DoE (Fig. 1(V) and (VI)), optimisation (relaxation; Fig. 1(VII) and (VIII)) and evaluation (Fig. 1(IX)) – was applied until the final optimum was reached (Fig. 1(X)).

## Results and discussion

### Catalytic system

The selective transformation of CO_2_ to DMM was performed using the ruthenium catalyst [Ru(*N*-triphos^Ph^)(tmm)] (tmm = trimethylenemethane dianion) for hydride transfer and Al(OTf)_3_ to facilitate the acetalisation with methanol (Scheme 1). Beneficially, the robust catalyst can be obtained on a large scale in a simple three-step procedure, which underlines its suitability for industrial and large scale applications.^27^ The catalysis is mainly affected by the reaction temperature (*T*), partial pressure of H_2_ (*p*_H_2__) and CO_2_ (*p*_CO_2__), reaction time (*t*), the amount of catalyst (*n*_cat_) and Lewis acid (*n*_add_) as well as the reaction volume (*V*).

### Experimental data analysis

Following the rationale outlined in the previous section, the optimisation started with a corpus of 49 unique settings of the seven process factors (*X*: *X*_1_, *X*_2_, …, *X*_7_ = *T*, *p*_H_2__, *p*_CO_2__, *t*, *n*_cat_, *n*_add_, *V*), two responses (*Y*: *Y*_1_, *Y*_2_ = TON_DMM_, TON_MF_) and 144 observations, which are measured values of the responses (Table 1, Fig. 1(I), ESI Table 1†).^27^ Each setting was run as a triple replicate, except for three settings that were realised as simple replicates, thus making up the 144 cases of the dataset. Goal of the optimisation was finding reaction conditions 
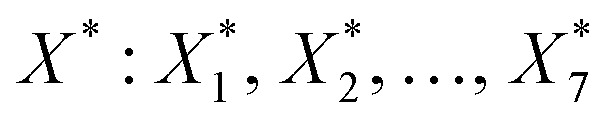
, that maximise TON_DMM_ (for a theoretical introduction on this approach, see the Methods section). As process factors, six of the previously investigated parameters, namely temperature, partial pressure of H_2_ and CO_2_, reaction time as well as the amount of catalyst and Lewis acid, proved suitable for the optimisation. The influence of varying the nature of the Lewis acid was neglected here, because statistical analysis of the dataset revealed Al(OTf)_3_ as the only promising candidate. Instead, the volume of the catalysis solution was considered for the multivariate analysis to account for mass transfer phenomena and gas solubility effects (concentration effects) potentially influencing the performance of the catalyst.

**Table 1:** Factors and responses of the dataset^27^

Entry	Variable	Mean	Median	Min	Max
1	*T* (°C)	89.2	90	20	120
2	*p*_H_2__ (bar)	82.1	90	40	100
3	*p*_CO_2__ (bar)	17.9	20	5	40
4	*t* (h)	26.5	18	1	168
5	*n*_cat_ (μmol)	1.3	1.5	0	3
6	*n*_add_ (μmol)	5.4	6.25	0	12.5
7	*V* (mL)	0.5	0.5	0.25	0.5
8	TON_DMM_	275	263	0	906
9	TON_MF_	145	71	0	1377

Modelling and analysis of datasets based on empirical optimisation (*e.g.* OFAT) is complex and more difficult than estimating parametric surrogate models from well-designed data. The difficulties arise primarily from non-linearities, which simple parametric models cannot appropriately describe. Furthermore, OFAT variations often and inadvertently lead to co-varying and thereby confounding the effects of process parameters. For instance, in the present dataset the process parameters *n*_cat_ and *n*_add_ were found to be highly correlated with a Pearson correlation coefficient *r*(*n*_cat_ – *n*_add_) = 0.895, rendering the data non-informative in terms of the independent effects of *n*_cat_ and *n*_add_.

Next, the problem for these datasets is finding an appropriate functional representation, *f[combining circumflex]*(), of the target response as a function of the process factors *X*_*i*_, that is TON_DMM_ = *f*(*T*,*p*_H_2__,*p*_CO_2__,*t*,*n*_cat_,*n*_add_,*V*) + *ε*, without making any prior assumptions about the analytical form of the ‘true’ function *f*(). Here, we used RF as a powerful non-linear and easy-to-use method for the empirical model building of complex datasets (Fig. 1(II)).^44^,^45^

**Fig. 2:**
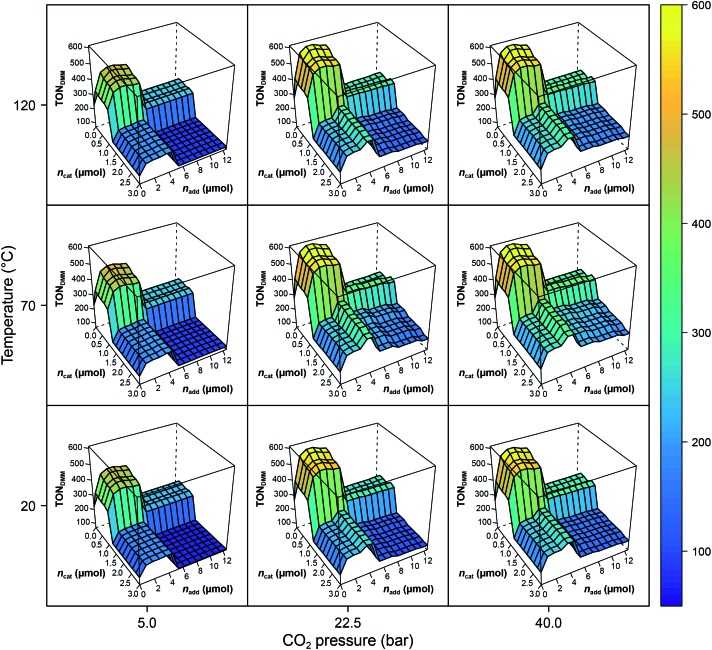
Trellis plot of the random forest predictions *f[combining circumflex]*(*T*,*p*_H_2__,*p*_CO_2__,*t*,*n*_cat_,*n*_add_,*V*) with *p*_H_2__ = 90 bar, *t* = 18 h and *V* = 0.5 mL. Note the difference of max(TON_DMM_) in the experimental data (Table 1, entry 8) and the model. The RF values are predictions causing the range of the *z*-axis to be smaller than the range of the empirical data.

The RF models describe 79% of the responses' variance on average (*R*^2^(TON_DMM_) = 0.83; *R*^2^(TON_MF_) = 0.74), which is a good result given the heterogeneous nature of the dataset. Further, tuning the RF hyperparameters using 10-fold cross validation with consecutive blocks (mtry* = 2 with *R*^2^ = 0.83) revealed that the default RF hyperparameters describe the data appropriately. The effect structure of the models can be conveniently explored by plotting the RF model predictions *f[combining circumflex]*(*X*) against the process factors *X*_1_, *X*_2_, …, *X*_7_. Fig. 2 shows the effects of the process parameters *T*, *p*_CO_2__, *n*_cat_ and *n*_add_, given the median values *p*_H_2__ = 90 bar, *t* = 18 h and *V* = 0.5 mL, as a conditional trellis plot.

As a tree-based ensemble method, RF splits and mean-aggregates the experimental space into hyperrectangles, the consequence being that the RF model surface becomes non-smooth (Fig. 2). This property of RF is a particular strength when it comes to identifying promising domains of the experimental space and was another motivation for choosing RF as modelling technique.

Evidently, *n*_cat_ and *n*_add_ exert strong non-linear, step like negative effects on the process performance (TON_DMM_), dividing the *n*_cat_ × *n*_add_ space into domains of different performance. The pressure, *p*_CO_2__, reveals a positive and temperature, *T*, a small convex effect, both, however, negligible compared to the dominant effects of *n*_cat_ and *n*_add_. At this point, the erroneous impression may arise that the heuristic interpretation of the experimental data could lead to a further improvement by a trivial reduction of the amount of catalyst, which is often reduced to minimise catalyst deactivation. However, a closer look at the experimental data shows that a reduction in the amount of catalyst without changing other parameters leads to a decrease in catalytic activity with respect to DMM. This case shows exemplarily how important multivariate analytical methods can be, because they can easily identify complex correlations of process parameters that are not detectable by a univariate analysis of the scientist.

With the condition TON_DMM_ > 400 deduced from the landscape of TON_DMM_ over *n*_cat_ × *n*_add_ (Fig. 2), a subset of 26 observations with 9 unique settings was selected from the data (Fig. 1(III)). In this subset, the parameters *T*, *p*_H_2__, *p*_CO_2__ and *t* were found constant at *T* = 90 °C, *p*_H_2__ = 90 bar, *p*_CO_2__ = 20 bar and *t* = 18 h, thereby suggesting to conditionally optimise *n*_cat_, *n*_add_ and *V* first, while keeping *T*, *p*_H_2__, *p*_CO_2__ and *t* constant for the time being.

### First DoE

These 9 candidate points (identified unique settings mentioned above) were subsequently augmented by an additional number of 6 points to fully support all second order effects of *n*_cat_, *n*_add_ and *V* (Fig. 1(IV)). The intention behind this augmentation was to render the formerly confounded effects of *n*_cat_ and *n*_add_ estimable as well as, assuming high complexity, to allow for interactions and non-linearities of the process parameters as a solid basis for further optimisation (Fig. 1(V)).

The 6 augmentation trials were realised as triple replicates in the lab, added to the 9 settings already available and the responses (TON_DMM_, TON_MF_) were modelled as a function of the least square parameters with stepwise ordinary least squares (OLS; Fig. 1(VI)). Both models accurately describe the data within the replication error and explain 91% (*R*^2^(TON_DMM_) = 0.91; DF = 37) and 97% (*R*^2^(TON_MF_) = 0.97; DF = 35) of the responses' variance with DF denoting the degrees of freedom (number of data points minus number of estimated model parameters).

**Fig. 3:**
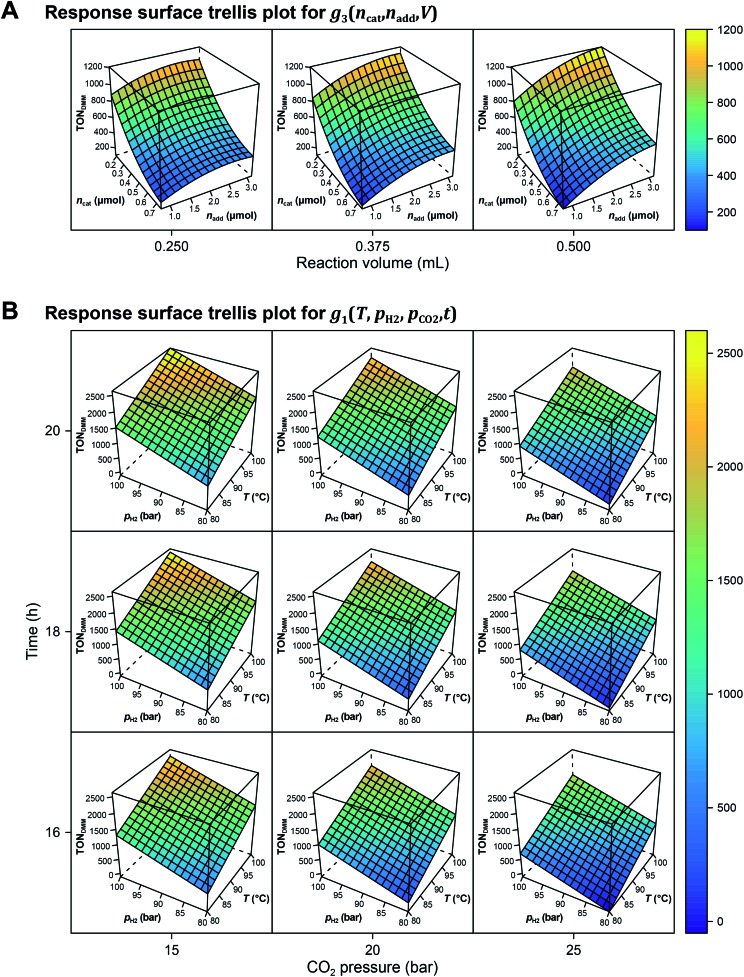
Calculated response surfaces. (A) Trellis plot of the response surface for *g*_3_(*n*_cat_,*n*_add_,*V*) from OLS modelling with *T* = 90 °C, *p*_H_2__ = 90 bar, *p*_CO_2__ = 20 bar and *t* = 18 h. (B) Response surface trellis plot of the linear OLS model for *g*_1_(*T*,*p*_H_2__,*p*_CO_2__,*t*) with 
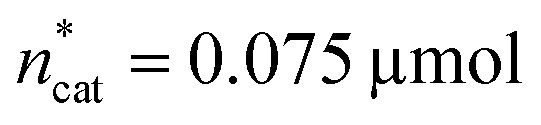
, 
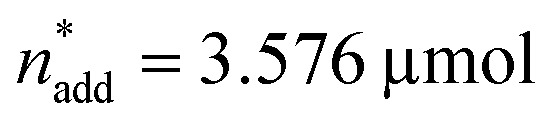
 and V* = 0.550 mL. For a mathematical definition of the polynomial parametric surrogates, *g*_1,3_(), see the Methods section.

**Fig. 4:**
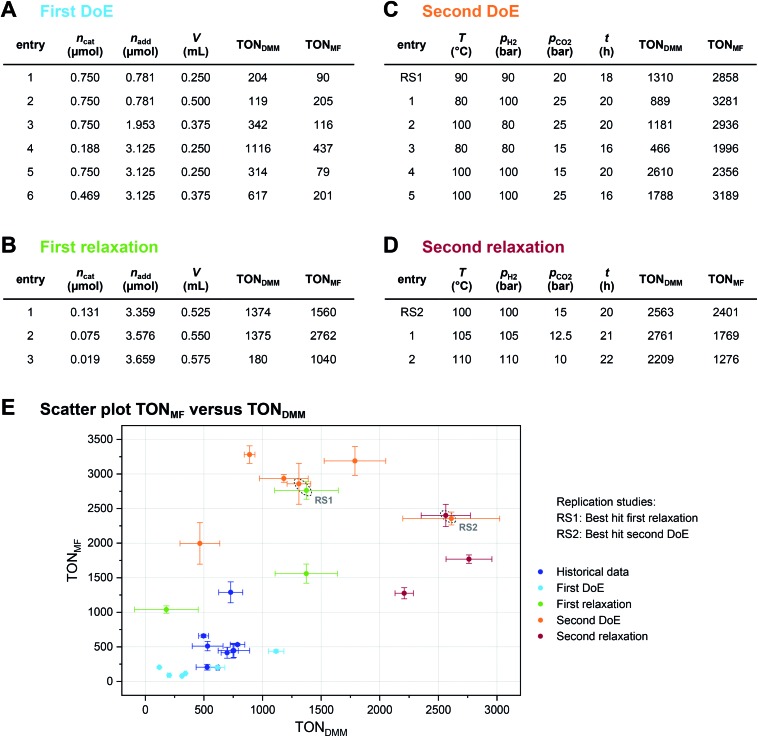
Optimisation summary. Process parameters and TONs for DMM and MF at different optimisation steps: (A) first DoE. (B) First relaxation (10, 20 and 30%). (C) Second DoE. (D) Second relaxation (25 and 50%). (E) Scatter plot TON_MF_*versus* TON_DMM_ summarising the outcome of the optimisation project. The results of each optimisation step are marked according to the colour code. Replication studies (RS): the circled data points label the best hit of the first relaxation and the respective replication trials in the second DoE (RS1) as well as the best hit of the second DoE and the respective replication trials in the second relaxation (RS2).

The local effects of *n*_cat_, *n*_add_ and *V* are depicted in Fig. 3A as response surface trellis plot. Again, there is a strong negative effect for *n*_cat_ along with positive effects for *n*_add_ and *V*. Together with the positive, synergistic effect between *n*_add_ and *V*, the effect structure suggests to decrease *n*_cat_ and to increase *n*_add_ and *V* to further maximise the catalytic performance beyond the best result of the first DoE (Fig. 4A, entry 4).

### First relaxation

To optimise towards the direction of maximal improvement, following the procedure outlined in the Methods section [eqn (5)], the experimental space was relaxed with 10% step size. The triple obtained from relaxing the design space together with the achieved experimental results are listed in Fig. 4B (Fig. 1(VII) and (VIII); see the Methods section).

The joint condition max(TON_DMM_), max(TON_MF_) was best met by the 20% relaxation trial (Fig. 4B, entry 2), and the factor setting 
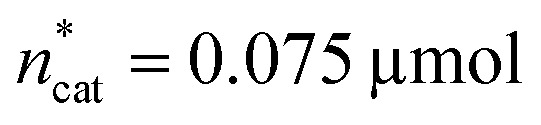
, 
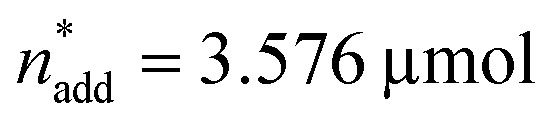
 and V* = 0.550 mL thus became the reference point for further optimisation. The 30% relaxation trial was very poor, indicating that a local maximum had been exceeded (Fig. 4B, entry 3). The pronounced drop in activity, resulting most likely from the reduced catalyst loading, indicates a molecular deactivation pathway of the catalytically active species due to potential inhibitors, such as carbon monoxide, moisture and oxygen, which are probably present in low concentrations.

At this point, there were two alternatives to proceed: (1) create an experimental design around 
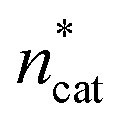
, 
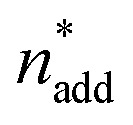
 and V* to fully identify the topology around the relaxation point at the desired resolution (complexity). (2) Consider 
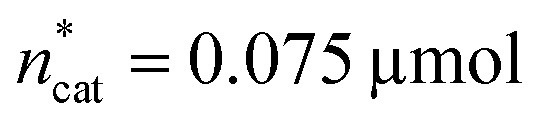
, 
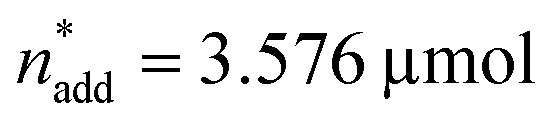
 and V* = 0.550 mL as locally optimal and switch to optimising the candidates *T*, *p*_H_2__, *p*_CO_2__ and *t*, which had so far been kept constant.

The poor outcome of the third relaxation trial (Fig. 4B, entry 3) showed that not much was to be expected from exploring the three-dimensional environment of the 20% relaxation trial any further. Therefore, option 2 was chosen and the 20% relaxation trial became the reference point for optimising the candidates *T*, *p*_H_2__, *p*_CO_2__ and *t*.

### Second DoE

A small linear design [eqn (2)] with 5 runs in the ranges listed in Fig. 4C was created with the reference point 
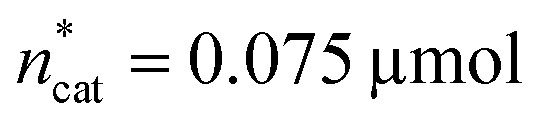
, 
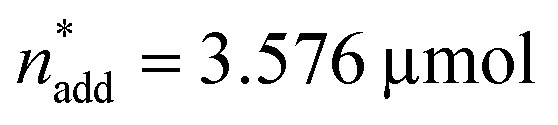
 and V* = 0.550 mL, T* = 90 °C, 
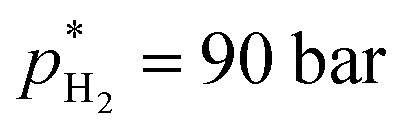
, 
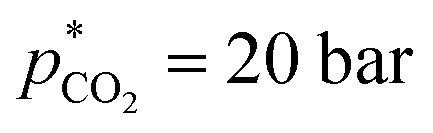
 bar, t* = 18 h at the design centre as replicate (Fig. 1(V); see the Methods section). The experiments were realised in the lab as triple replicate to provide a measure of accuracy (Fig. 1(VI)).

The measured responses (TON_DMM_, TON_MF_) were linearly modelled as a function of the process parameters with stepwise OLS. The models explain 92% (*R*^2^(TON_DMM_) = 0.92; DF = 16) and 78% (*R*^2^(TON_MF_) = 0.78; DF = 18) variance of the responses thus indicating a large signal-to-noise ratio for TON_DMM_ and to a lesser extent for TON_MF_.

Fig. 3B shows the linear effects of the process parameters on TON_DMM_ as trellis response surface plot. The factors *T* and *p*_H_2__ both have strong positive effects on TON_DMM_, whereas *t* reveals only a small positive and *p*_CO_2__ a moderate negative effect on TON_DMM_. Optimal conditions were found in the upper left panel and these are the conditions of the top candidate found in the design list with *T*, *p*_H_2__, *t* at the upper and *p*_CO_2__ at the lower bound, yielding respective TONs for DMM and MF of 2610 and 2356 (Fig. 4C, entry 4).

### Second relaxation

Following eqn (5), the experimental space was relaxed in 25% and 50% steps and the relaxation trials were experimentally realised in the lab (Fig. 4D and 1(VII); see the Methods section).

Again, we saw a small improvement of the 25% relaxation trial (Fig. 4D, entry 1) compared with the best candidate from the second DoE (Fig. 4C, entry 4), whereas the 50% relaxation candidate performed comparatively poorly (Fig. 4D, entry 2; Fig. 1(VIII)). With these relaxation trials, we reached the technical limits of our setup regarding hydrogen gas pressure, and therefore, the conditions of the 25% relaxation experiment can be considered locally optimal given the constraints of technical feasibility (Fig. 1(IX) and (X)). We would like to point out that the catalytic conditions optimised by the here presented strategy may still represent a local maximum. Modification of the catalyst and the additive might also result in further improvement, but as demonstrated in this work the identification of high-performance catalytic conditions should be a prerequisite for a design strategy of new catalysts.

A complete overview of the results obtained at each step of the optimisation project is given in Fig. 4E, overall tripling the initial TON_DMM_ value of 786 to a final value of 2761. As illustrated, the standard deviation (SD) of TON_DMM_ tends to increase with increasing mean value of TON_DMM_ (Fig. 4E), which might be a joint effect from decreasing *n*_cat_ and increasing both *n*_add_ and *V* over the course of the optimisation project. Decreasing *n*_cat_ and increasing *V* is equivalent to reducing the concentration of the catalyst, which presumably renders the system more susceptible to random disturbances by catalyst deactivation, thereby providing an explanation for the observed increase of the standard deviation. Simple Spearman rank correlation analysis of the relationship between SD(TON_DMM_), SD(TON_MF_) and the process parameters *X*_*i*_ supports this hypothesis by revealing a negative and a positive association of SD(TON_DMM_) with *n*_cat_ and *V*, respectively (ESI Table 26†).

Another interesting result in Fig. 4E refers to the independent replication error. The linear design of the second DoE includes two independent settings of the 20% reference as centre points with each point measured as triple replicate (ESI Tables 11 and 17†). This sextet from the second DoE (Fig. 4C, entry RS1) excellently matches the 20% relaxation triple (Fig. 4B, entry 2) indicating good repeatability and reliability of the system (Fig. 4E, ESI Tables 27 and 28†). In a similar way, the best candidate from the second DoE (Fig. 4C, entry 4) has been independently replicated when running the second relaxation sequence (Fig. 4D, entry RS2) and both replicates turned out identical within the experimental error (Fig. 4E, ESI Tables 29 and 30†).

### Technical adaption

The results of the non-biased mathematical modelling approach presented in this study revealed that a better catalytic performance is *inter alia* strongly correlated to a combination of lower catalyst loadings and higher reaction volumes. The heuristic interpretation of this finding indicates that the poor solubility of hydrogen gas in methanol and mass transfer might be limiting factors within our experimental setup. We anticipated that a larger reaction volume, a higher surface to volume ratio and improved mixing would lead to better mass transfer and thus designed an experimental setup accordingly to enhance the catalytic performance. With this upscale autoclave, the TON for DMM increased to 3874, while the TON for MF reached a value of 1445, resulting in a higher selectivity toward DMM (ESI Table 21†). The result of this technical adaption shows that while the reaction is already optimised to a high degree with respect to reaction parameters, further improvements can be expected by focusing on engineering aspects of the reaction setup.

## Conclusion

We demonstrated the power of multivariate optimisation for catalytic processes over the usually applied cumbersome one-factor-at-a-time method. In the homogeneously catalysed transformation of CO_2_ to DMM (dimethoxymethane) and MF (methyl formate) using the ruthenium-triphos complex [Ru(*N*-triphos^Ph^)(tmm)], the TON (turnover number) for DMM was drastically increased to 2761 (with it: TON_MF_ 1769) by an easy-to-use algorithmic workflow combined with only a small number of catalytic experiments. Given the complexity of the transformation, which depends on seven parameters, conventional OFAT screening techniques would have been very costly and time-consuming, with uncertain outcome.

Starting from catalytic data using RF (random forest) for empirical model building, an experimental subspace was identified and subsequently augmented to render the effects of a first set of three process factors estimable. Modelling and optimisation, followed by relaxation led to a sequence of relaxation trials with one candidate assumed to be locally optimal. With this candidate as reference, a linear design of the remaining four variables yielded another substantial improvement. Relaxation of the second design further enhanced the catalytic performance, thereby reaching the technical limits of the setup.

The optimised conditions were used in a specifically designed experimental setup and the highest TON for DMM of 3874 (with it: TON_MF_ 1445) was obtained, which is, to the best of our knowledge, the by far highest value reported in the investigated catalysis.

## Methods

### Theoretical introduction

Experimental design (design of experiments, DoE) methods can be used to study the joint effects of several parameters *X*: *X*_1_, *X*_2_, …, *X*_I_ on response *Y*.^26^,^42^,^43^ This can formally be written as:

1 *Y* = *f*(*X*) + *ε*

*f*() denotes the true, however unknown function, linking the responses *Y* with the process conditions *X*: *X*_1_, *X*_2_, …, *X*_I_, whereas *ε* is a random element taken from a normal distribution with variance *σ*^2^, *ε* ∼ *N*(0,*σ*^2^) to account for experimental uncertainties. Conceptually, nature evaluates in an experiment the function *f*() known to her only at reaction conditions *X*, then adds some random noise *ε* and returns the experimental results *Y* [eqn (1)].

Under the weak assumption that *f*() is smooth and continuous, *f*() can be locally approximated as polynomial parametric surrogates, *g*_1,2,3_(), of increasing complexity, formally:2
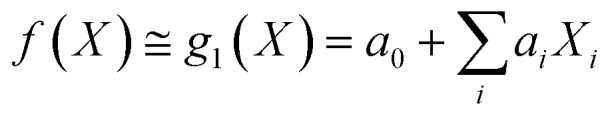

3
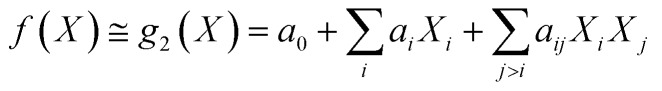

4
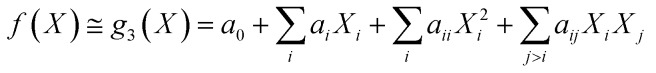



These are linear [eqn (2)], bilinear [eqn (3)] or quadratic [eqn (4)] parametric surrogates of the true function *f*(). With the experimental values *Y*, *X*_1_, *X*_2_, …, *X*_I_ available, the unknown parameters *a*_*i*_, *a*_*ii*_, *a*_*ij*_ can be estimated from the data using ordinary least squares (OLS).^46^

Given process factors *X*_1_, *X*_2_, …, *X*_I_, their ranges *X*_*i*_ ∈ {LB,UB} with LB, UB denoting the lower and upper bounds of the process factors *X*_*i*_ and, depending on the expected complexity, the parametric form of the surrogate model, the design points *X* (experimental design) optimally supporting the chosen model can be calculated. However, in an early project phase it is often unclear which factors *X*_*i*_ and ranges should be chosen and what levels of complexity must be assumed for the domain under investigation. Therefore, DoE can benefit from experimental data analysis, with the latter helping to answer the questions arising in the former.

After first optimisation by an experimental design, ascending in the direction of maximal improvement can be easily achieved by increasing (relaxing) the experimental space in discrete steps and by solving a maximisation problem subject to a sequence of hypercubical constraints, that is

5 max(*g*(*X*)) subject to LB – *k*Δ*X* < *X* < UB + *k*Δ*X*

with Δ*X* being the step size of the relaxation, here taken to be 10% of the initial factor ranges, that is Δ*X* = 0.1[UB – LB], and LB, UB denoting the lower and upper bounds of the process factors *X*_*i*_. Varying *k* = 1, 2, …, K leads to a sequence of relaxation trials 
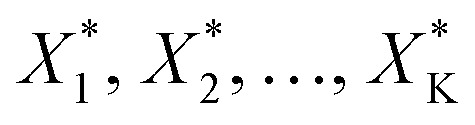
 to be realised in the lab.

### Additional information

The detailed description of all experiments, the performed multivariate analysis, the spectroscopic data of compounds as well as the NMR spectra of compounds and catalysis samples can be found in the ESI.† In order to improve comprehensibility, simplified names were used in some cases rather than using exact IUPAC names.

All calculations were done using the statistical software R.^47^ Random forest modelling was performed with the R-package ‘randomForest’.^48^ Experimental designs were calculated with the D-optimal criterion of the function optFederov() in the R-package ‘AlgDesign’.^49^ Optimisation was achieved with the augmented Lagrange method from the R-package ‘Rsolnp’.^50^ Graphics were produced with the R-package ‘lattice’.^51^

All catalyses and the corresponding analyses were performed following a procedure previously reported by our group.^27^ The catalysis was also performed in the absence of a catalyst, a co-catalyst or both to demonstrate the need of the catalytic system for the formation of DMM and MF. In all cases, no significant conversions for both of these compounds were observed (ESI†).

### Reproduction of modelling results

The R-code used as well as the catalytic data analysed (Excel sheet) are available online. The multivariate analysis can be reproduced by following the instructions in the ESI.†

## Author contributions

M. Siebert and G. K. designed the experiments. M. Siebert performed the experiments. G. K. performed computer modelling and analysis of the data. All authors contributed to the interpretation of the data. The manuscript was co-written by M. Siebert and G. K. and all authors contributed to the manuscript. O. T. supervised the project.

## Conflicts of interest

There are no conflicts to declare.

## Supplementary Material

Supplementary information

Supplementary information

Supplementary information

Infographic
