# Supplementary material for: Identifying high-performance catalytic conditions for carbon dioxide reduction to dimethoxymethane by multivariate modelling
Source: Chem Sci. 2019 Oct 24;10(45):10466–74. doi: 10.1039/c9sc04591k (PMC7012071; doi:10.1039/c9sc04591k)
Supplement: Supplementary file 3 [file SC-010-C9SC04591K-s003.pdf]

Electronic Supplementary Material (ESI) for Chemical Science.
This journal is © The Royal Society of Chemistry 2019

Supporting Information

Identifying high-performance catalytic conditions for the carbon dioxide reduction to dimethoxymethane by multivariate modelling

Max Siebert, Gerhard Krennrich, Max Seibicke, Alexander F. Siegle, and Oliver Trapp

Contents

1 Materials and Methods **S5**
1.1 Preliminary Remarks S5
1.1.1 General Procedures S5
1.1.2 Solvents and Reagents S5
1.2 Analytical Methods S6
1.2.1 NMR Spectroscopy S6
1.2.2 Mass Spectrometry S6
1.2.3 Vibrational Spectroscopy S7
1.2.4 Elemental Analysis S7

2 Synthetic Procedures **S8**
2.1 Synthesis of the Ligand Precursor S8
2.1.1 Bis(hydroxymethyl)diphenylphosphonium chloride **SI-2** S8
2.2 Synthesis of the Ligand S10
2.2.1 *N*-triphos^{Ph} **SI-3** S10
2.3 Synthesis of the Complex S12
2.3.1 [Ru(*N*-triphos^{Ph})(tmm)] **SI-4** S12

3 Multivariate Modeling **S14**

4 Experimental Data **S17**

5 Catalytic Investigations **S26**
5.1 General Procedure for Hydrogenation Reactions S26
5.2 References S31
5.3 First DoE S33
5.3.1 First Triple S33

5.3.2 Second Triple . . . S35
5.3.3 Third Triple . . . S37
5.3.4 Fourth Triple . . . S39
5.3.5 Fifth Triple . . . S41
5.3.6 Sixth Triple . . . S43
5.4 First Relaxation . . . S45
5.4.1 10 % Relaxation . . . S45
5.4.2 20 % Relaxation . . . S47
5.4.3 30 % Relaxation . . . S49
5.5 Second DoE . . . S51
5.5.1 Replication: Best Hit First Relaxation . . . S51
5.5.2 First Triple . . . S53
5.5.3 Second Triple . . . S55
5.5.4 Third Triple . . . S57
5.5.5 Fourth Triple . . . S59
5.5.6 Fifth Triple . . . S61
5.5.7 Replication: Best Hit First Relaxation . . . S63
5.6 Second Relaxation . . . S65
5.6.1 Replication: Best Hit Second DoE . . . S65
5.6.2 25 % Relaxation . . . S67
5.6.3 50 % Relaxation . . . S69
5.7 Upscale Reactions . . . S71
5.8 Background Measurements . . . S73
5.9 Overview of Catalysis Results . . . S79

6 Additional Information **S83**
6.1 Simple Spearman Rank Correlation Analysis . . . S83
6.2 Replication Error . . . S83

7 Appendix **S85**
7.1 NMR Spectra of the Ligand Precursor . . . S85
7.1.1 Bis(hydroxymethyl)diphenylphosphonium chloride **SI-2** . . . S85

7.2 NMR Spectra of the Ligand S87
7.2.1 *N*-triphos^{Ph} **SI-3** S87
7.3 NMR Spectra of the Complex S89
7.3.1 [Ru(*N*-triphos^{Ph})(tmm)] **SI-4** S89

List of Abbreviations **S91**

Bibliography **S93**

1 Materials and Methods

1.1 Preliminary Remarks

1.1.1 General Procedures

Air and moisture sensitive syntheses were carried out within an argon atmosphere under exclusion of air and moisture. The argon gas was dried by silica gel and molecular sieve (4 Å). All glassware was flame-dried prior to use and standard Schlenk techniques were applied. The given yields refer to isolated and purified products.

1.1.2 Solvents and Reagents

Toluene was dried in a MB SPS-800 system and stored under nitrogen. All other anhydrous solvents used were bought and directly used or stored under exclusion of air and moisture. Degassing was accomplished by at least three Freeze-Pump-Thaw cycles. The anhydrous and degassed solvents were stored under argon over molecular sieve (3 Å). Argon gas (Ar 5.0), hydrogen gas (H_2 5.0) and carbon dioxide gas (CO_2 4.5) were purchased from Air Liquide Deutschland GmbH. Argon gas was purified as described above, whereas hydrogen gas and carbon dioxide gas were used without further purification. All other solvents and chemicals were purchased from manufacturing and trading companies (abcr GmbH, Acros Organics b.v.b.a., Sigma-Aldrich Co. LLC., Strem Chemicals Inc. and TCI Europe N.V.) and stored according to the respective instructions. Isolated compounds were stored under argon and, if needed, at 5 or -20 °C.

1.2 Analytical Methods

1.2.1 NMR Spectroscopy

NMR spectra were recorded on a 400 MHz Bruker Avance III HD spectrometer and a 400 MHz Bruker Avance III HD spectrometer with a CryoProbe Prodigy. NMR spectra of air and moisture sensitive compounds were measured using J. Young NMR tubes. The solvent residual signals were used for internal calibration.[1] Chemical shifts δ are reported in ppm and coupling constants J in Hz. The different multiplicities are defined by s (singlet), d (doublet) and m (multiplet) or by combinations of these. In few cases, the peak shape is specified by b (broad) in addition to the description of the multiplicity. The assignments in ^{13}C NMR and ^{31}P NMR spectra refer to proton decoupled experiments. The assignment of all signals was realized by two-dimensional NMR spectroscopy (^{1}H-^{1}H-COSY, ^{1}H-^{13}C-HSQC-ME and ^{1}H-^{13}C-HMBC experiments). Atom numbering for NMR assignments is not based on IUPAC nomenclature.

For the quantification experiments, ^{1}H NMR spectra were obtained with a pulse width of 3.6 µs (30° flip angle), an acquisition time of 4.1 s and a delay time of 1.0 s or a pulse width of 5.0 µs (30° flip angle), an acquisition time of 5.1 s and a delay time of 1.0 s.

1.2.2 Mass Spectrometry

Mass spectrometric measurements were performed on a Thermo Finnigan LTQ Ultra FT-ICR mass spectrometer. The mode (positive / negative) of the experiment, the solvent used to dissolve the sample and the method of injection together with potential solvents are listed. The *m/z* value of the most abundant or characteristic peak is given in comparison to the calculated *m/z* value. In case of characteristic peaks, the abundance is given in percent. If isotopic patterns are pronounced, the most intensive peak is indicated. Molecular ions are abbreviated as *M*.

[1] G. R. Fulmer et al., *Organometallics*, 2010, **29**, 2176–2179.

1.2.3 Vibrational Spectroscopy

IR spectra were recorded on a Thermo Scientific Nicolet 700 ATR-FT-IR spectrometer using the purified products without further modification. The background transmission was measured before the sample measurement and subtracted. Wave numbers $\tilde{\nu}$ are given in cm^{-1}.

1.2.4 Elemental Analysis

Elemental analyses were performed using a Elementar vario EL or a Elementar vario MICRO cube instrument.

2 Synthetic Procedures

2.1 Synthesis of the Ligand Precursor

2.1.1 Bis(hydroxymethyl)diphenylphosphonium chloride SI-2

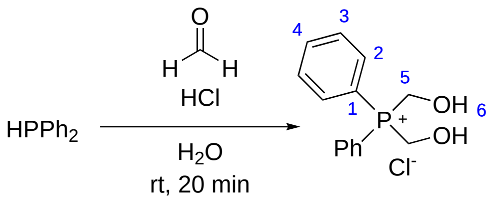

SI-1 **SI-2**

Compound **SI-2** is literature known[2] and was synthesized according to a reported procedure.[3]

Diphenylphosphine **SI-1** (9.31 g, 8.70 mL, 50.0 mmol, 1.00 eq.) was stirred with degassed hydrochloric acid (37 *wt*%, 7.04 mL, 85.0 mmol, 1.70 eq.) and degassed aqueous formaldehyde solution (37 *wt*%, 14.5 mL, 195 mmol, 3.90 eq.) for 20 min at room temperature. The volatile compounds were removed *in vacuo*. Precipitation was induced by addition of acetone (5 mL). The resulting colorless solid was filtered and washed with cold Et_2O ($3 \cdot 10$ mL). The product **SI-2** (12.9 g, 45.6 mmol, 91 %) was isolated as a colorless solid.

[2] J. Fawcett, P. A. T. Hoye, R. D. W. Kemmitt, D. J. Law and D. R. Russell, *J. Chem. Soc., Dalton Trans.* 1993, 2563–2568.

[3] A. Phanopoulos, N. J. Brown, A. J. P. White, N. J. Long and P. W. Miller, *Inorg. Chem.* 2014, **53**, 3742–3752.

^{1}H NMR (CDCl$_3$, 400 MHz, 298 K):
δ = 5.06 (s, 4H, H^5), 7.62-7.67 (m, 4H, H^3), 7.77-7.83 (m, 6H, H^{2,4}).

^{13}C{^{1}H} NMR (CDCl$_3$, 101 MHz, 298 K):
δ = 54.7 (d, $^1J_{C\text{-}P}$ = 55.9 Hz, 2C, C^5), 116.2 (d, $^1J_{C\text{-}P}$ = 73.2 Hz, 2C, C^1), 130.4 (d, $^3J_{C\text{-}P}$ = 10.8 Hz, 4C, C^3), 133.4 (d, $^2J_{C\text{-}P}$ = 8.5 Hz, 4C, C^2), 135.3 (d, $^4J_{C\text{-}P}$ = 3.1 Hz, 2C, C^4).

^{31}P{^{1}H} NMR (CDCl$_3$, 162 MHz, 298 K):
δ = 13.39 (s, 1P).

HR-MS (ESI$^+$, CHCl$_3$, FIA: ACN/H$_2$O):

[M-CH$_2$O-Cl]$^+$: $C_{13}H_{14}OP^+$	calculated *m/z*	217.0777
	found *m/z*	217.0776

IR (FT-ATR):
$\tilde{\nu}$ = 3161, 3094, 3065, 3021, 2929, 2887, 2877, 2821, 2607, 1588, 1489, 1437, 1418, 1311, 1288, 1238, 1210, 1191, 1118, 1112, 1059, 1051, 934, 891, 844, 752, 740, 688.

EA (vario MICRO):

$C_{14}H_{16}ClO_2P$	calculated [%]	C 59.48; H 5.70
	found [%]	C 58.72; H 5.69

2.2 Synthesis of the Ligand

2.2.1 *N*-triphos^{Ph} SI-3

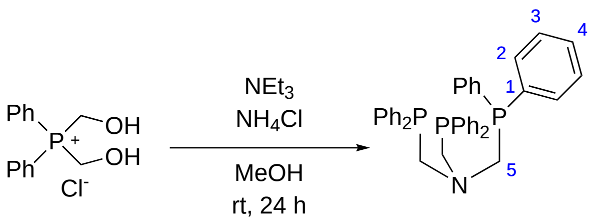

SI-2 **SI-3**

Compound **SI-3** is literature known[4] and was synthesized according to a reported procedure.[5]

Bis(hydroxymethyl)diphenylphosphonium chloride **SI-2** (6.68 g, 23.6 mmol, 1.00 eq.) was dissolved in anhydrous degassed MeOH (25 mL) and anhydrous degassed NEt_3 (6.55 mL, 47.3 mmol, 2.00 eq.) was added. The solution was stirred for 1 h at room temperature. A solution of NH_4Cl (417 mg, 7.80 mmol, 0.33 eq.) in anhydrous degassed MeOH (25 mL) and additional anhydrous degassed NEt_3 (6.55 mL, 47.3 mmol, 2.00 eq.) was added. The reaction mixture was stirred for 24 h at room temperature. The colorless precipitate was filtered and washed with anhydrous degassed MeOH (4 · 15 mL). The product **SI-3** (4.30 g, 7.03 mmol, 89 %) was isolated as a colorless solid.

^{1}H NMR ($CDCl_3$, 400 MHz, 298 K):

$\delta =$ 3.79 (d, $^{2}J_{H\text{-}P}$ = 3.6 Hz, 6H, H^{5}), 7.18-7.29 (m, 18H, $H^{2/3,4}$), 7.33-7.39 (m, 12H, $H^{2/3}$).

^{13}C{^{1}H} NMR ($CDCl_3$, 101 MHz, 298 K):

$\delta =$ 59.9-60.2 (m, 3C, C^{5}), 128.4 (d, $^{2/3}J_{C\text{-}P}$ = 6.9 Hz, 12C, $C^{2/3}$), 128.6 (s, 6C, C^{4}), 133.3 (d, $^{2/3}J_{C\text{-}P}$ = 18.2 Hz, 12C, $C^{2/3}$), 137.9 (d, $^{1}J_{C\text{-}P}$ = 12.2 Hz, 6C, C^{1}).

^{31}P{^{1}H} NMR ($CDCl_3$, 162 MHz, 298 K):

$\delta =$ -28.96 (s, 3P).

[4] G. Märkl and G. Y. Jin, *Tetrahedron Lett.* 1981, **22**, 1105–1108.
[5] J. Lloret Fillol, A. Kruckenberg, P. Scherl, H. Wadepohl and L. H. Gade, *Chem. Eur. J.* 2011, **17**, 14047–14062.

HR-MS (ESI$^+$, $CHCl_3$, FIA: ACN/H_2O):

$[M+O_2\text{-}PPh_2]^+$: $C_{27}H_{26}NO_2P_2^+$	calculated *m/z*	458.1433
	found *m/z*	458.1431

IR (FT-ATR):

$\tilde{\nu}$ = 3067, 3048, 3014, 2998, 2927, 2903, 2843, 2802, 2789, 2779, 1952, 1885, 1876, 1583, 1568, 1478, 1429, 1419, 1383, 1298, 1276, 1230, 1211, 1178, 1097, 1073, 1067, 1052, 1025, 934, 883, 868, 863, 757, 745, 739, 691.

EA (vario MICRO):

$C_{39}H_{36}NP_3$	calculated [%]	C 76.59; H 5.93; N 2.29
	found [%]	C 75.64; H 5.96; N 2.31

2.3 Synthesis of the Complex

2.3.1 [Ru(*N*-triphos^{Ph})(tmm)] SI-4

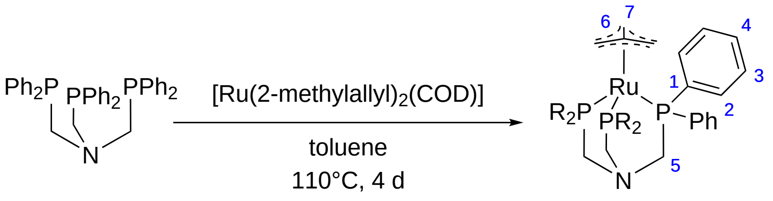

SI-3 **SI-4**

Compound **SI-4** is literature known and was synthesized according to a reported procedure.[3]

N-triphos^{Ph} **SI-3** (4.30 g, 7.03 mmol, 1.00 eq.) and [Ru(2-methylallyl)$_2$(COD)] (2.25 g, 7.03 mmol, 1.00 eq.) were dissolved in anhydrous degassed toluene (50 mL). The solution was stirred for 4 d at 110 °C. The volatile compounds were removed *in vacuo* by half inducing precipitation. The supernatant solution was removed using a filter cannula. The solid was washed with anhydrous degassed toluene (1 · 15 mL, 2 · 7.5 mL). The product **SI-4** (3.36 g, 4.38 mmol, 62 %) was isolated as an off-white solid.

^{1}H NMR (CDCl$_3$, 400 MHz, 298 K):
δ = 1.68 (s, 6H, H^7), 3.86 (s, 6H, H^5), 6.88-6.99 (m, 24H, H^{2,3}), 7.04-7.10 (m, 6H, H^4).

^{13}C{^{1}H} NMR (CDCl$_3$, 101 MHz, 298 K):
δ = 43.9-44.2 (m, 3C, C^7), 53.8-54.2 (m, 3C, C^5), 105.5 (bs, 1C, C^6), 127.5-127.6 (m, 12C, C$^{2/3}$), 127.7 (s, 6C, C^4), 132.5-132.7 (m, 12C, C$^{2/3}$), 141.1-141.5 (m, 6C, C^1).

^{31}P{^{1}H} NMR (CDCl$_3$, 162 MHz, 298 K):
δ = 18.42 (s, 3P).

HR-MS (ESI$^+$, CHCl$_3$, FIA: ACN/H$_2$O):

[M+H]$^+$: $C_{43}H_{43}NP_3Ru^+$	calculated *m/z*	768.1646
	found *m/z*	768.1655

IR (FT-ATR):
$\tilde{\nu}$ = 3070, 3050, 3032, 2986, 2881, 2804, 2789, 2779, 1972, 1872, 1804, 1585, 1572, 1480, 1432, 1411, 1385, 1317, 1299, 1271, 1180, 1091, 1083, 1070, 996, 934, 894, 861, 853, 842, 736, 695.

EA (vario EL):

$C_{43}H_{42}NP_3Ru$	calculated [%]	C 67.35; H 5.52; N 1.83
	found [%]	C 67.35; H 5.47; N 1.88

3 Multivariate Modeling

All calculations were done using the statistical software R.[6] Random forest modeling was performed with the R-package 'randomForest'.[7,8] Experimental designs were calculated with the D-optimal criterion of the function optFederov() in the R-package 'AlgDesign'.[9] Optimization was achieved with the augmented Lagrange method from the R-package 'Rsolnp'.[10] Graphics were produced with the R-package 'lattice'.[11,12]

The whole optimization process can be divided into three major steps, namely experimental data analysis, DoE1 and DoE2. Each of the two steps DoE1 and DoE2 can be further divided into three sub-steps comprising design creation, OLS modeling and constrained optimization.

All steps of the project can be reproduced with the data file 'all.data.xlsx' and the R-script 'all.R.code.r'. No special R knowledge is required to run the code and for reproducing the results of the individual steps. With some basic R-knowledge, it should be easily possible to adapt the code to the needs of other projects. All additional packages (libraries) that have to be added to the standard user library are mentioned within the R-script 'all.R.code.r'.

[6] R Core Team, *R: A Language and Environment for Statistical Computing*, https://www.R-project.org, 2017.
[7] A. Liaw and M. Wiener, *R News*, 2002, **2**, 18–22.
[8] L. Breiman, A. Cutler, A. Liaw and M. Wiener, *randomForest: Breiman and Cutler's Random Forests for Classification and Regression*, https://CRAN.R-project.org/package=randomForest, 2018.
[9] B. Wheeler, *AlgDesign: Algorithmic Experimental Design*, https://CRAN.R-project.org/package=AlgDesign, 2014.
[10] A. Ghalanos and S. Theussl, *Rsolnp: General Non-linear Optimization Using Augmented Lagrange Multiplier Method*, https://CRAN.R-project.org/package=Rsolnp, 2015.
[11] D. Sarkar, *Lattice*, Springer-Verlag, New York, 2008.
[12] D. Sarkar, *lattice: Trellis Graphics for R*, https://CRAN.R-project.org/package=lattice, 2018.

These packages have to be downloaded prior to the calculations (namely 'lattice',[11,12] 'randomForest',[7,8] 'pals',[13] 'viridis',[14] 'readxl',[15] 'AlgDesign',[9] 'MASS',[16] 'Rsolnp'[10]).
To calculate the different steps of the optimization process, the user has to run the individual steps in the given order. Therefore, the respective code framed by the descriptions given below has to be marked and run.

Important: The user has to make sure to put the R-script 'all.R.code.r' in the folder 'R-code' with the path C:\R-code\and to put the data file 'all.data.xlsx' in the subfolder 'data' with the path C:\R-code\data\. If a different location for the folder was chosen, the path in line #5 of the R-script has to be adjusted accordingly.

Referring to the R-code and the comments used therein, the different steps can be characterized:

1. **Experimental data analysis:** The code, starting in line #1 to entry 'End 1', reads the experimental data of dimension 144 x 10 from the Excel sheet 'historical.data', performs random forest modeling and visualizes the modeling results of the major response ton.dmm as scatter and trellis plots.

2. **DoE1**

    a) The code, starting at entry 'Start 2a' to entry 'End 2a', augments the set of nine candidates with ton.dmm>400 by another six d-optimal candidate points and reports the results to the console along with the condition number of the augmented design.

    b) The code, starting at entry 'Start 2b' to entry 'End 2b', analyzes the data with stepwise ordinary least squares (stepOLS) using the Bayesian Information Criterion (BIC) for model building. Modeling results for the major response, ton.dmm, are reported similar to those obtained for experimental data analysis (plot observed ton.dmm versus predicted ton.dmm and response surface trellis plots).

[13] K. Wright, *pals: Color Palettes, Colormaps, and Tools to Evaluate Them*, https://CRAN.R-project.org/package=pals, 2018.
[14] S. Garnier, *viridis: Default Color Maps from 'matplotlib'*, https://CRAN.R-project.org/package=viridis, 2018.
[15] H. Wickham et al., *readxl: Read Excel Files*, https://CRAN.R-project.org/package=readxl, 2019.
[16] B. Ripley et al., *MASS: Support Functions and Datasets for Venables and Ripley's MASS*, https://CRAN.R-project.org/package=MASS, 2018.

c) The code, starting at entry 'Start 2c' to entry 'End 2c', maximizes ton.dmm under relaxed hypercubical constraints, here at step size of 10, 20, 30 % and reports the results to the console. The values reported for ton.dmm and ton.mf are OLS-based expectations against which experimental results can be compared.

3. **DoE2**

   a) The code, starting at entry 'Start 3a' to entry 'End 3a', creates a small, d-optimal design comprising the factors T (80-100), p.H2 (80-100), p.CO2 (15-25) and t.H (16-20) aiming at the linear effects of the four process parameters. The design is reported to the console together with its condition number.

   b) With the code, starting at entry 'Start 3b' to entry 'End 3b', DoE2 results are modeled with stepOLS and modeling results are reported similar to 2b.

   c) The code, starting at entry 'Start 3c' to entry 'End 3c', follows the same rationale as 2c, however with modified step size of 0, 25 and 50 %.

The simplified parameter designations used here do not correspond with the designations used in the text or the remaining SI that have been optimized for legibility. Nevertheless, the parameter designation is unambiguous. For the sake of clarity, the use of units in the mathematical approach has been omitted.

4 Experimental Data

The data obtained from a comprehensive screening of the catalysis process parameters temperature, partial pressure of H_2 and CO_2, time, additive as well as catalyst and additive loading were used for the multivariate analysis approach.[17] The complete data is given in Supplementary Table 1. While the TON values given here were rounded, for the multivariate analysis the exact values were taken, which were calculated with the values and formulas given in the original literature.[17] For the sake of the statistical evaluation, significant digits were not taken into account in the SI, while the values given in the manuscript were rounded to three decimal places for the substance quantities and the reaction volume.

The entries 142-144 of Supplementary Table 1 refer to an additional triple experiment that was performed to investigate the dependency of catalyst deactivation on concentration effects. This triple was added to the experimental data mentioned above to account for the effect of different reaction volumes in the statistical analysis.

[17] M. Siebert, M. Seibicke, A. F. Siegle, S. Kräh and O. Trapp, *J. Am. Chem. Soc.* 2019, **141**, 334–341.

Supplementary Table 1: Overview of the experimental data from a previous publication of our group used for the random forest algorithm.[17]

	entry	Catalysis Process Parameters								Responses	
		T (°C)	p_{H2} (bar)	p_{CO2} (bar)	t (h)	additive	n_{cat} (µmol)	n_{add} (µmol)	V_{MeOH} (mL)	TON DMM	TON MF
	1	20	60	20	18	$Al(OTf)_3$	1.5	6.25	0.5	4	49
	2	20	60	20	18	$Al(OTf)_3$	1.5	6.25	0.5	7	46
	3	20	60	20	18	$Al(OTf)_3$	1.5	6.25	0.5	13	59
	4	70	60	20	18	$Al(OTf)_3$	1.5	6.25	0.5	154	165
	5	70	60	20	18	$Al(OTf)_3$	1.5	6.25	0.5	156	163
	6	70	60	20	18	$Al(OTf)_3$	1.5	6.25	0.5	181	156
	7	80	60	20	18	$Al(OTf)_3$	1.5	6.25	0.5	269	108
	8	80	60	20	18	$Al(OTf)_3$	1.5	6.25	0.5	289	115
	9	80	60	20	18	$Al(OTf)_3$	1.5	6.25	0.5	319	114
	10	85	60	20	18	$Al(OTf)_3$	1.5	6.25	0.5	314	96
	11	85	60	20	18	$Al(OTf)_3$	1.5	6.25	0.5	329	91
	12	85	60	20	18	$Al(OTf)_3$	1.5	6.25	0.5	329	92
	13	90	60	20	18	$Al(OTf)_3$	1.5	6.25	0.5	296	80
Temperature	14	90	60	20	18	$Al(OTf)_3$	1.5	6.25	0.5	313	88
	15	90	60	20	18	$Al(OTf)_3$	1.5	6.25	0.5	320	88
	16	95	60	20	18	$Al(OTf)_3$	1.5	6.25	0.5	263	69
	17	95	60	20	18	$Al(OTf)_3$	1.5	6.25	0.5	271	76
	18	95	60	20	18	$Al(OTf)_3$	1.5	6.25	0.5	280	74
	19	100	60	20	18	$Al(OTf)_3$	1.5	6.25	0.5	201	70
	20	100	60	20	18	$Al(OTf)_3$	1.5	6.25	0.5	205	59

Continued on next page...

Supplementary Table 1: Overview of the experimental data from a previous publication of our group used for the random forest algorithm.[17] (continued)

	entry	Catalysis Process Parameters								Responses	
		T (°C)	p_{H2} (bar)	p_{CO2} (bar)	t (h)	additive	n_{cat} (µmol)	n_{add} (µmol)	V_{MeOH} (mL)	TON DMM	TON MF
	21	100	60	20	18	Al(OTf)$_3$	1.5	6.25	0.5	219	69
	22	110	60	20	18	Al(OTf)$_3$	1.5	6.25	0.5	111	48
	23	110	60	20	18	Al(OTf)$_3$	1.5	6.25	0.5	118	51
	24	110	60	20	18	Al(OTf)$_3$	1.5	6.25	0.5	129	58
	25	120	60	20	18	Al(OTf)$_3$	1.5	6.25	0.5	47	41
	26	120	60	20	18	Al(OTf)$_3$	1.5	6.25	0.5	60	43
	27	120	60	20	18	Al(OTf)$_3$	1.5	6.25	0.5	61	42
H_2 pressure	28	90	40	20	18	Al(OTf)$_3$	1.5	6.25	0.5	241	67
	29	90	40	20	18	Al(OTf)$_3$	1.5	6.25	0.5	243	70
	30	90	40	20	18	Al(OTf)$_3$	1.5	6.25	0.5	266	70
	31	90	50	20	18	Al(OTf)$_3$	1.5	6.25	0.5	254	76
	32	90	50	20	18	Al(OTf)$_3$	1.5	6.25	0.5	289	80
	33	90	50	20	18	Al(OTf)$_3$	1.5	6.25	0.5	293	72
	34	90	70	20	18	Al(OTf)$_3$	1.5	6.25	0.5	291	87
	35	90	70	20	18	Al(OTf)$_3$	1.5	6.25	0.5	332	80
	36	90	70	20	18	Al(OTf)$_3$	1.5	6.25	0.5	334	81
	37	90	80	20	18	Al(OTf)$_3$	1.5	6.25	0.5	292	77
	38	90	80	20	18	Al(OTf)$_3$	1.5	6.25	0.5	333	86
	39	90	80	20	18	Al(OTf)$_3$	1.5	6.25	0.5	352	85
	40	90	90	20	18	Al(OTf)$_3$	1.5	6.25	0.5	348	91

Continued on next page...

Supplementary Table 1: Overview of the experimental data from a previous publication of our group used for the random forest algorithm.[17] (continued)

	entry	Catalysis Process Parameters								Responses	
		T (°C)	p_{H2} (bar)	p_{CO2} (bar)	t (h)	additive	n_{cat} (µmol)	n_{add} (µmol)	V_{MeOH} (mL)	TON DMM	TON MF
	41	90	90	20	18	Al(OTf)$_3$	1.5	6.25	0.5	358	85
	42	90	90	20	18	Al(OTf)$_3$	1.5	6.25	0.5	383	102
	43	90	100	20	18	Al(OTf)$_3$	1.5	6.25	0.5	303	82
	44	90	100	20	18	Al(OTf)$_3$	1.5	6.25	0.5	336	93
	45	90	100	20	18	Al(OTf)$_3$	1.5	6.25	0.5	357	84
CO_2 pressure	46	90	90	5	18	Al(OTf)$_3$	1.5	6.25	0.5	86	8
	47	90	90	5	18	Al(OTf)$_3$	1.5	6.25	0.5	87	8
	48	90	90	5	18	Al(OTf)$_3$	1.5	6.25	0.5	118	9
	49	90	90	10	18	Al(OTf)$_3$	1.5	6.25	0.5	202	34
	50	90	90	10	18	Al(OTf)$_3$	1.5	6.25	0.5	214	38
	51	90	90	10	18	Al(OTf)$_3$	1.5	6.25	0.5	222	34
	52	90	90	15	18	Al(OTf)$_3$	1.5	6.25	0.5	272	65
	53	90	90	15	18	Al(OTf)$_3$	1.5	6.25	0.5	322	71
	54	90	90	15	18	Al(OTf)$_3$	1.5	6.25	0.5	347	69
	55	90	90	25	18	Al(OTf)$_3$	1.5	6.25	0.5	341	109
	56	90	90	25	18	Al(OTf)$_3$	1.5	6.25	0.5	358	94
	57	90	90	25	18	Al(OTf)$_3$	1.5	6.25	0.5	383	100
	58	90	90	30	18	Al(OTf)$_3$	1.5	6.25	0.5	317	101
	59	90	90	30	18	Al(OTf)$_3$	1.5	6.25	0.5	320	113
	60	90	90	30	18	Al(OTf)$_3$	1.5	6.25	0.5	349	111

Continued on next page...

Supplementary Table 1: Overview of the experimental data from a previous publication of our group used for the random forest algorithm.[17] (continued)

	entry	Catalysis Process Parameters								Responses	
		T (°C)	p_{H2} (bar)	p_{CO2} (bar)	t (h)	additive	n_{cat} (µmol)	n_{add} (µmol)	V_{MeOH} (mL)	TON DMM	TON MF
	61	90	90	40	18	$Al(OTf)_3$	1.5	6.25	0.5	259	120
	62	90	90	40	18	$Al(OTf)_3$	1.5	6.25	0.5	278	116
	63	90	90	40	18	$Al(OTf)_3$	1.5	6.25	0.5	287	116
Time at 5 bar CO_2	64	90	90	5	1	$Al(OTf)_3$	1.5	6.25	0.5	20	36
	65	90	90	5	1	$Al(OTf)_3$	1.5	6.25	0.5	21	36
	66	90	90	5	1	$Al(OTf)_3$	1.5	6.25	0.5	21	32
	67	90	90	5	6	$Al(OTf)_3$	1.5	6.25	0.5	95	13
	68	90	90	5	6	$Al(OTf)_3$	1.5	6.25	0.5	95	14
	69	90	90	5	6	$Al(OTf)_3$	1.5	6.25	0.5	102	20
	70	90	90	5	12	$Al(OTf)_3$	1.5	6.25	0.5	91	11
	71	90	90	5	12	$Al(OTf)_3$	1.5	6.25	0.5	97	11
	72	90	90	5	12	$Al(OTf)_3$	1.5	6.25	0.5	130	18
	73	90	90	5	24	$Al(OTf)_3$	1.5	6.25	0.5	69	7
	74	90	90	5	24	$Al(OTf)_3$	1.5	6.25	0.5	93	8
	75	90	90	5	24	$Al(OTf)_3$	1.5	6.25	0.5	94	5
	76	90	90	5	48	$Al(OTf)_3$	1.5	6.25	0.5	30	3
	77	90	90	5	48	$Al(OTf)_3$	1.5	6.25	0.5	38	4
	78	90	90	5	48	$Al(OTf)_3$	1.5	6.25	0.5	57	5
	79	90	90	5	72	$Al(OTf)_3$	1.5	6.25	0.5	12	1
	80	90	90	5	72	$Al(OTf)_3$	1.5	6.25	0.5	15	2

Continued on next page…

Supplementary Table 1: Overview of the experimental data from a previous publication of our group used for the random forest algorithm.[17] (continued)

	entry	T (°C)	p_{H2} (bar)	p_{CO2} (bar)	t (h)	additive	n_{cat} (µmol)	n_{add} (µmol)	V_{MeOH} (mL)	TON DMM	TON MF
	81	90	90	5	72	$Al(OTf)_3$	1.5	6.25	0.5	33	5
	82	90	90	5	168	$Al(OTf)_3$	1.5	6.25	0.5	3	0
	83	90	90	5	168	$Al(OTf)_3$	1.5	6.25	0.5	3	0
	84	90	90	5	168	$Al(OTf)_3$	1.5	6.25	0.5	5	0
Time at 20 bar CO_2	85	90	90	20	1	$Al(OTf)_3$	1.5	6.25	0.5	15	71
	86	90	90	20	1	$Al(OTf)_3$	1.5	6.25	0.5	17	56
	87	90	90	20	1	$Al(OTf)_3$	1.5	6.25	0.5	17	60
	88	90	90	20	6	$Al(OTf)_3$	1.5	6.25	0.5	157	61
	89	90	90	20	6	$Al(OTf)_3$	1.5	6.25	0.5	158	61
	90	90	90	20	6	$Al(OTf)_3$	1.5	6.25	0.5	163	66
	91	90	90	20	12	$Al(OTf)_3$	1.5	6.25	0.5	217	74
	92	90	90	20	12	$Al(OTf)_3$	1.5	6.25	0.5	223	75
	93	90	90	20	12	$Al(OTf)_3$	1.5	6.25	0.5	305	101
	94	90	90	20	24	$Al(OTf)_3$	1.5	6.25	0.5	295	75
	95	90	90	20	24	$Al(OTf)_3$	1.5	6.25	0.5	326	78
	96	90	90	20	24	$Al(OTf)_3$	1.5	6.25	0.5	330	79
	97	90	90	20	48	$Al(OTf)_3$	1.5	6.25	0.5	271	56
	98	90	90	20	48	$Al(OTf)_3$	1.5	6.25	0.5	303	65
	99	90	90	20	48	$Al(OTf)_3$	1.5	6.25	0.5	306	61
	100	90	90	20	72	$Al(OTf)_3$	1.5	6.25	0.5	229	56

Column groups: Catalysis Process Parameters (T through V_{MeOH}); Responses (TON: DMM, MF).

Continued on next page...

Supplementary Table 1: Overview of the experimental data from a previous publication of our group used for the random forest algorithm.[17] (continued)

	entry	Catalysis Process Parameters								Responses	
		T (°C)	p_{H2} (bar)	p_{CO2} (bar)	t (h)	additive	n_{cat} (µmol)	n_{add} (µmol)	V_{MeOH} (mL)	TON DMM	TON MF
	101	90	90	20	72	$Al(OTf)_3$	1.5	6.25	0.5	238	54
	102	90	90	20	72	$Al(OTf)_3$	1.5	6.25	0.5	262	55
	103	90	90	20	168	$Al(OTf)_3$	1.5	6.25	0.5	168	44
	104	90	90	20	168	$Al(OTf)_3$	1.5	6.25	0.5	172	48
	105	90	90	20	168	$Al(OTf)_3$	1.5	6.25	0.5	191	48
Catalyst loading	106	90	90	20	18	$Al(OTf)_3$	0.1875	0.78125	0.5	669	1375
	107	90	90	20	18	$Al(OTf)_3$	0.1875	0.78125	0.5	670	1115
	108	90	90	20	18	$Al(OTf)_3$	0.1875	0.78125	0.5	846	1377
	109	90	90	20	18	$Al(OTf)_3$	0.375	1.5625	0.5	725	521
	110	90	90	20	18	$Al(OTf)_3$	0.375	1.5625	0.5	785	527
	111	90	90	20	18	$Al(OTf)_3$	0.375	1.5625	0.5	849	552
	112	90	90	20	18	$Al(OTf)_3$	0.75	3.125	0.5	443	159
	113	90	90	20	18	$Al(OTf)_3$	0.75	3.125	0.5	510	240
	114	90	90	20	18	$Al(OTf)_3$	0.75	3.125	0.5	630	219
	115	90	90	20	18	$Al(OTf)_3$	2.25	9.375	0.5	180	44
	116	90	90	20	18	$Al(OTf)_3$	2.25	9.375	0.5	203	50
	117	90	90	20	18	$Al(OTf)_3$	2.25	9.375	0.5	219	46
	118	90	90	20	18	$Al(OTf)_3$	3.0	12.5	0.5	117	35
	119	90	90	20	18	$Al(OTf)_3$	3.0	12.5	0.5	119	31
	120	90	90	20	18	$Al(OTf)_3$	3.0	12.5	0.5	131	32

Continued on next page...

Supplementary Table 1: Overview of the experimental data from a previous publication of our group used for the random forest algorithm.[17] (continued)

	entry	Catalysis Process Parameters								Responses	
		T (°C)	p_{H2} (bar)	p_{CO2} (bar)	t (h)	additive	n_{cat} (µmol)	n_{add} (µmol)	V_{MeOH} (mL)	TON DMM	TON MF
Additive loading	121	90	90	20	18	$Al(OTf)_3$	0.375	0.9375	0.5	345	633
	122	90	90	20	18	$Al(OTf)_3$	0.375	0.9375	0.5	467	642
	123	90	90	20	18	$Al(OTf)_3$	0.375	0.9375	0.5	527	676
	124	90	90	20	18	$Al(OTf)_3$	0.375	1.25	0.5	455	476
	125	90	90	20	18	$Al(OTf)_3$	0.375	1.25	0.5	456	468
	126	90	90	20	18	$Al(OTf)_3$	0.375	1.25	0.5	683	588
	127	90	90	20	18	$Al(OTf)_3$	0.375	1.875	0.5	644	371
	128	90	90	20	18	$Al(OTf)_3$	0.375	1.875	0.5	705	366
	129	90	90	20	18	$Al(OTf)_3$	0.375	1.875	0.5	745	507
	130	90	90	20	18	$Al(OTf)_3$	0.375	2.1875	0.5	648	387
	131	90	90	20	18	$Al(OTf)_3$	0.375	2.1875	0.5	712	392
	132	90	90	20	18	$Al(OTf)_3$	0.375	2.1875	0.5	906	559
	133	90	90	20	18	$Al(OTf)_3$	0.375	2.5	0.5	699	327
	134	90	90	20	18	$Al(OTf)_3$	0.375	2.5	0.5	755	470
	135	90	90	20	18	$Al(OTf)_3$	0.375	2.5	0.5	789	535
Background	136	90	90	20	18	$Al(OTf)_3$	0.0	0.0	0.5	0	6
	137	90	90	20	18	$Al(OTf)_3$	0.0	0.0	0.5	1	2
	138	90	90	20	18	$Al(OTf)_3$	1.5	0.0	0.5	0	8
	139	90	90	20	18	$Al(OTf)_3$	1.5	0.0	0.5	1	11
	140	90	90	20	18	$Al(OTf)_3$	0.0	6.25	0.5	0	5

Continued on next page...

Supplementary Table 1: Overview of the experimental data from a previous publication of our group used for the random forest algorithm.[17] (continued)

	entry	Catalysis Process Parameters								Responses	
		T (°C)	p_{H2} (bar)	p_{CO2} (bar)	t (h)	additive	n_{cat} (µmol)	n_{add} (µmol)	V_{MeOH} (mL)	TON DMM	TON MF
Volume	141	90	90	20	18	$Al(OTf)_3$	0.0	6.25	0.5	1	5
	142	90	90	20	18	$Al(OTf)_3$	0.375	1.5625	0.25	604	204
	143	90	90	20	18	$Al(OTf)_3$	0.375	1.5625	0.25	616	188
	144	90	90	20	18	$Al(OTf)_3$	0.375	1.5625	0.25	631	195

5 Catalytic Investigations

5.1 General Procedure for Hydrogenation Reactions

All catalyses and the corresponding analyses were performed three times following a procedure previously reported by our group.[17]
The catalyses were performed in stainless steel high-pressure autoclaves (10 mL total volume) using an NMR tube as inset and a small stirring bar for mixing. The catalyst and the additive were suspended in a Schlenk tube in MeOH and the mixture was stirred for 1 h. The autoclave was evacuated and flushed with nitrogen three times before the respective amount of reaction mixture was transferred into the NMR tube inset. The carbon dioxide gas line was purged with CO_2 and the hydrogen gas line was purged with H_2 at least seven times. After the autoclave was pressurized with CO_2, the CO_2 was deposited by cooling the autoclave to -78 °C for 5 min. Then, the autoclave was further pressurized with H_2. The closed autoclave was allowed to warm to room temperature for 10 min. The reaction mixture was stirred at the mentioned temperature for the defined time. The autoclaves were cooled to 0 °C for 15 min before the gas was discharged. The TONs of the catalyses were determined by ^{1}H NMR spectroscopy. A sample of 50 µL of the reaction mixture and 35 µL mesitylene as internal standard were dissolved in 450 µL DCM-d_2.[17]

With the experimental setup tailor-made for the optimized conditions, the catalysis was performed twice. The new autoclave (35 mL total volume) enabled better mixing of the catalysis mixture and enhanced the mass transfer within the catalytic reaction. For this autoclave, the cool-down time at -78 °C was extended to 10 min and the warm-up time at room temperature as well as the cool-down time at 0 °C to 20 min.

The catalysis was also performed in the absence of a catalyst, a co-catalyst or both to demonstrate the need of the catalytic system for the formation of DMM and MF. In all cases, no significant conversions for both of these compounds were observed.

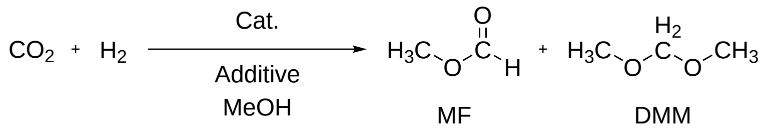

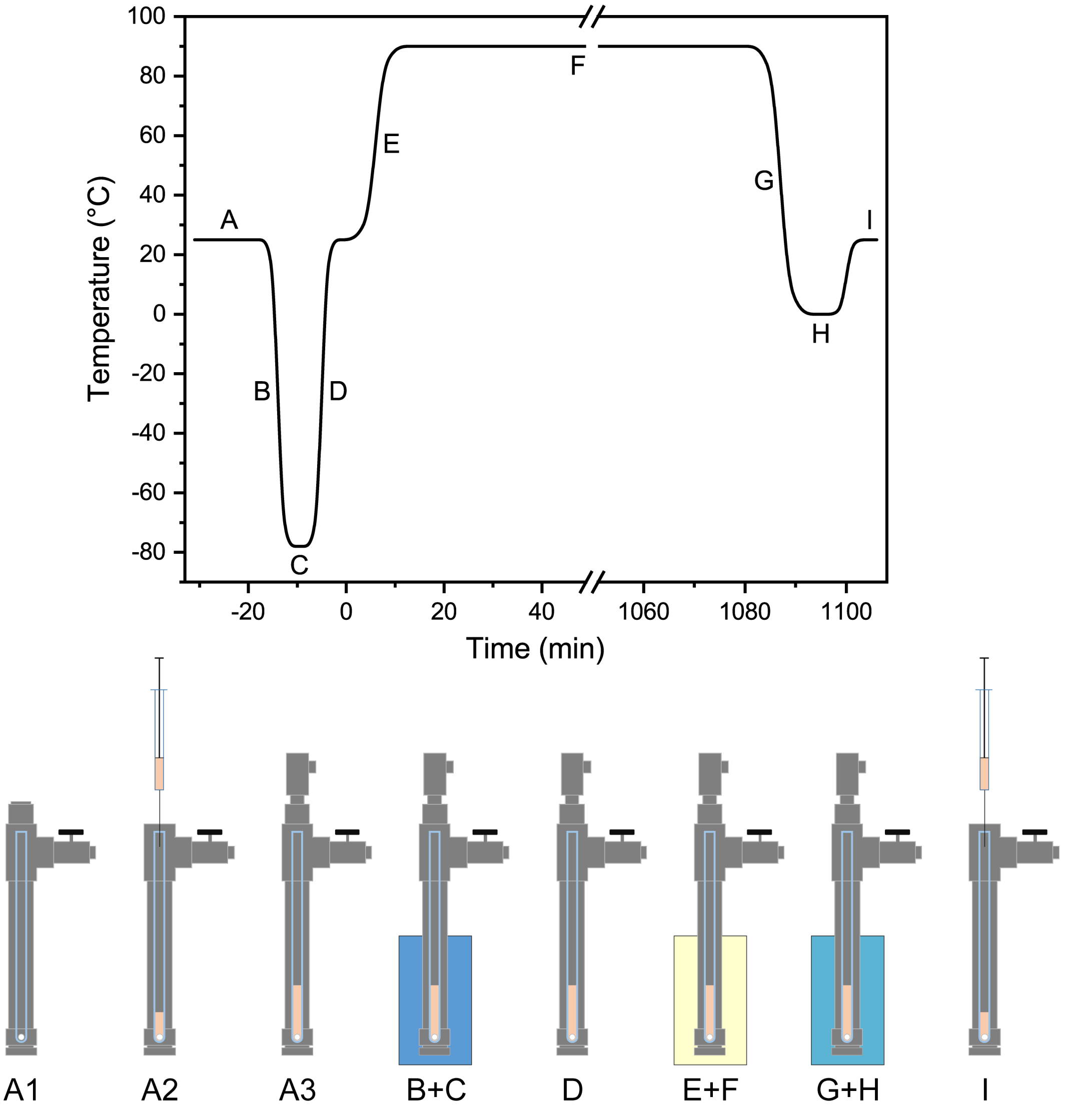

Supplementary Figure 1: Exemplary timeline of an experimental procedure regarding every step and the respective temperature at which it was performed together with a schematic drawing of the experimental setup.

A1: A glass inset is placed in the autoclave and the autoclave is closed. Evacuation of the autoclave and flushing with nitrogen at 25 °C.

A2: Opening of the autoclave and transfer of the reaction mixture into the glass inset in a nitrogen gas counter-flow at 25 °C.

A3: Installing of the proportional relief valve in a nitrogen gas counter-flow at 25 °C. Afterwards, the autoclave is pressurized with CO_2.

B: Cooling of the autoclave to -78 °C using an acetone / dry-ice bath.

C: The autoclave is kept at -78 °C for 5 min in order to deposit CO_2. Afterwards, the autoclave is pressurized with H_2.

D: Warming of the autoclave to 25 °C for 10 min.

E: Heating of the autoclave to the respective temperature (90 °C in the example) using a preheated oil bath. The temperature of the oil bath is stable within ± 0.1 °C after the autoclave is tempered at the desired temperature.

F: The autoclave is kept at constant temperature over the course of the reaction (18 h in the example).

G: Cooling of the autoclave to 0 °C using an ice bath.

H: The autoclave is kept at 0 °C for 15 min.

I: The autoclave is vented carefully and a sample is taken for quantification at 25 °C.

The TONs for DMM and MF were calculated using equations 5.2 and 5.4 and the peaks at 4.57 - 4.58 and 3.73 - 3.77 ppm, respectively.

$$TON_{DMM} = \frac{\frac{V_{cat}}{V_{sample}} \cdot \frac{Int_{DMM}}{x_{Proton,DMM}} \cdot \frac{x_{Proton,mes}}{Int_{mes}} \cdot \frac{\rho_{mes} \cdot V_{mes}}{M_{mes}}}{n_{cat}} \tag{5.1}$$

$$TON_{DMM} = 2.5160\,\frac{\mu mol}{\mu L} \cdot \frac{V_{cat} \cdot Int_{DMM}}{n_{cat}} \tag{5.2}$$

$$TON_{MF} = \frac{\frac{V_{cat}}{V_{sample}} \cdot \frac{Int_{MF}}{x_{Proton,MF}} \cdot \frac{x_{Proton,mes}}{Int_{mes}} \cdot \frac{\rho_{mes} \cdot V_{mes}}{M_{mes}}}{n_{cat}} \tag{5.3}$$

$$TON_{MF} = 1.6773\,\frac{\mu mol}{\mu L} \cdot \frac{V_{cat} \cdot Int_{MF}}{n_{cat}} \tag{5.4}$$

where:

TON_{DMM} = Turnover number for DMM
TON_{MF} = Turnover number for MF
V_{cat} = Volume of catalysis solution
V_{sample} = Volume of sample taken for NMR analysis
Int_{DMM} = Integral of the methylene group of DMM
Int_{MF} = Integral of the methyl group of MF
$x_{Proton,DMM}$ = Number of integrated protons of DMM (here: 2)
$x_{Proton,MF}$ = Number of integrated protons of MF (here: 3)
Int_{mes} = Integral of the aromatic protons of mesitylene (set manually to 3)
$x_{Proton,mes}$ = Number of integrated protons of mesitylene (here: 3)
ρ_{mes} = Density of mesitylene at 25 °C (0.864 g mL^{-1})
V_{mes} = Volume of mesitylene used as internal standard (here: 35 µL)
M_{mes} = Molar mass of mesitylene (120.19 g mol^{-1})
n_{cat} = Amount of catalyst in catalysis solution

The sample standard deviation mentioned for each mean value was calculated using equation 5.5.

$$SD = \sqrt{\frac{\sum_{i=1}^{N}(x_i - \overline{x})^2}{N-1}} \tag{5.5}$$

where:

SD = Standard Deviation
i = Number of experiment
N = Total number of observations in the sample
x_i = Observed values of the sample item
$\overline{x}$ = Mean value of the observations

The average TONs for DMM and MF as well as the ratios and their average values in Chapter 5 were calculated using the exact values obtained by the given integrals of DMM and MF and equations 5.2 and 5.4, respectively. Therefore, these values might vary slightly compared to the average of the rounded values. The sample standard deviations were calculated using the exact values of the TONs and the ratios.

The respective values for the amount of DMM and MF formed in the reactions without any catalyst were calculated using the given integrals of DMM and MF and equations 5.2 and 5.4, respectively, except that only the counter was calculated and the denominator was neglected. The average amounts of DMM and MF as well as the ratios and their average values were calculated using the exact values.

5.2 References

NMR Spectra of DMM[17]

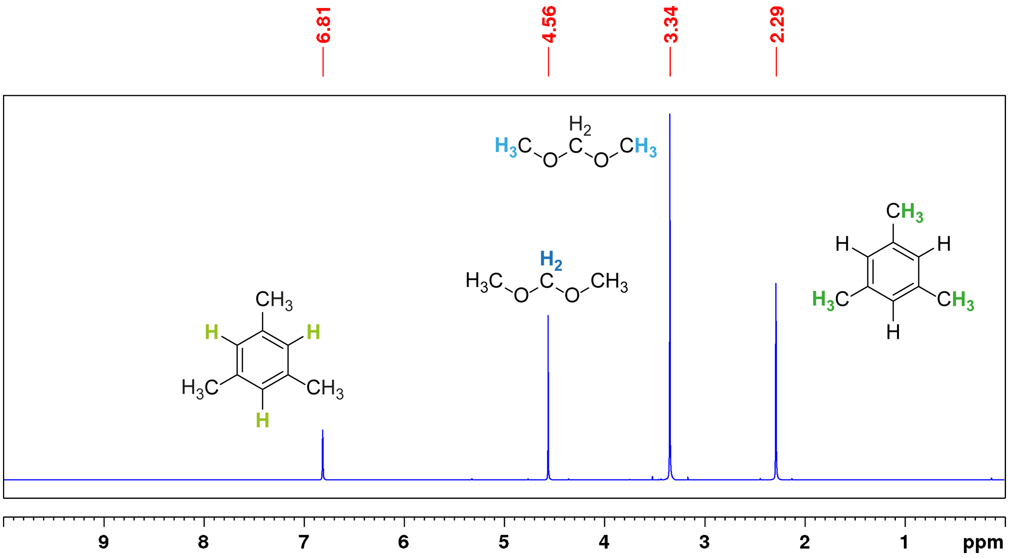

Supplementary Figure 2: NMR spectra of 50 µL DMM and 35 µL mesitylene in 450 µL DCM-d_2.

NMR Spectra of MF[17]

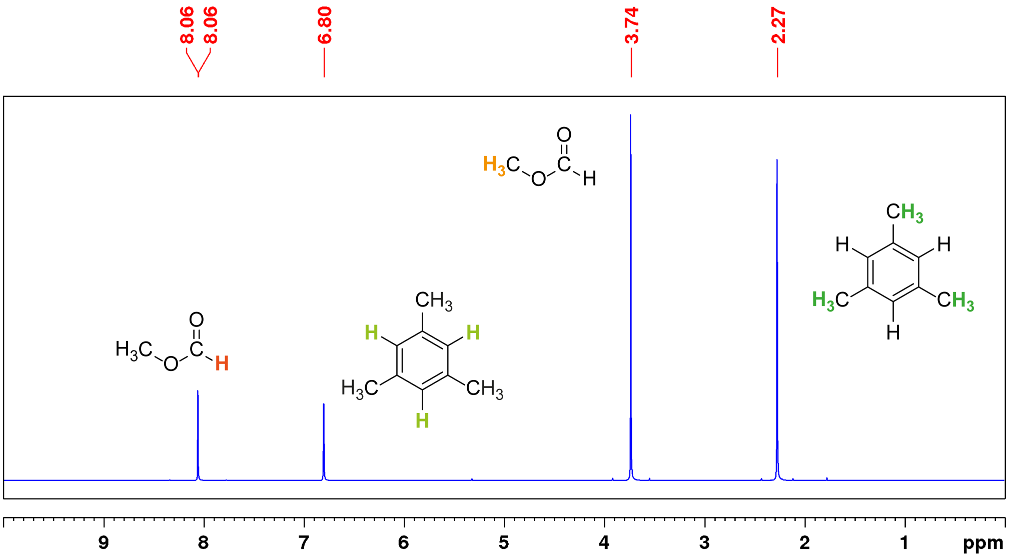

Supplementary Figure 3: NMR spectra of 50 µL MF and 35 µL mesitylene in 450 µL DCM-d_2.

NMR Spectra of Mesitylene[17]

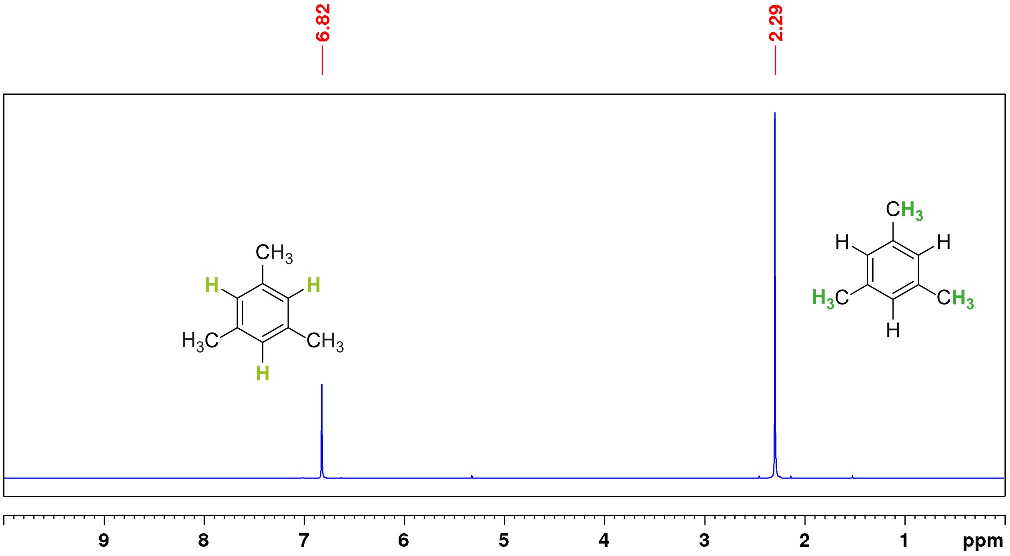

Supplementary Figure 4: NMR spectra of 35 µL mesitylene in 450 µL DCM-d_2.

5.3 First DoE

5.3.1 First Triple

$$T = 90\,°C \qquad n_{cat} = 0.75\ \mu mol$$
$$p_{H2} = 90\,bar \qquad n_{add} = 0.78125\ \mu mol$$
$$p_{CO2} = 20\,bar \qquad add = Al(OTf)_3$$
$$t = 18\,h \qquad V_{MeOH} = 0.25\ mL$$

Supplementary Table 2: Catalysis results of the first triple.

	$Integral_{DMM}$	TON_{DMM}	$Integral_{MF}$	TON_{MF}	Ratio
Experiment 1	0.2323	195	0.1594	89	2.19
Experiment 2	0.2337	196	0.1523	85	2.30
Experiment 3	0.2629	220	0.1691	95	2.33
Average	-	204(14)	-	90(5)	2.27(0.08)

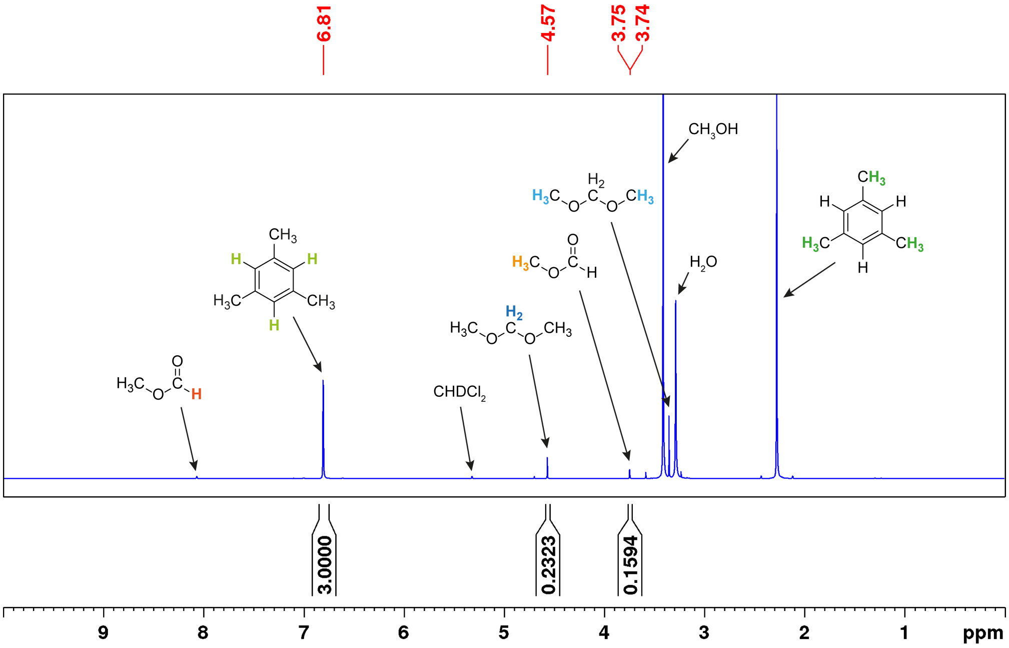

Supplementary Figure 5: Experiment 1.

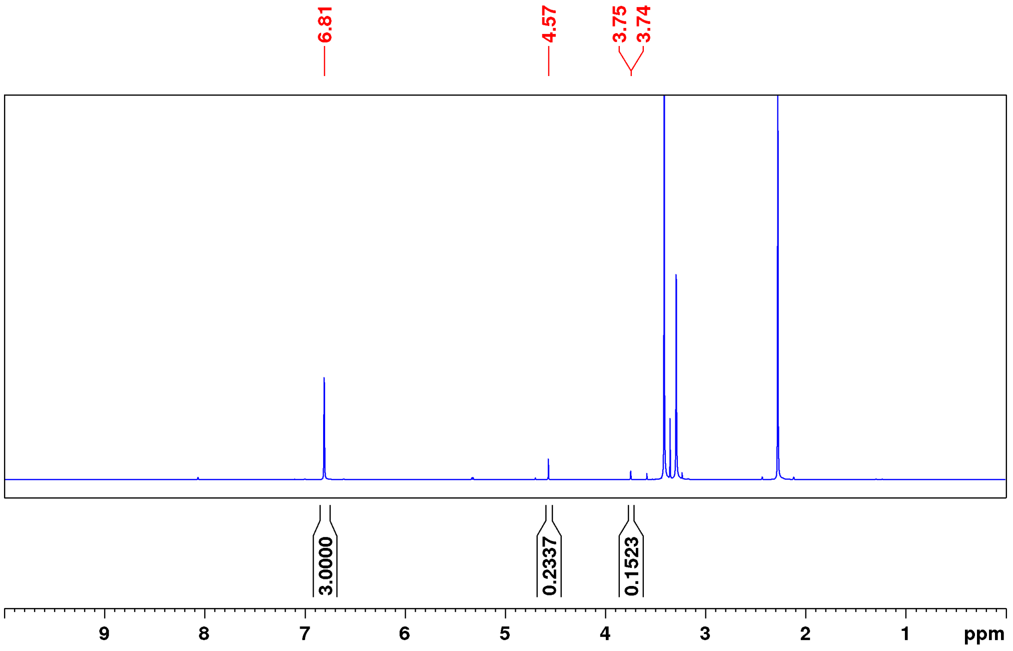

Supplementary Figure 6: Experiment 2.

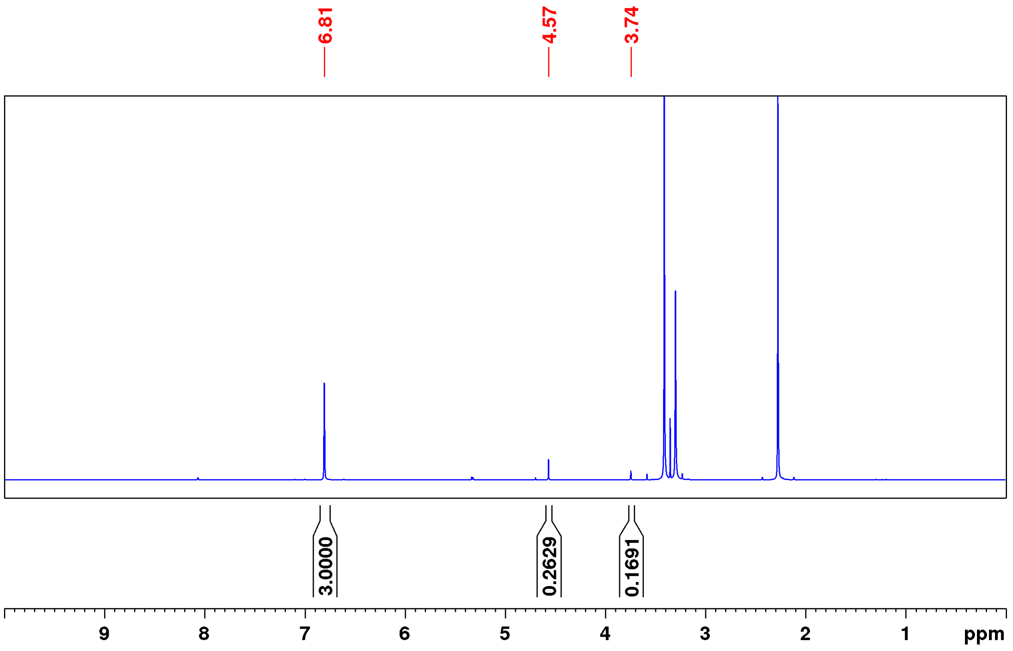

Supplementary Figure 7: Experiment 3.

5.3.2 Second Triple

$$T = 90\,°\mathrm{C} \qquad n_{cat} = 0.75\ \mu\mathrm{mol}$$
$$p_{H2} = 90\,\mathrm{bar} \qquad n_{add} = 0.78125\ \mu\mathrm{mol}$$
$$p_{CO2} = 20\,\mathrm{bar} \qquad \mathrm{add} = \mathrm{Al(OTf)_3}$$
$$t = 18\,\mathrm{h} \qquad V_{MeOH} = 0.5\ \mathrm{mL}$$

Supplementary Table 3: Catalysis results of the second triple.

	$Integral_{DMM}$	TON_{DMM}	$Integral_{MF}$	TON_{MF}	Ratio
Experiment 1	0.0738	124	0.1882	210	0.59
Experiment 2	0.0678	114	0.1710	191	0.59
Experiment 3	0.0720	121	0.1898	212	0.57
Average	-	119(5)	-	205(12)	0.58(0.01)

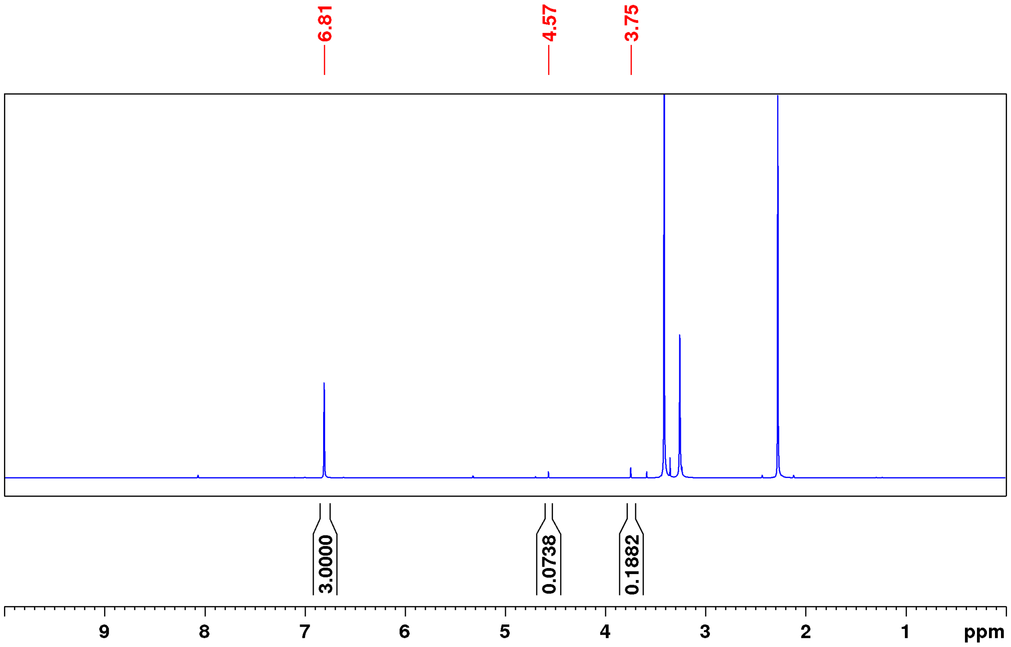

Supplementary Figure 8: Experiment 1.

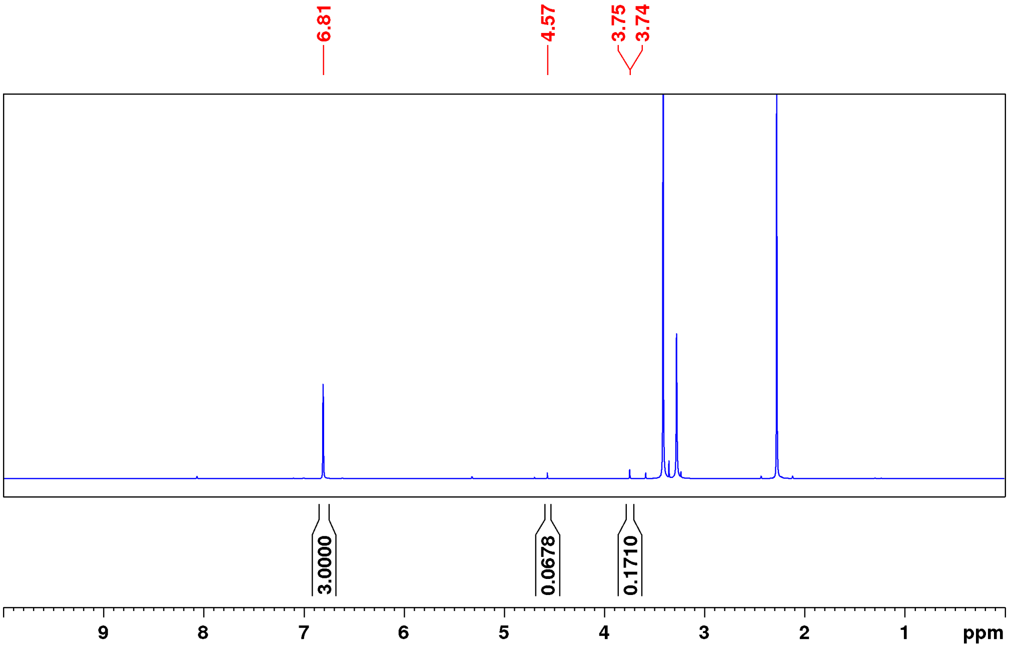

Supplementary Figure 9: Experiment 2.

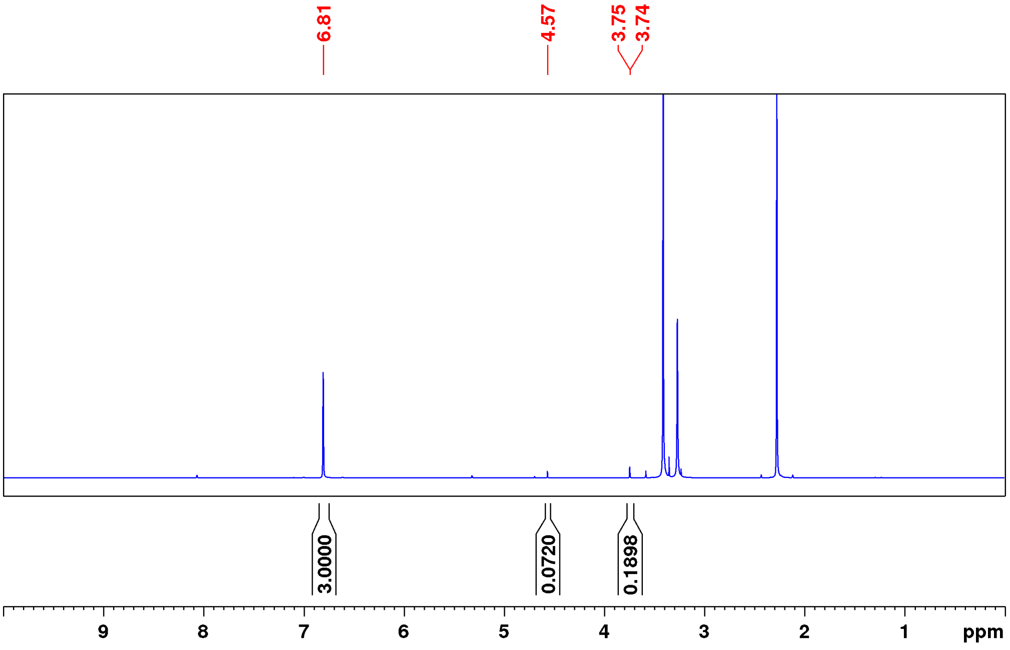

Supplementary Figure 10: Experiment 3.

5.3.3 Third Triple

$$T = 90\,°C \qquad n_{cat} = 0.75\ \mu mol$$
$$p_{H2} = 90\,bar \qquad n_{add} = 1.953125\ \mu mol$$
$$p_{CO2} = 20\,bar \qquad add = Al(OTf)_3$$
$$t = 18\,h \qquad V_{MeOH} = 0.375\ mL$$

Supplementary Table 4: Catalysis results of the third triple.

	$Integral_{DMM}$	TON_{DMM}	$Integral_{MF}$	TON_{MF}	Ratio
Experiment 1	0.2678	337	0.1458	122	2.76
Experiment 2	0.2665	335	0.1309	110	3.05
Experiment 3	0.2820	355	0.1379	116	3.07
Average	-	342(11)	-	116(6)	2.96(0.18)

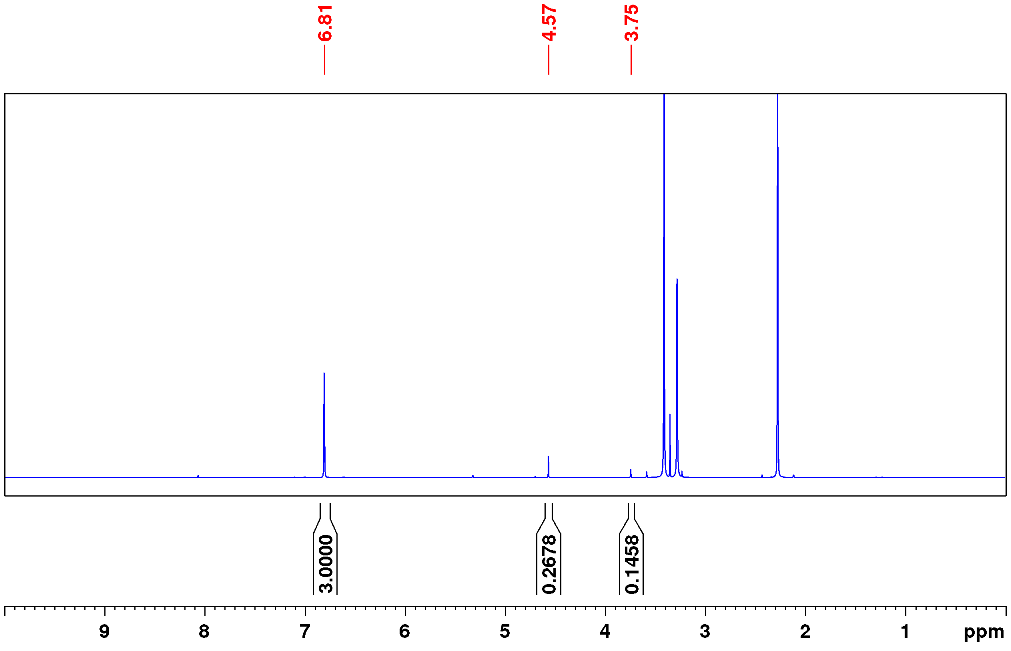

Supplementary Figure 11: Experiment 1.

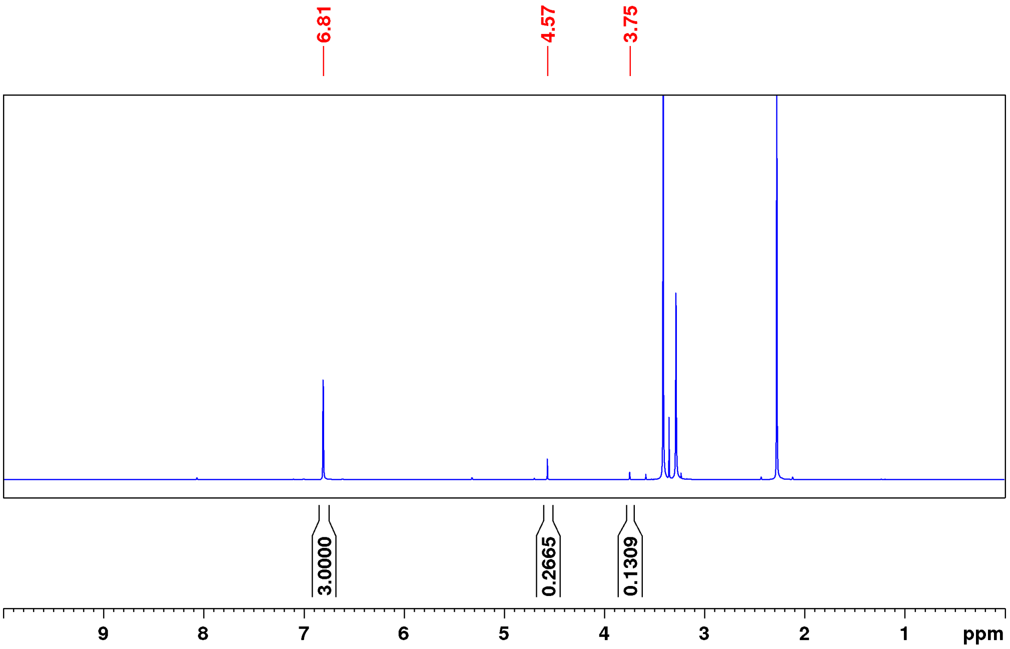

Supplementary Figure 12: Experiment 2.

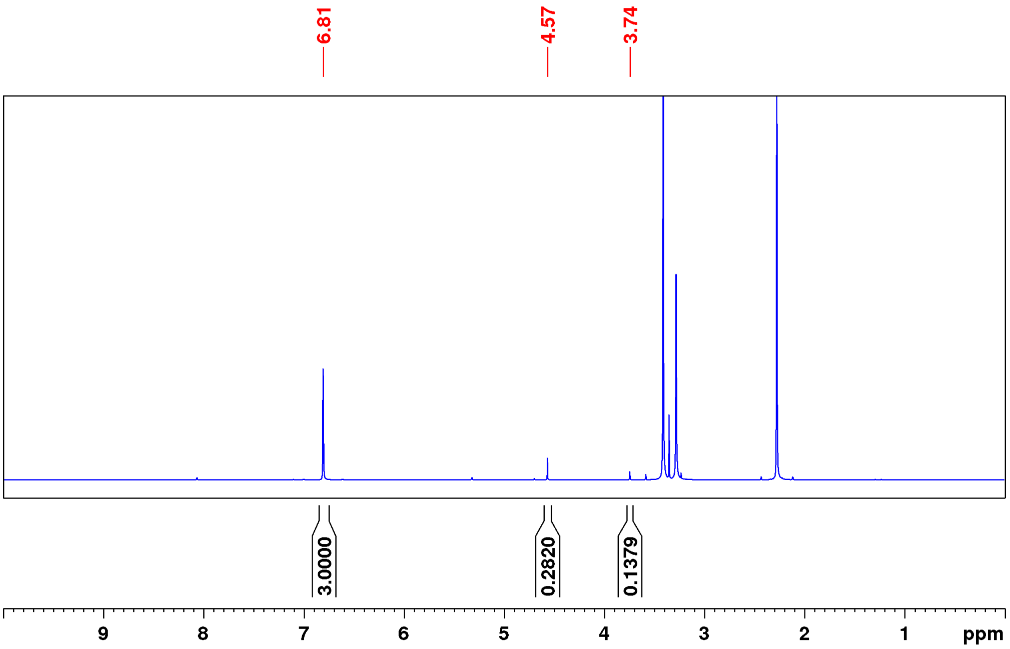

Supplementary Figure 13: Experiment 3.

5.3.4 Fourth Triple

$$T = 90\,°C \qquad n_{cat} = 0.1875\ \mu mol$$
$$p_{H2} = 90\,bar \qquad n_{add} = 3.125\ \mu mol$$
$$p_{CO2} = 20\,bar \qquad add = Al(OTf)_3$$
$$t = 18\,h \qquad V_{MeOH} = 0.25\ mL$$

Supplementary Table 5: Catalysis results of the fourth triple.

	$Integral_{DMM}$	TON_{DMM}	$Integral_{MF}$	TON_{MF}	Ratio
Experiment 1	0.3464	1162	0.1955	437	2.66
Experiment 2	0.3419	1147	0.1857	415	2.76
Experiment 3	0.3101	1040	0.2047	458	2.27
Average	-	1116(66)	-	437(21)	2.56(0.26)

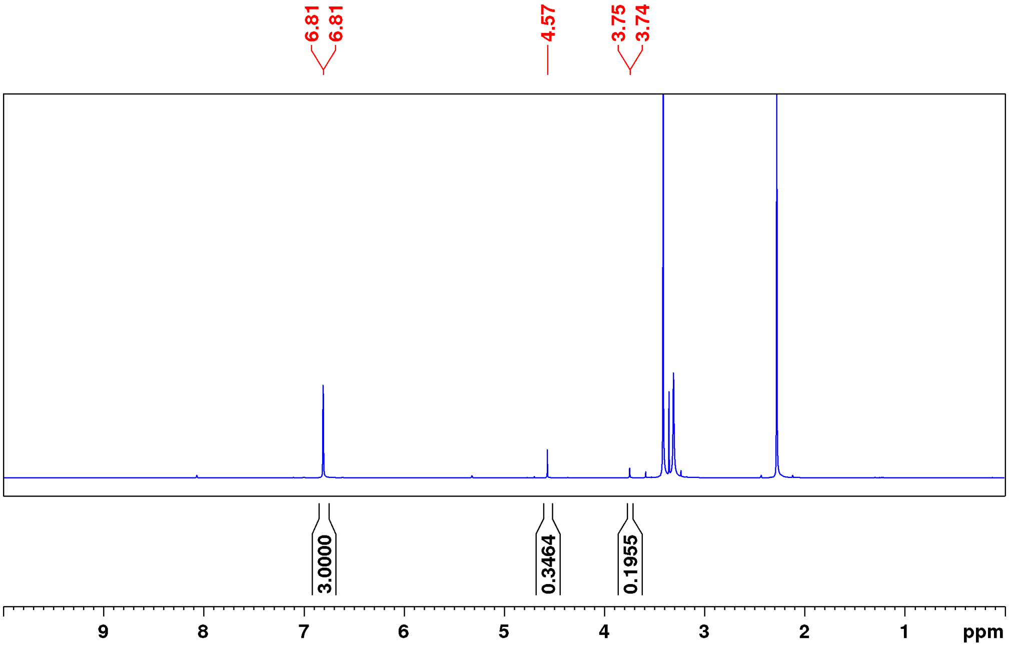

Supplementary Figure 14: Experiment 1.

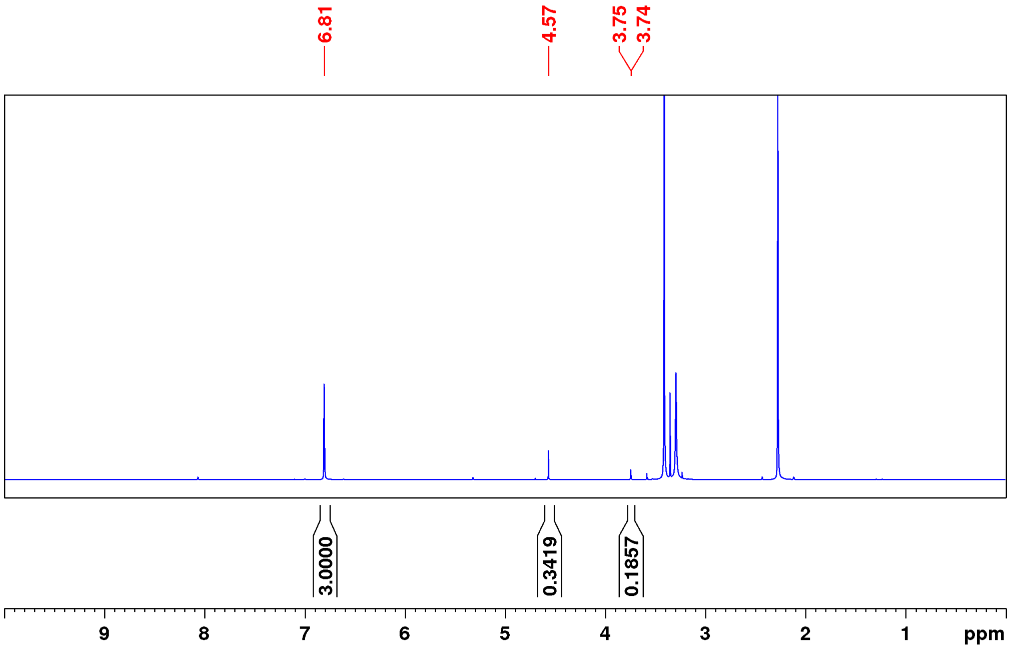

Supplementary Figure 15: Experiment 2.

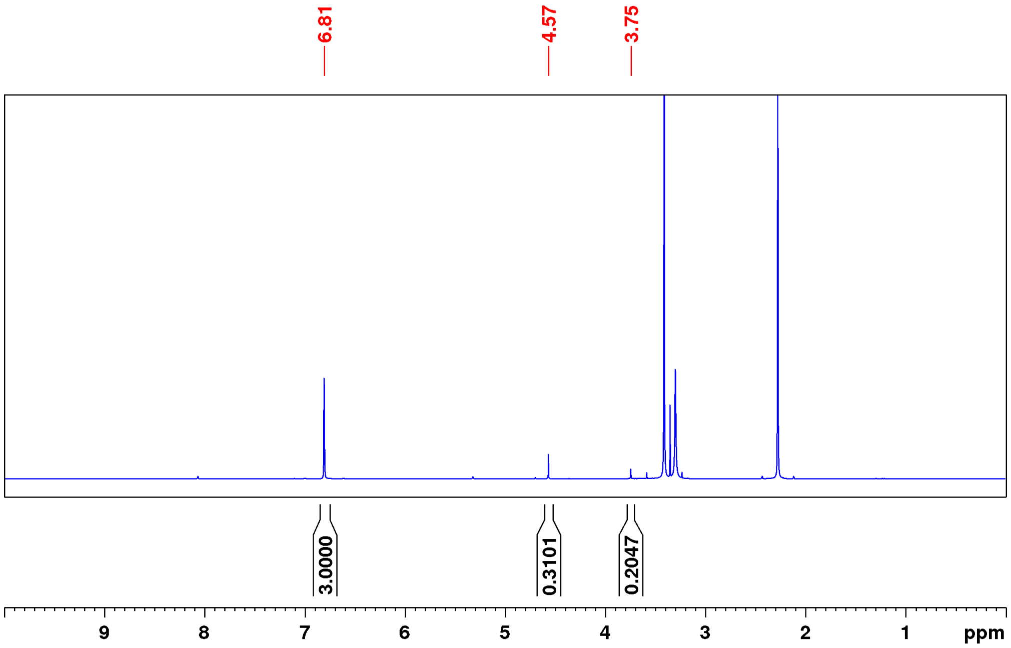

Supplementary Figure 16: Experiment 3.

5.3.5 Fifth Triple

$$T = 90\,°C \qquad n_{cat} = 0.75\ \mu mol$$
$$p_{H2} = 90\,bar \qquad n_{add} = 3.125\ \mu mol$$
$$p_{CO2} = 20\,bar \qquad add = Al(OTf)_3$$
$$t = 18\,h \qquad V_{MeOH} = 0.25\ mL$$

Supplementary Table 6: Catalysis results of the fifth triple.

	$Integral_{DMM}$	TON_{DMM}	$Integral_{MF}$	TON_{MF}	Ratio
Experiment 1	0.3701	310	0.1393	78	3.99
Experiment 2	0.3670	308	0.1436	80	3.83
Experiment 3	0.3855	323	0.1434	80	4.03
Average	-	314(8)	-	79(1)	3.95(0.10)

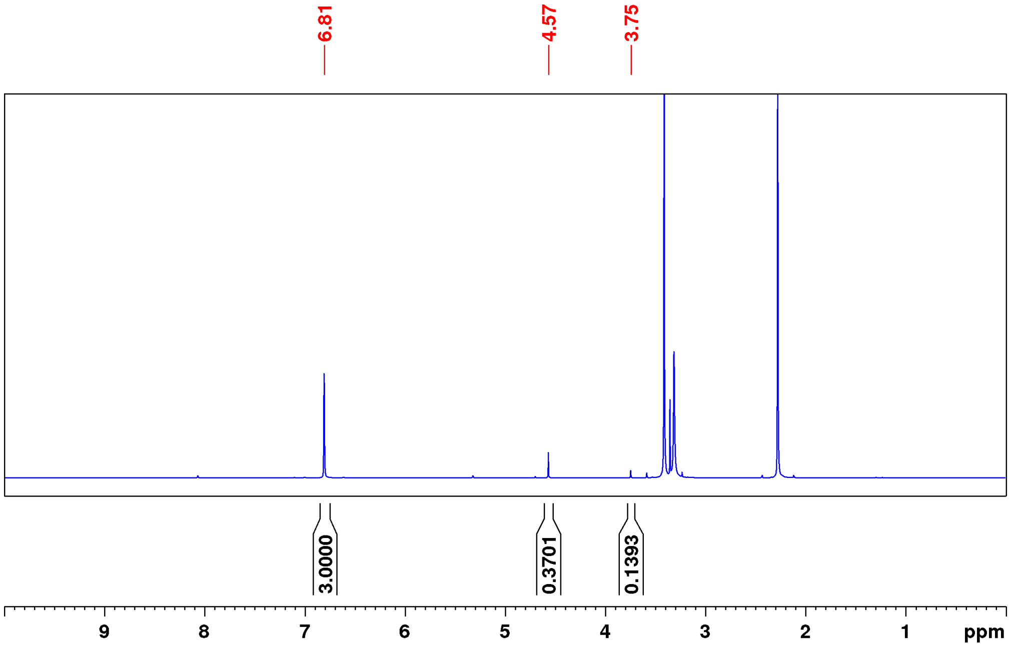

Supplementary Figure 17: Experiment 1.

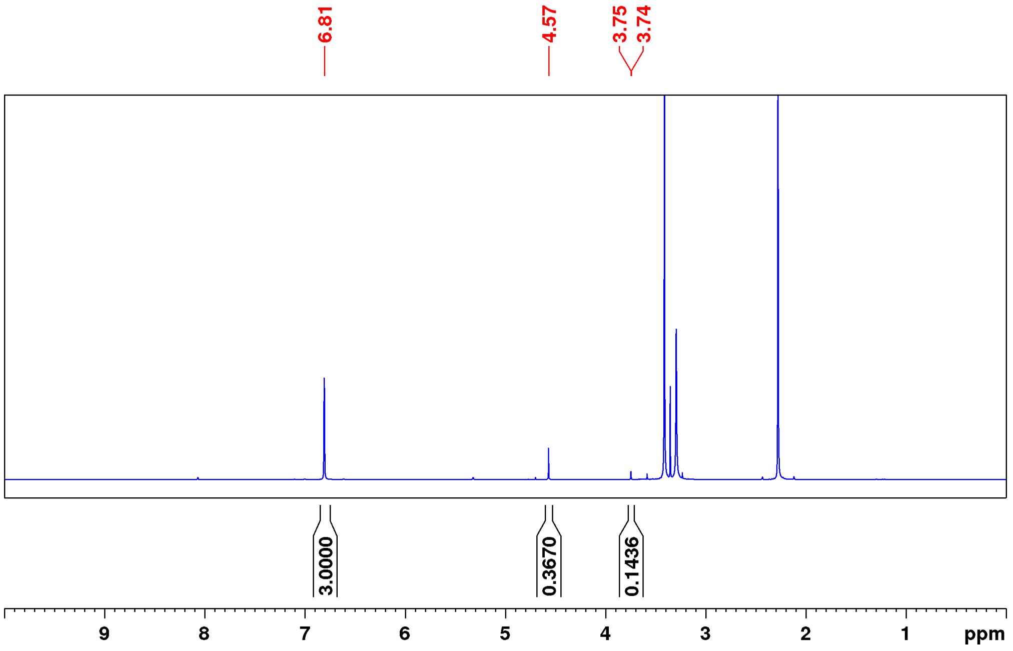

Supplementary Figure 18: Experiment 2.

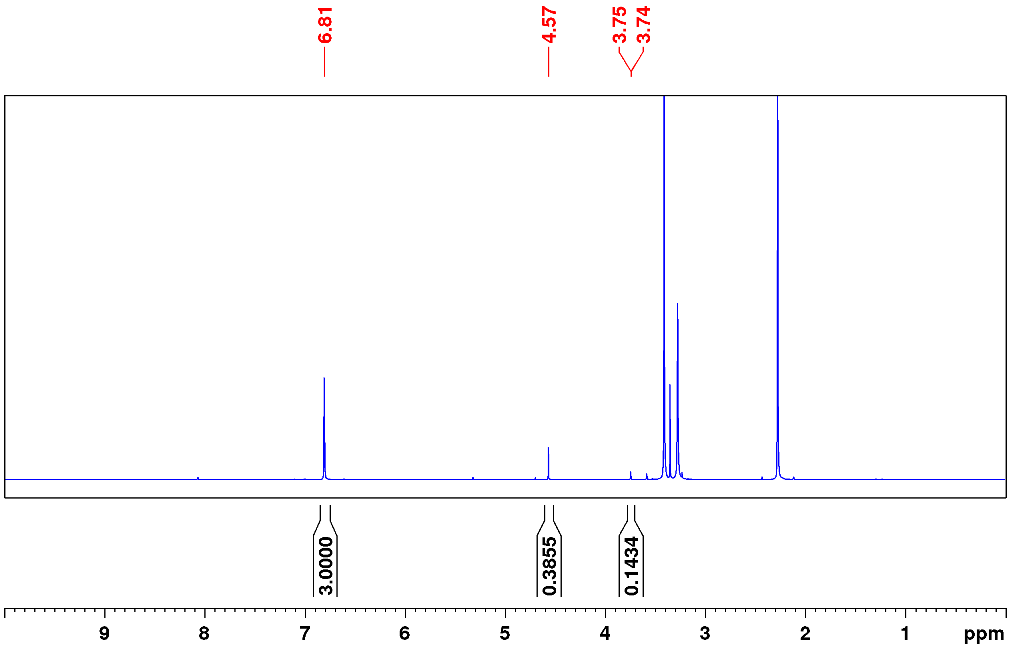

Supplementary Figure 19: Experiment 3.

5.3.6 Sixth Triple

$T = 90\,°C$	$n_{cat} = 0.46875$ µmol
$p_{H2} = 90$ bar	$n_{add} = 3.125$ µmol
$p_{CO2} = 20$ bar	add = $Al(OTf)_3$
$t = 18$ h	$V_{MeOH} = 0.375$ mL

Supplementary Table 7: Catalysis results of the sixth triple.

	$Integral_{DMM}$	TON_{DMM}	$Integral_{MF}$	TON_{MF}	Ratio
Experiment 1	0.3119	628	0.1448	194	3.23
Experiment 2	0.3343	673	0.1588	213	3.16
Experiment 3	0.2733	550	0.1457	195	2.81
Average	-	617(62)	-	201(11)	3.07(0.22)

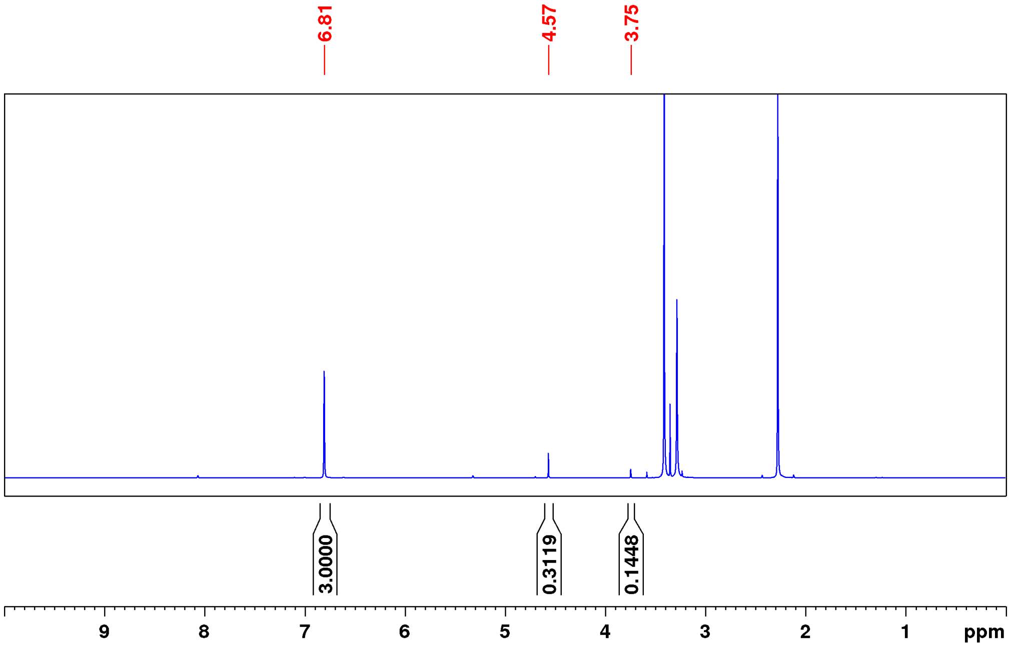

Supplementary Figure 20: Experiment 1.

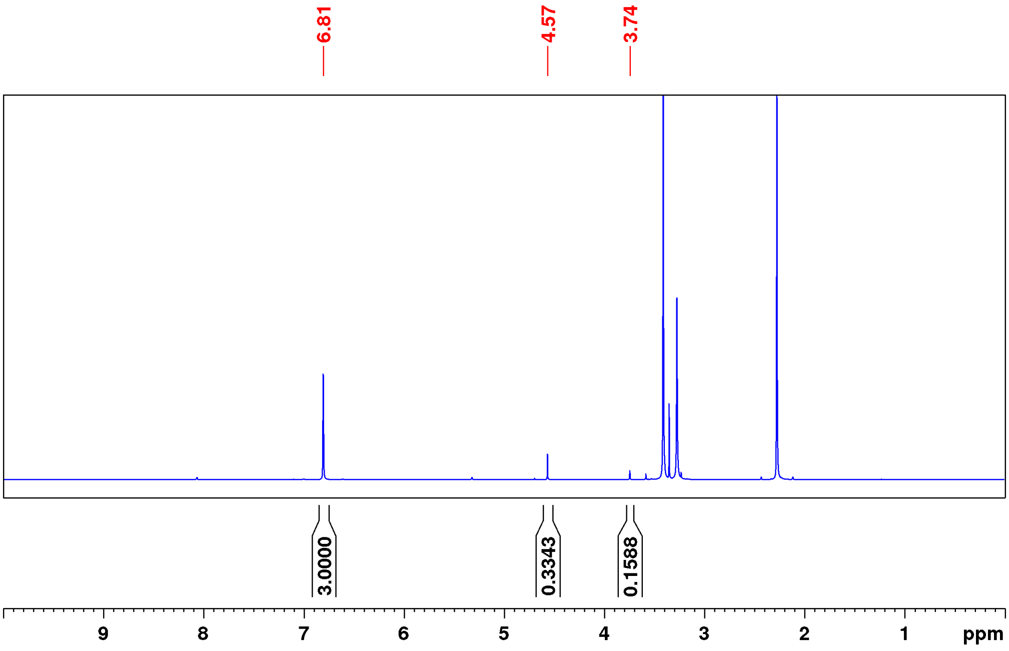

Supplementary Figure 21: Experiment 2.

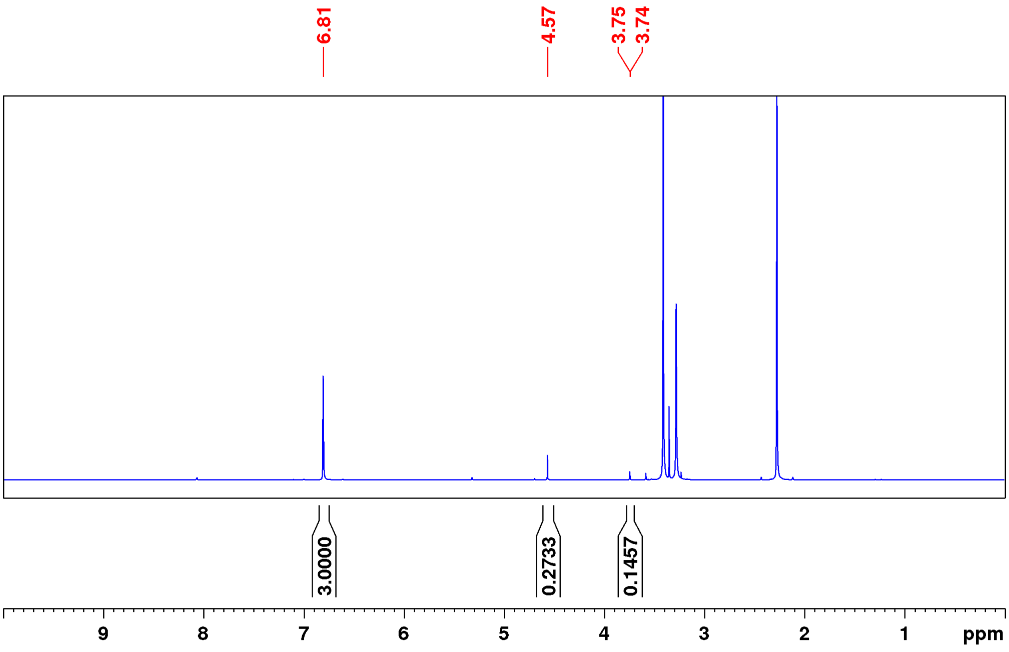

Supplementary Figure 22: Experiment 3.

5.4 First Relaxation

5.4.1 10 % Relaxation

$T = 90\,°C$	$n_{cat} = 0.1313\ \mu mol$
$p_{H2} = 90\,bar$	$n_{add} = 3.3594\ \mu mol$
$p_{CO2} = 20\,bar$	add = $Al(OTf)_3$
$t = 18\,h$	$V_{MeOH} = 0.525\ mL$

Supplementary Table 8: Catalysis results of the 10 % relaxation trials.

	$Integral_{DMM}$	TON_{DMM}	$Integral_{MF}$	TON_{MF}	Ratio
Experiment 1	0.1466	1475	0.2543	1706	0.86
Experiment 2	0.1065	1071	0.2133	1431	0.75
Experiment 3	0.1565	1574	0.2300	1543	1.02
Average	-	1374(266)	-	1560(138)	0.88(0.14)

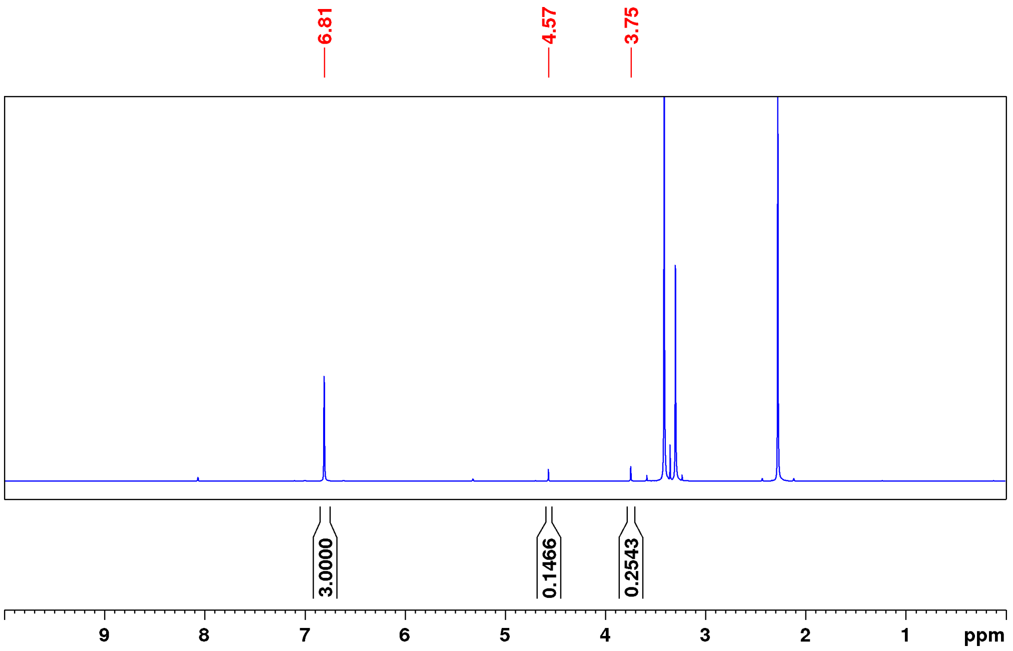

Supplementary Figure 23: Experiment 1.

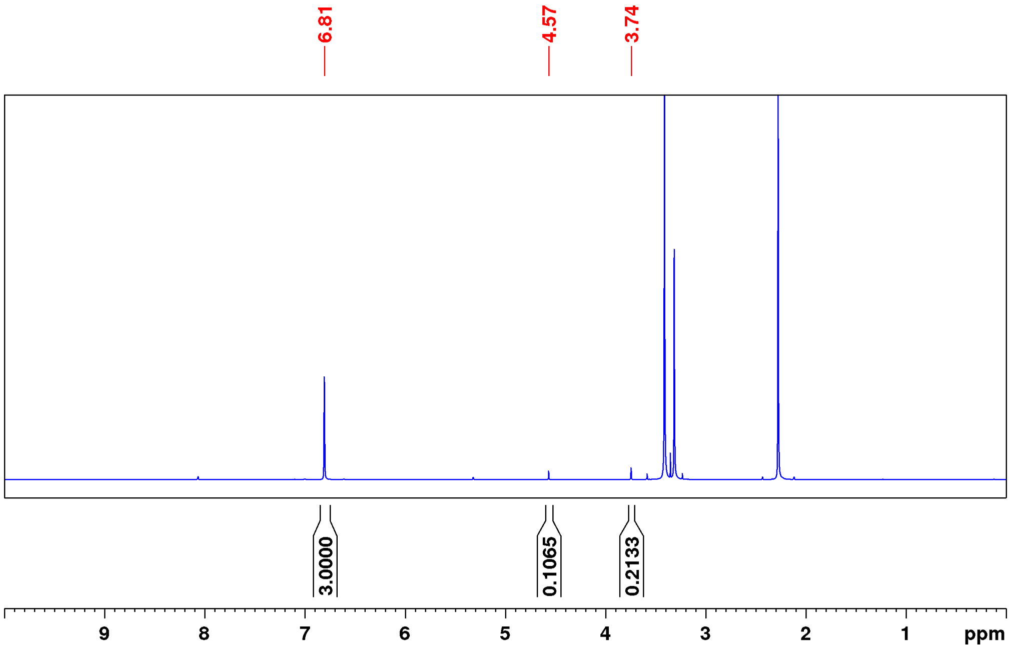

Supplementary Figure 24: Experiment 2.

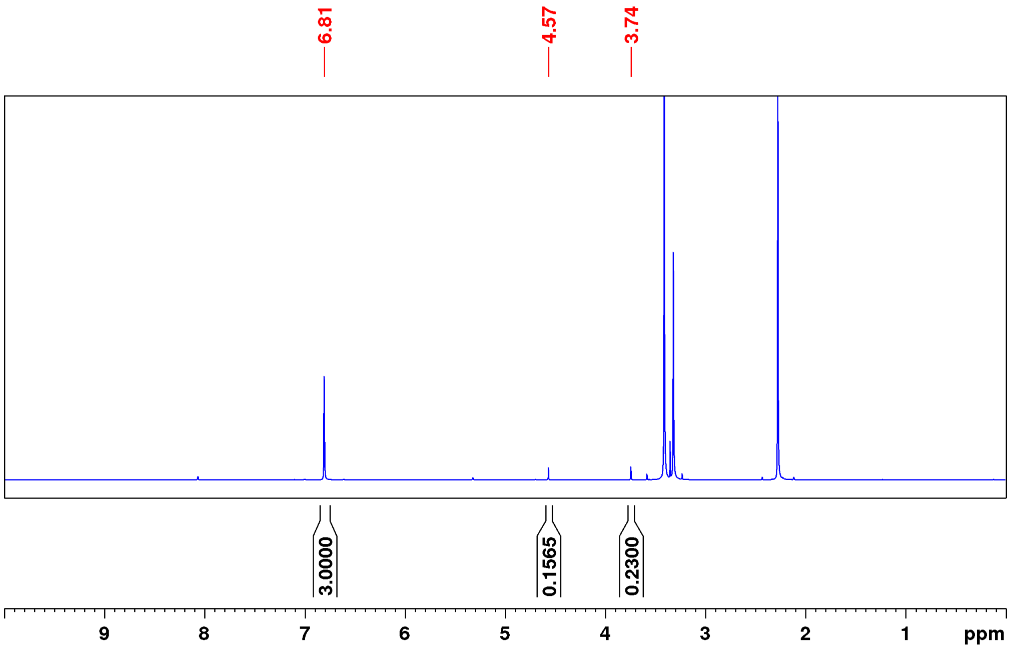

Supplementary Figure 25: Experiment 3.

5.4.2 20 % Relaxation

$$T = 90\,°C \qquad n_{cat} = 0.075\ \mu mol$$
$$p_{H2} = 90\,bar \qquad n_{add} = 3.576\ \mu mol$$
$$p_{CO2} = 20\,bar \qquad add = Al(OTf)_3$$
$$t = 18\,h \qquad V_{MeOH} = 0.55\ mL$$

Supplementary Table 9: Catalysis results of the 20 % relaxation trials.

	$Integral_{DMM}$	TON_{DMM}	$Integral_{MF}$	TON_{MF}	Ratio
Experiment 1	0.0838	1546	0.2273	2796	0.55
Experiment 2	0.0822	1517	0.2332	2868	0.53
Experiment 3	0.0575	1061	0.2131	2621	0.40
Average	-	1375(272)	-	2762(127)	0.50(0.08)

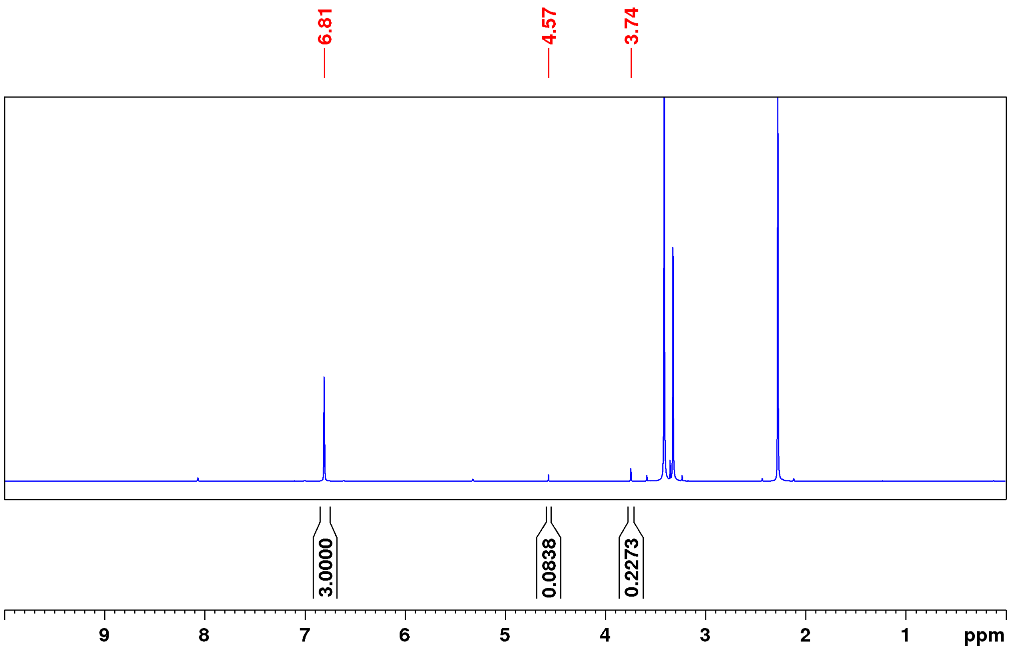

Supplementary Figure 26: Experiment 1.

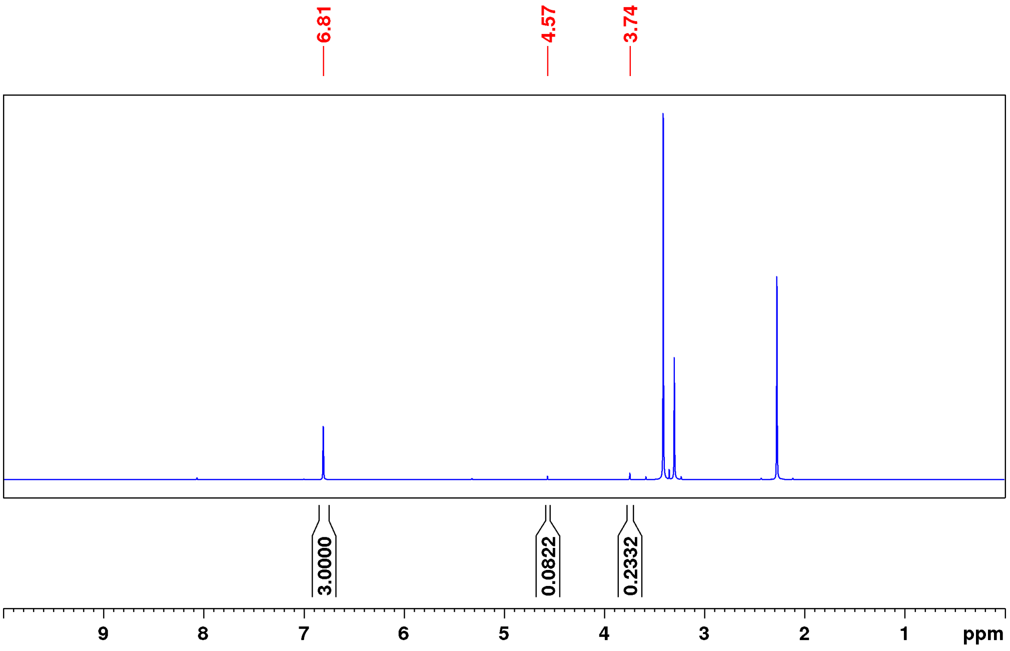

Supplementary Figure 27: Experiment 2.

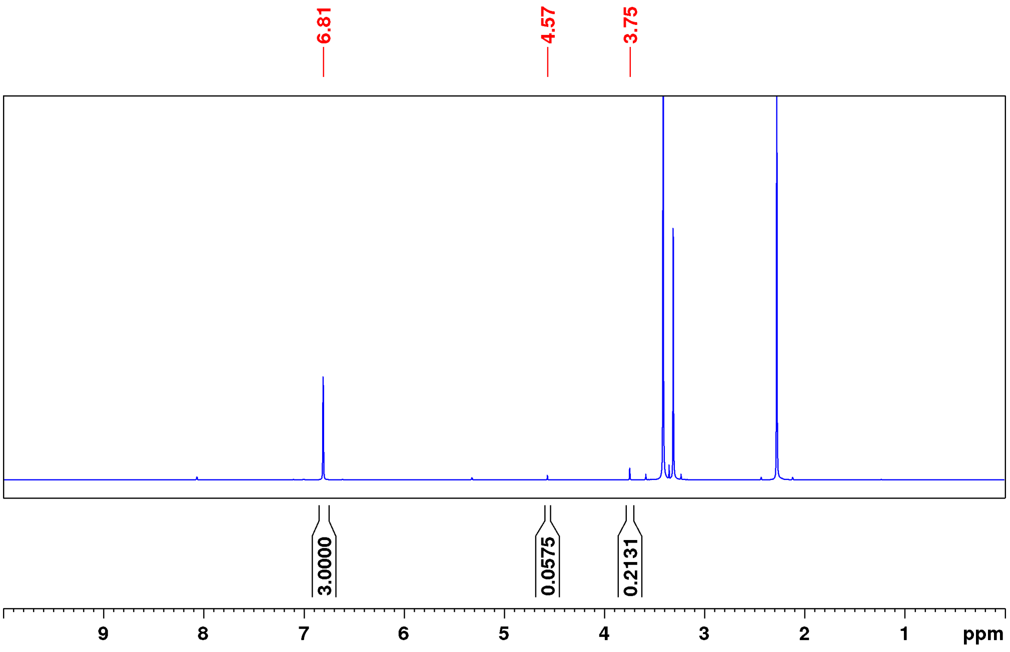

Supplementary Figure 28: Experiment 3.

5.4.3 30 % Relaxation

$$T = 90\,^{\circ}\mathrm{C} \qquad n_{cat} = 0.0188\ \mu\mathrm{mol}$$
$$p_{H2} = 90\,\mathrm{bar} \qquad n_{add} = 3.6594\ \mu\mathrm{mol}$$
$$p_{CO2} = 20\,\mathrm{bar} \qquad \mathrm{add} = \mathrm{Al(OTf)_3}$$
$$t = 18\,\mathrm{h} \qquad V_{MeOH} = 0.575\ \mathrm{mL}$$

Supplementary Table 10: Catalysis results of the 30 % relaxation trials.

	$\mathrm{Integral_{DMM}}$	$\mathrm{TON_{DMM}}$	$\mathrm{Integral_{MF}}$	$\mathrm{TON_{MF}}$	Ratio
Experiment 1	0.0005	38	0.0200	1026	0.04
Experiment 2	0.0001	8	0.0194	995	0.01
Experiment 3	0.0064	492	0.0214	1098	0.45
Average	-	180(271)	-	1040(53)	0.16(0.25)

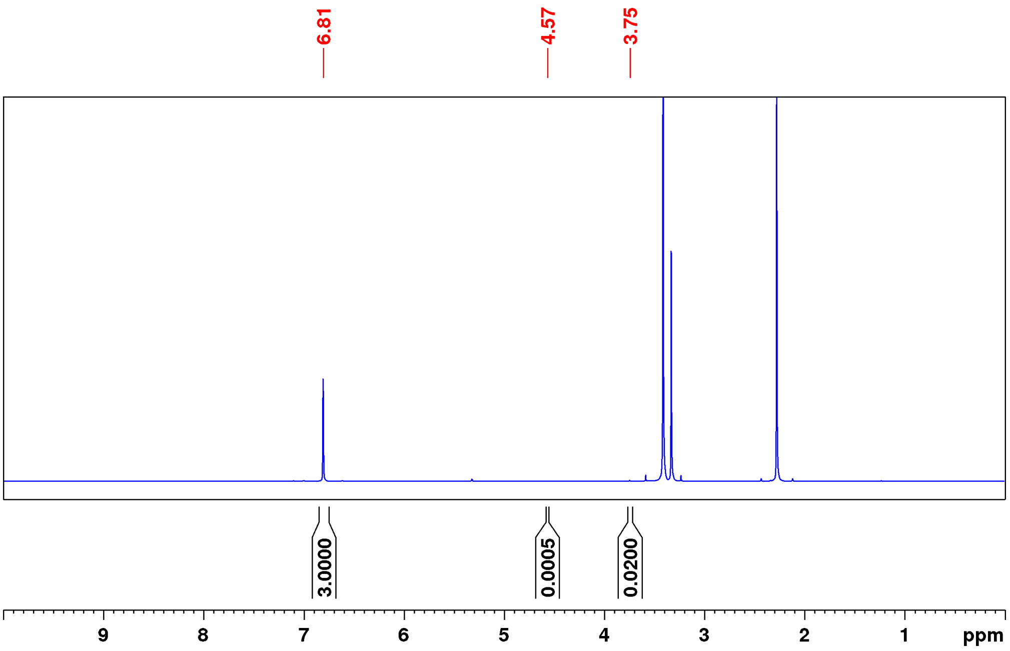

Supplementary Figure 29: Experiment 1.

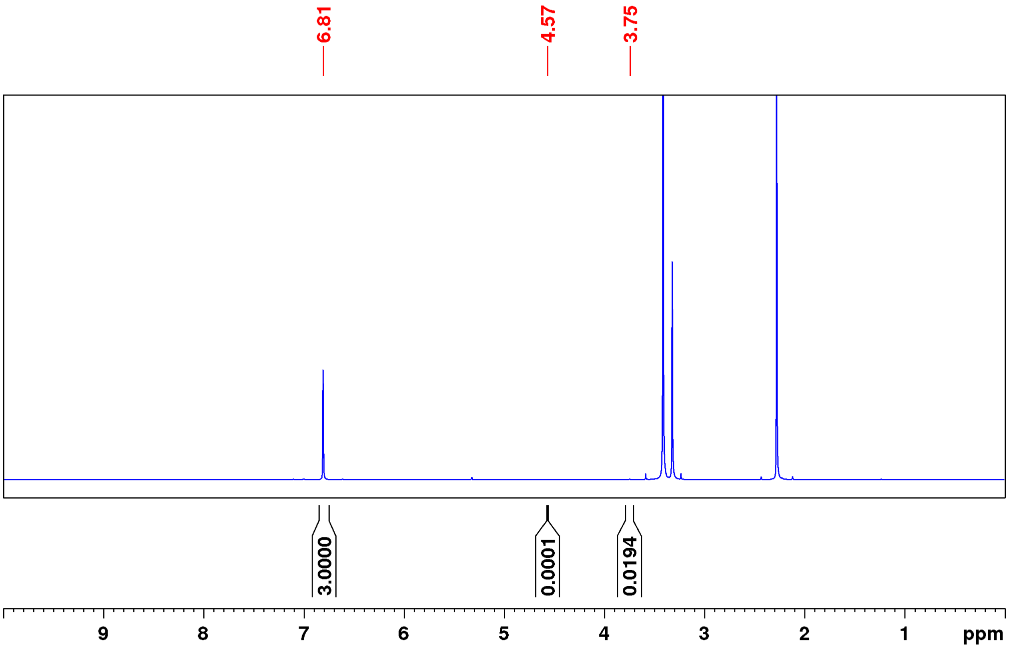

Supplementary Figure 30: Experiment 2.

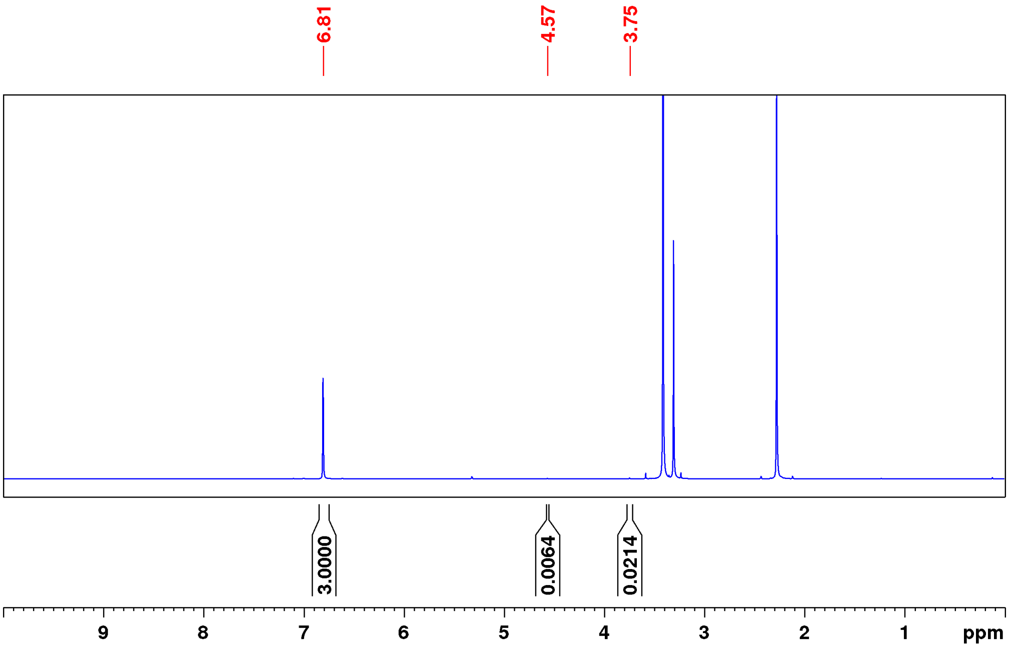

Supplementary Figure 31: Experiment 3.

5.5 Second DoE

5.5.1 Replication: Best Hit First Relaxation

$$T = 90\,°C \qquad n_{cat} = 0.075\ \mu mol$$
$$p_{H2} = 90\,bar \qquad n_{add} = 3.576\ \mu mol$$
$$p_{CO2} = 20\,bar \qquad add = Al(OTf)_3$$
$$t = 18\,h \qquad V_{MeOH} = 0.55\ mL$$

Supplementary Table 11: Catalysis results of the replication study.

	$Integral_{DMM}$	TON_{DMM}	$Integral_{MF}$	TON_{MF}	Ratio
Experiment 1	0.0767	1415	0.2207	2715	0.52
Experiment 2	0.0741	1367	0.2228	2741	0.50
Experiment 3	0.0650	1199	0.2408	2962	0.40
Average	-	1327(113)	-	2806(136)	0.48(0.06)

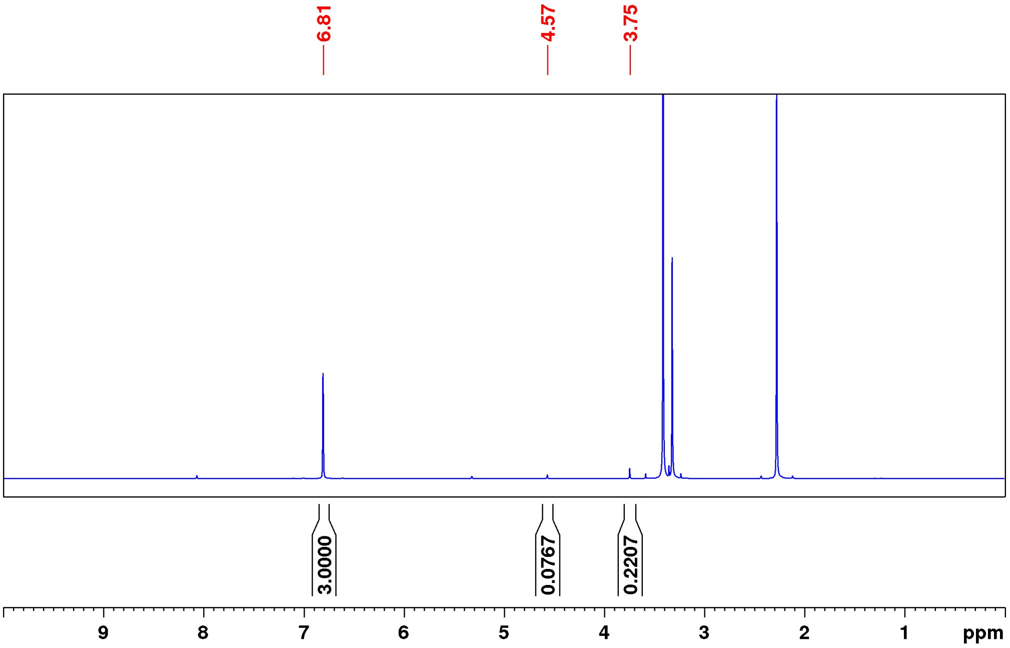

Supplementary Figure 32: Experiment 1.

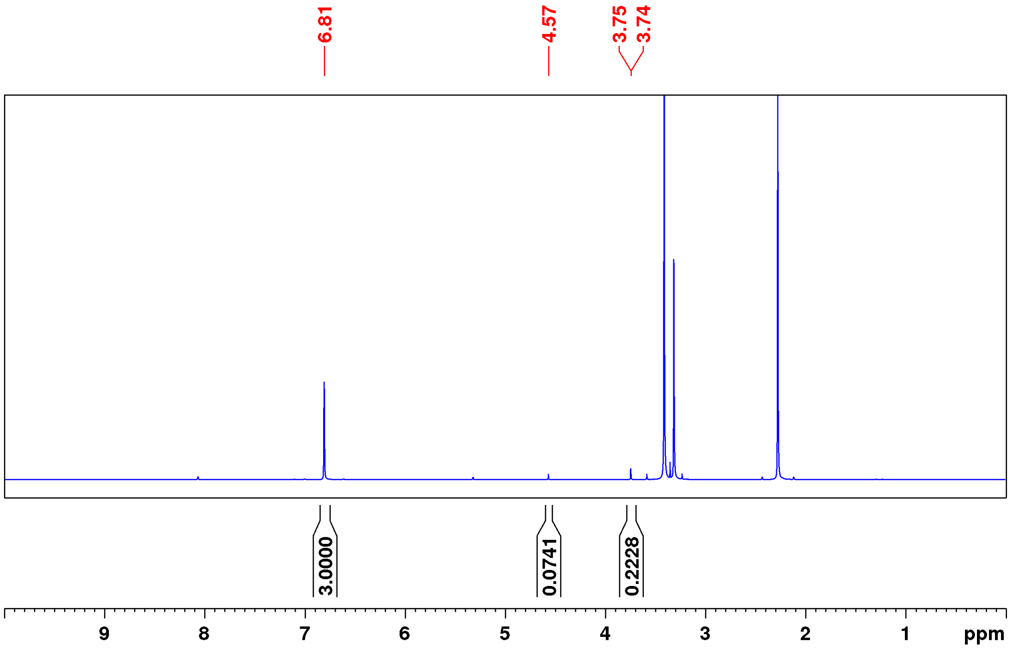

Supplementary Figure 33: Experiment 2.

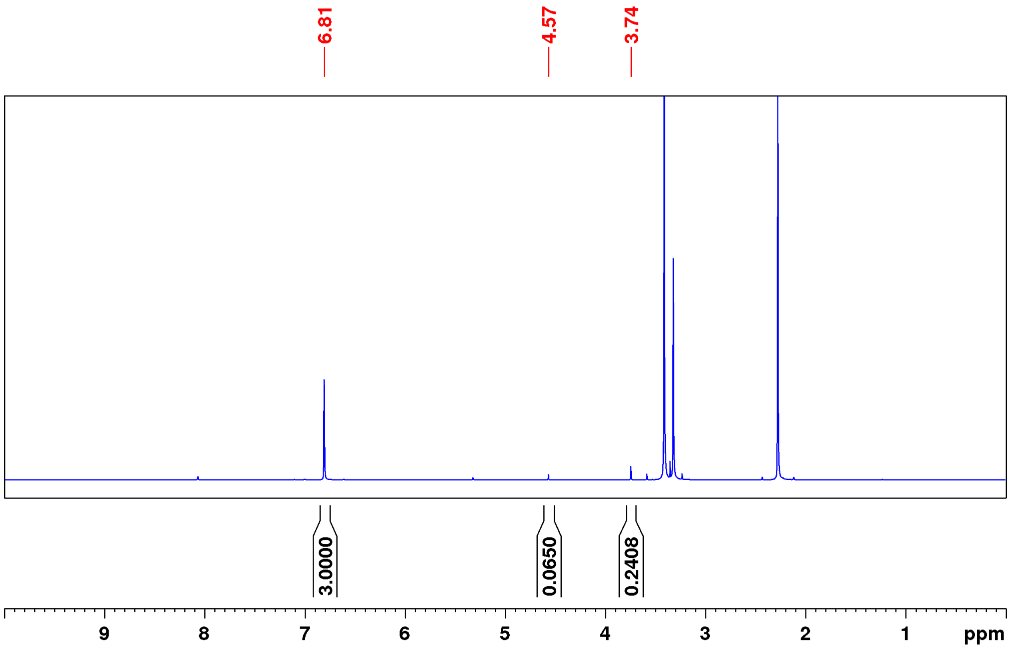

Supplementary Figure 34: Experiment 3.

5.5.2 First Triple

$$T = 80\,°C \qquad n_{cat} = 0.075\ \mu mol$$
$$p_{H2} = 100\,bar \qquad n_{add} = 3.576\ \mu mol$$
$$p_{CO2} = 25\,bar \qquad add = Al(OTf)_3$$
$$t = 20\,h \qquad V_{MeOH} = 0.55\ mL$$

Supplementary Table 12: Catalysis results of the first triple.

	$Integral_{DMM}$	TON_{DMM}	$Integral_{MF}$	TON_{MF}	Ratio
Experiment 1	0.0454	838	0.2593	3190	0.26
Experiment 2	0.0501	924	0.2623	3226	0.29
Experiment 3	0.0491	906	0.2785	3426	0.26
Average	-	889(46)	-	3281(127)	0.27(0.01)

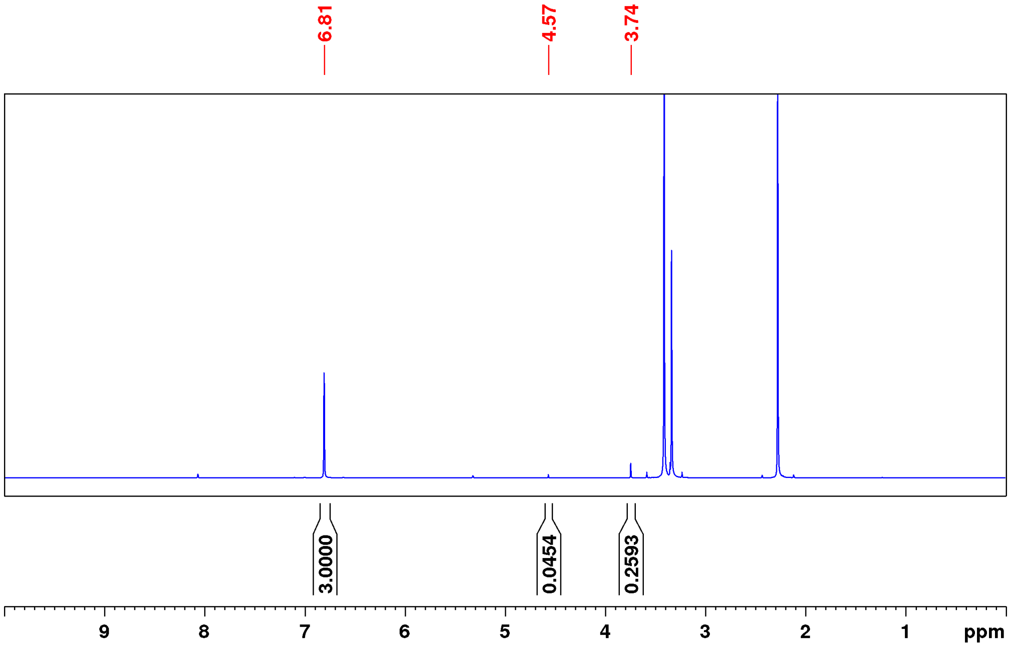

Supplementary Figure 35: Experiment 1.

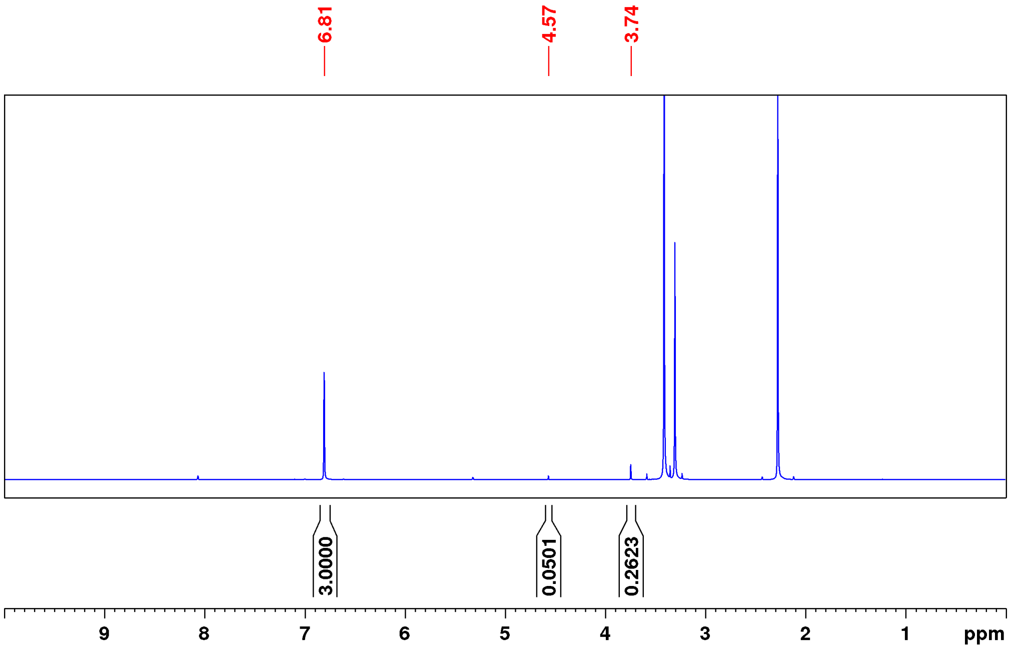

Supplementary Figure 36: Experiment 2.

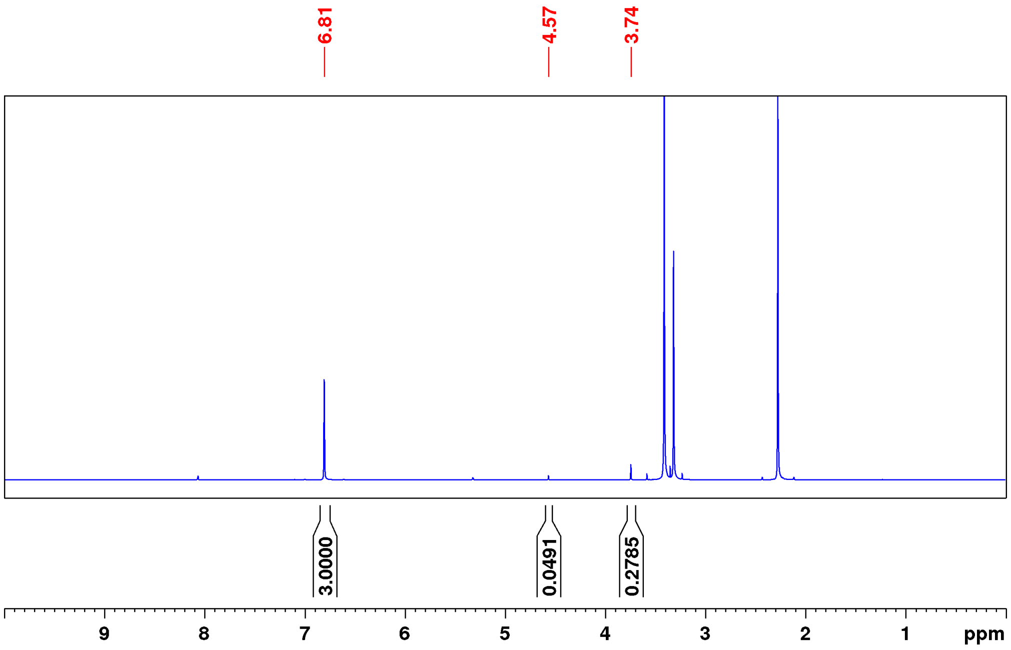

Supplementary Figure 37: Experiment 3.

5.5.3 Second Triple

$$T = 100\,°C \qquad n_{cat} = 0.075\ \mu mol$$
$$p_{H2} = 80\,bar \qquad n_{add} = 3.576\ \mu mol$$
$$p_{CO2} = 25\,bar \qquad add = Al(OTf)_3$$
$$t = 20\,h \qquad V_{MeOH} = 0.55\ mL$$

Supplementary Table 13: Catalysis results of the second triple.

	$Integral_{DMM}$	TON_{DMM}	$Integral_{MF}$	TON_{MF}	Ratio
Experiment 1	0.0584	1078	0.2386	2935	0.37
Experiment 2	0.0567	1046	0.2342	2881	0.36
Experiment 3	0.0770	1421	0.2432	2991	0.47
Average	-	1181(208)	-	2936(55)	0.40(0.06)

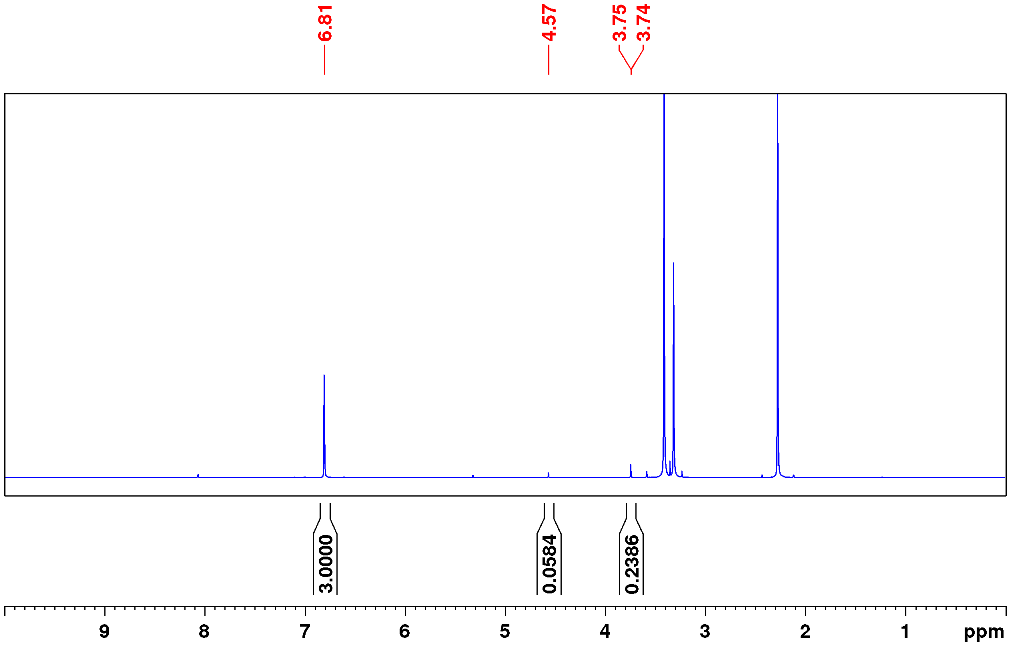

Supplementary Figure 38: Experiment 1.

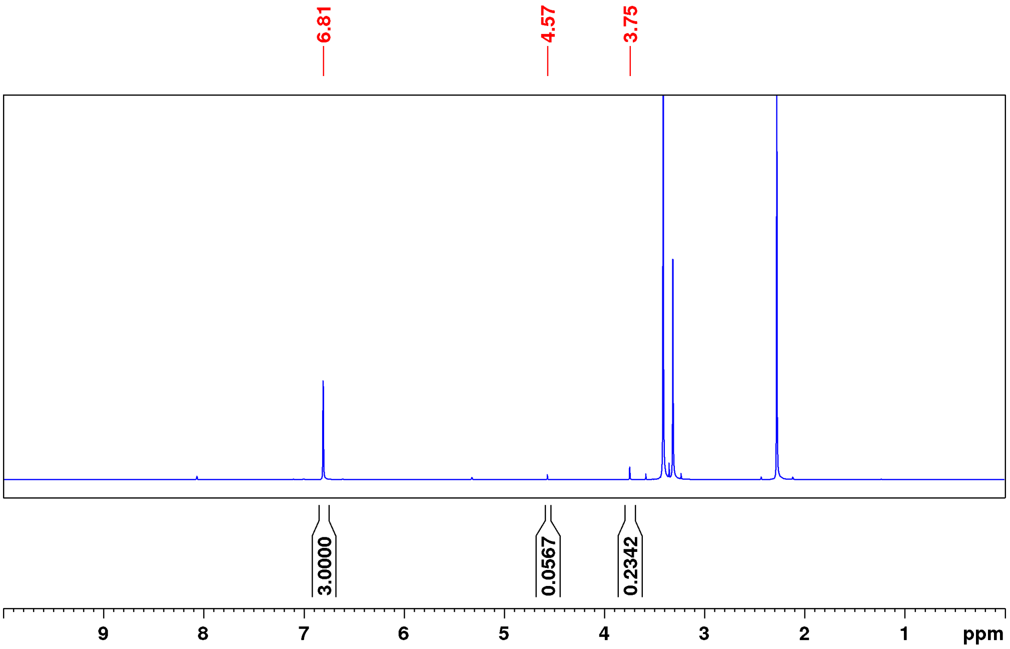

Supplementary Figure 39: Experiment 2.

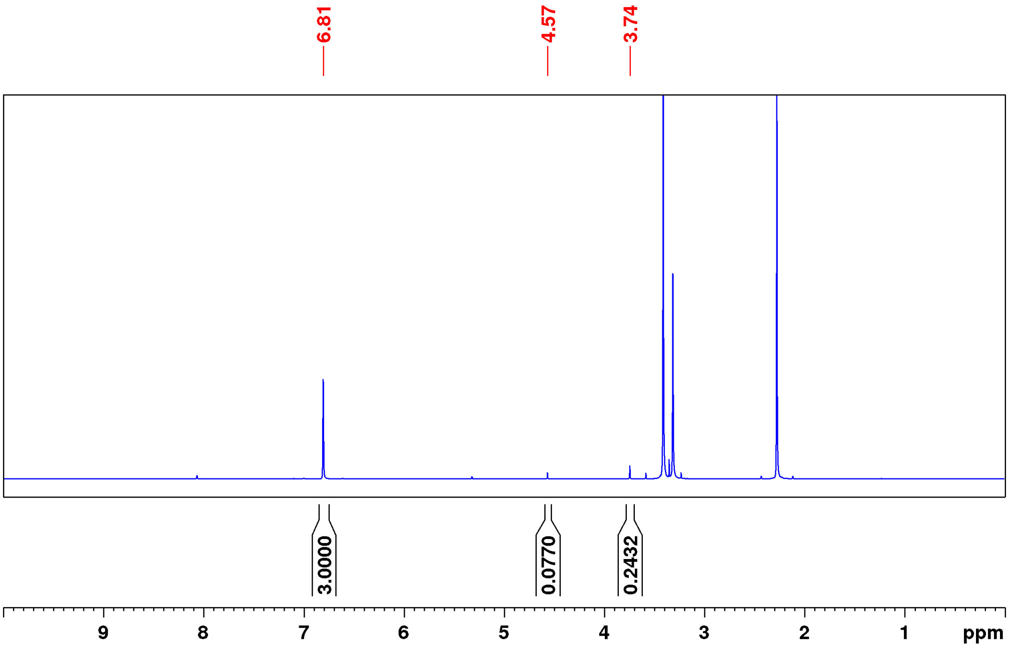

Supplementary Figure 40: Experiment 3.

5.5.4 Third Triple

$T = 80\,°C$	$n_{cat} = 0.075\ \mu mol$
$p_{H2} = 80\,bar$	$n_{add} = 3.576\ \mu mol$
$p_{CO2} = 15\,bar$	$add = Al(OTf)_3$
$t = 16\,h$	$V_{MeOH} = 0.55\ mL$

Supplementary Table 14: Catalysis results of the third triple.

	$Integral_{DMM}$	TON_{DMM}	$Integral_{MF}$	TON_{MF}	Ratio
Experiment 1	0.0231	426	0.1580	1943	0.22
Experiment 2	0.0173	319	0.1403	1726	0.18
Experiment 3	0.0353	651	0.1885	2319	0.28
Average	-	466(170)	-	1996(300)	0.23(0.05)

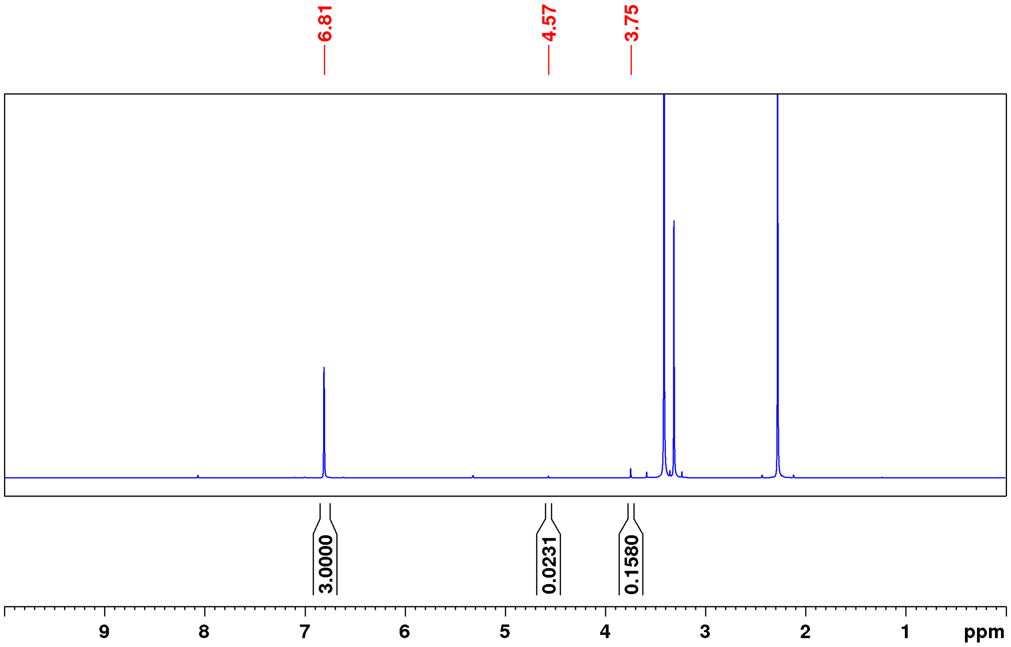

Supplementary Figure 41: Experiment 1.

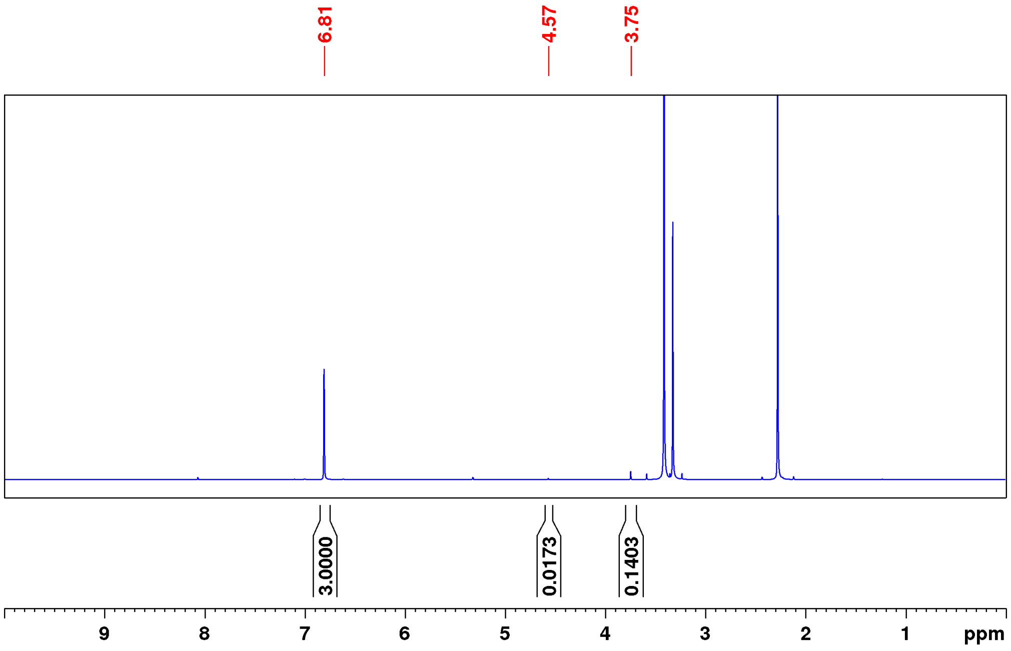

Supplementary Figure 42: Experiment 2.

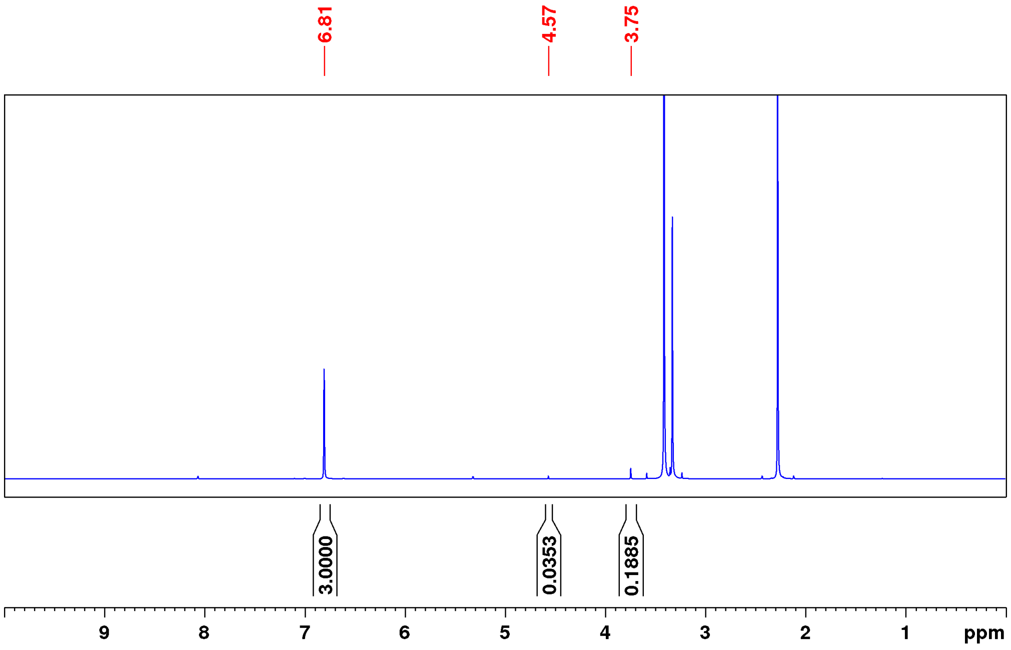

Supplementary Figure 43: Experiment 3.

5.5.5 Fourth Triple

$T = 100\,°C$ $n_{cat} = 0.075$ µmol
$p_{H2} = 100\,bar$ $n_{add} = 3.576$ µmol
$p_{CO2} = 15\,bar$ add $= Al(OTf)_3$
$t = 20\,h$ $V_{MeOH} = 0.55$ mL

Supplementary Table 15: Catalysis results of the fourth triple.

	$Integral_{DMM}$	TON_{DMM}	$Integral_{MF}$	TON_{MF}	Ratio
Experiment 1	0.1328	2450	0.1992	2450	1.00
Experiment 2	0.1247	2301	0.1915	2356	0.98
Experiment 3	0.1669	3079	0.1840	2263	1.36
Average	-	2610(413)	-	2356(93)	1.11(0.22)

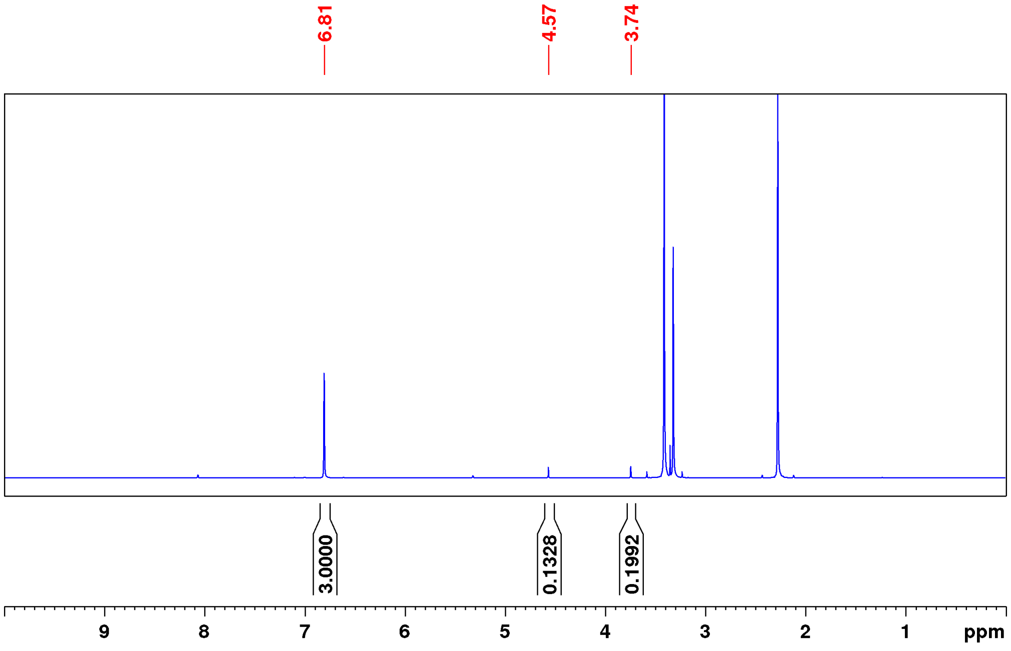

Supplementary Figure 44: Experiment 1.

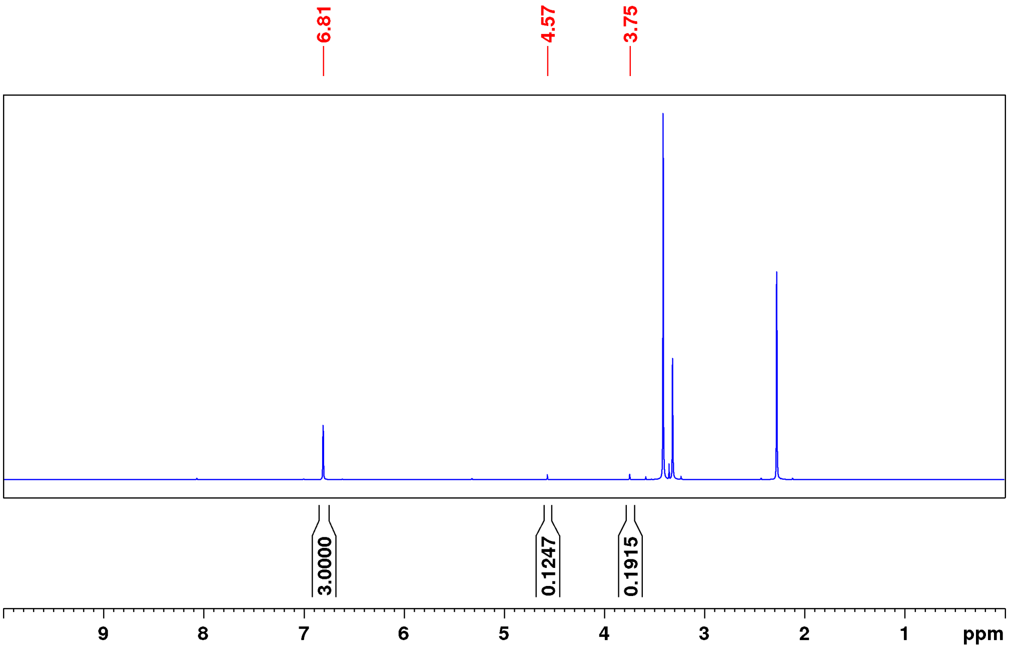

Supplementary Figure 45: Experiment 2.

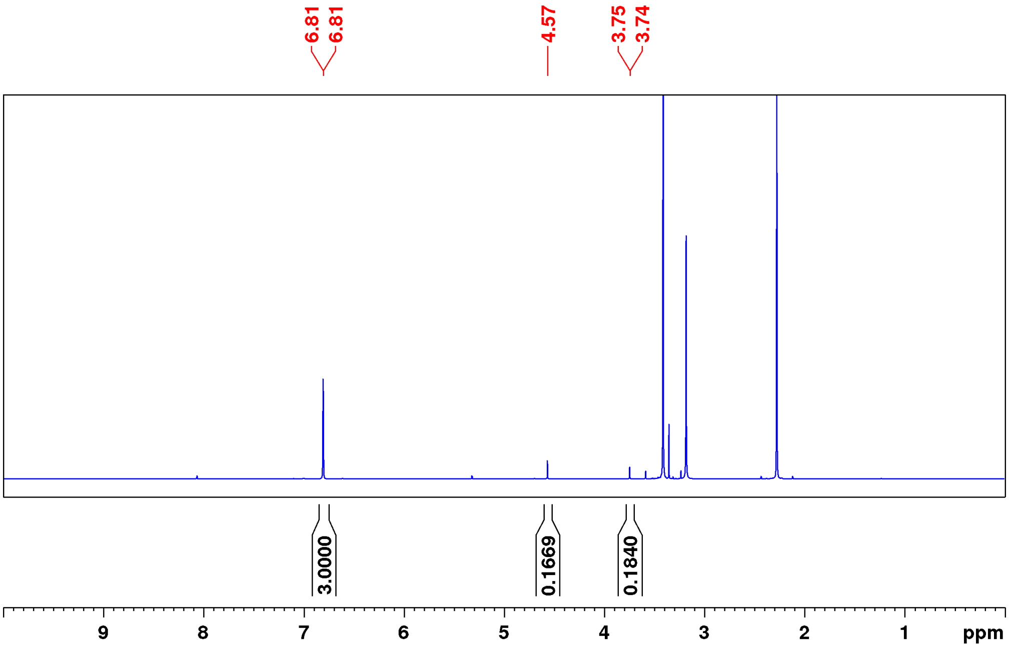

Supplementary Figure 46: Experiment 3.

5.5.6 Fifth Triple

$$T = 100\,°C \qquad n_{cat} = 0.075\ \mu mol$$
$$p_{H2} = 100\,bar \qquad n_{add} = 3.576\ \mu mol$$
$$p_{CO2} = 25\,bar \qquad add = Al(OTf)_3$$
$$t = 16\,h \qquad V_{MeOH} = 0.55\ mL$$

Supplementary Table 16: Catalysis results of the fifth triple.

	$Integral_{DMM}$	TON_{DMM}	$Integral_{MF}$	TON_{MF}	Ratio
Experiment 1	0.0825	1522	0.2396	2947	0.52
Experiment 2	0.1110	2048	0.2687	3305	0.62
Experiment 3	0.0973	1795	0.2694	3314	0.54
Average	-	1788(263)	-	3189(209)	0.56(0.05)

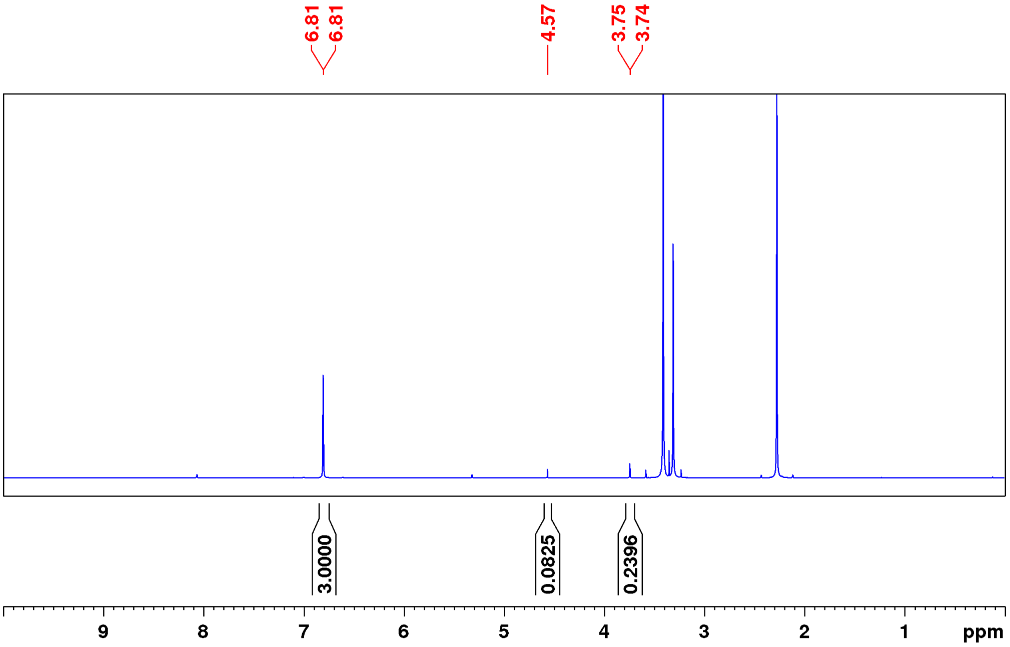

Supplementary Figure 47: Experiment 1.

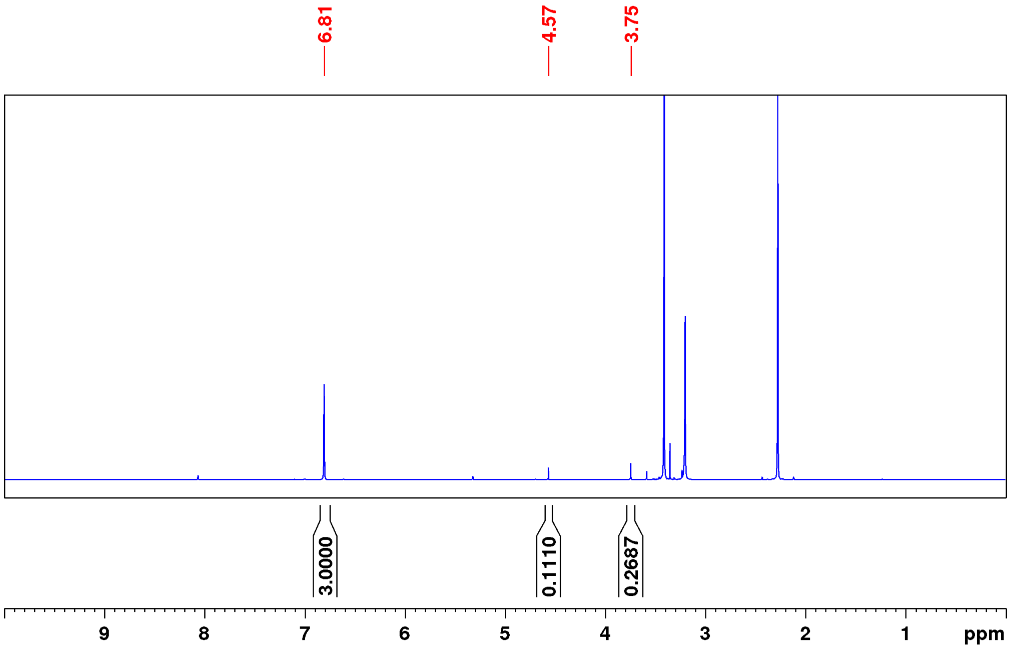

Supplementary Figure 48: Experiment 2.

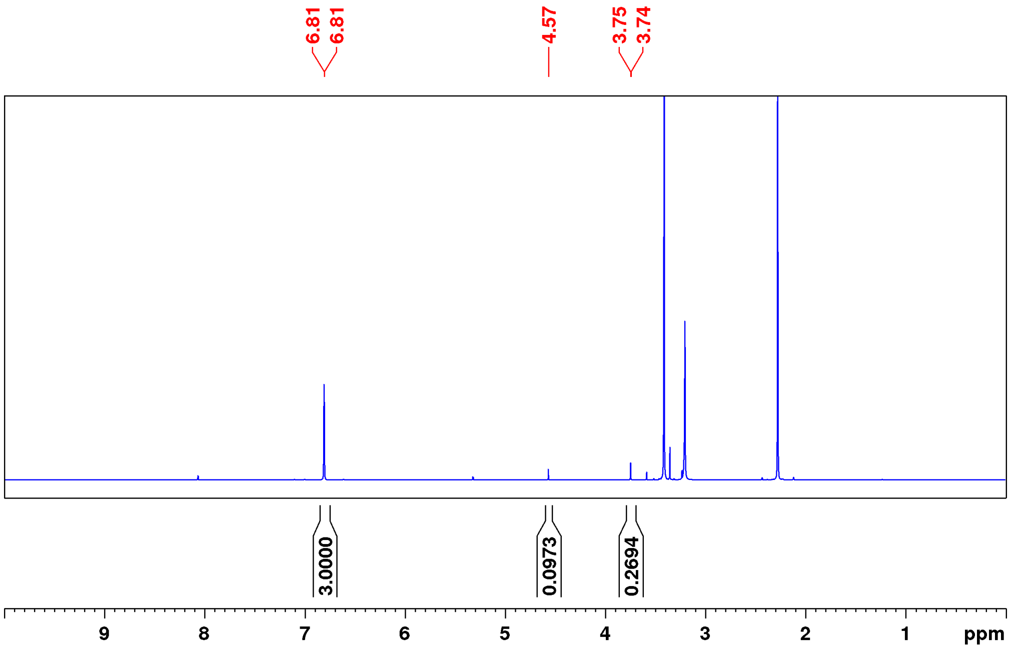

Supplementary Figure 49: Experiment 3.

5.5.7 Replication: Best Hit First Relaxation

$$T = 90\,°C \qquad n_{cat} = 0.075\ \mu mol$$
$$p_{H2} = 90\,bar \qquad n_{add} = 3.576\ \mu mol$$
$$p_{CO2} = 20\,bar \qquad add = Al(OTf)_3$$
$$t = 18\,h \qquad V_{MeOH} = 0.55\ mL$$

Supplementary Table 17: Catalysis results of the replication study.

	$Integral_{DMM}$	TON_{DMM}	$Integral_{MF}$	TON_{MF}	Ratio
Experiment 1	0.0633	1168	0.1954	2404	0.49
Experiment 2	0.0729	1345	0.2575	3167	0.42
Experiment 3	0.0740	1365	0.2569	3160	0.43
Average	-	1293(109)	-	2910(439)	0.45(0.03)

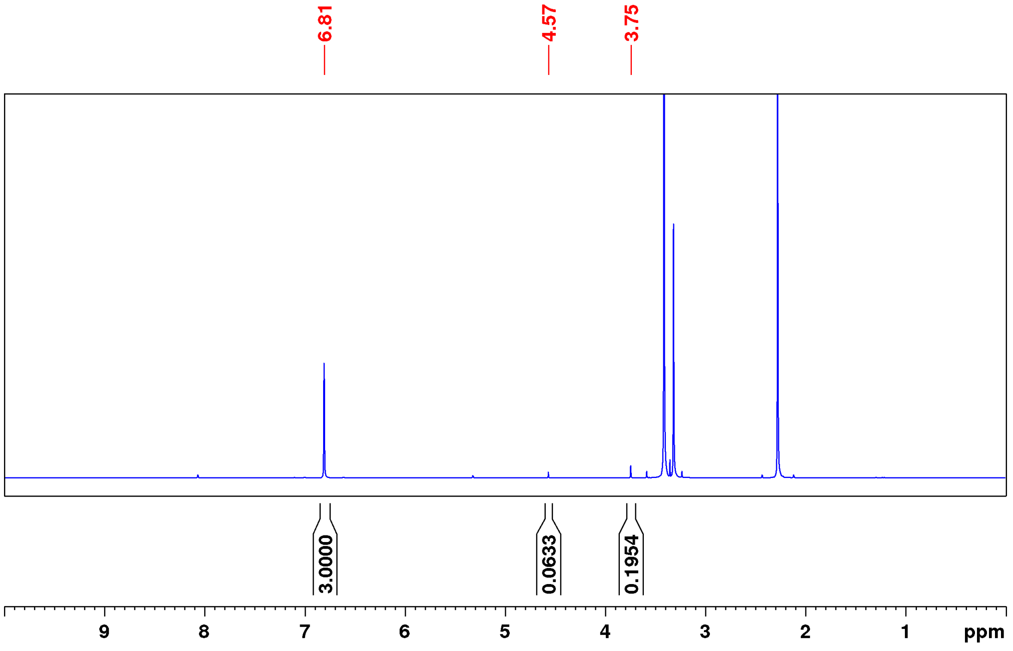

Supplementary Figure 50: Experiment 1.

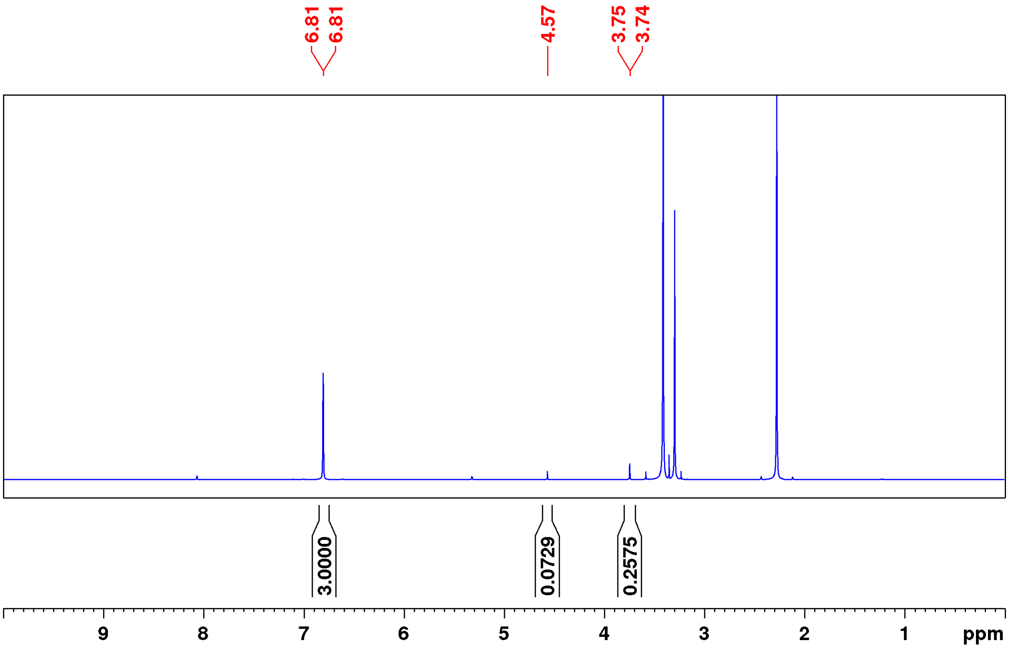

Supplementary Figure 51: Experiment 2.

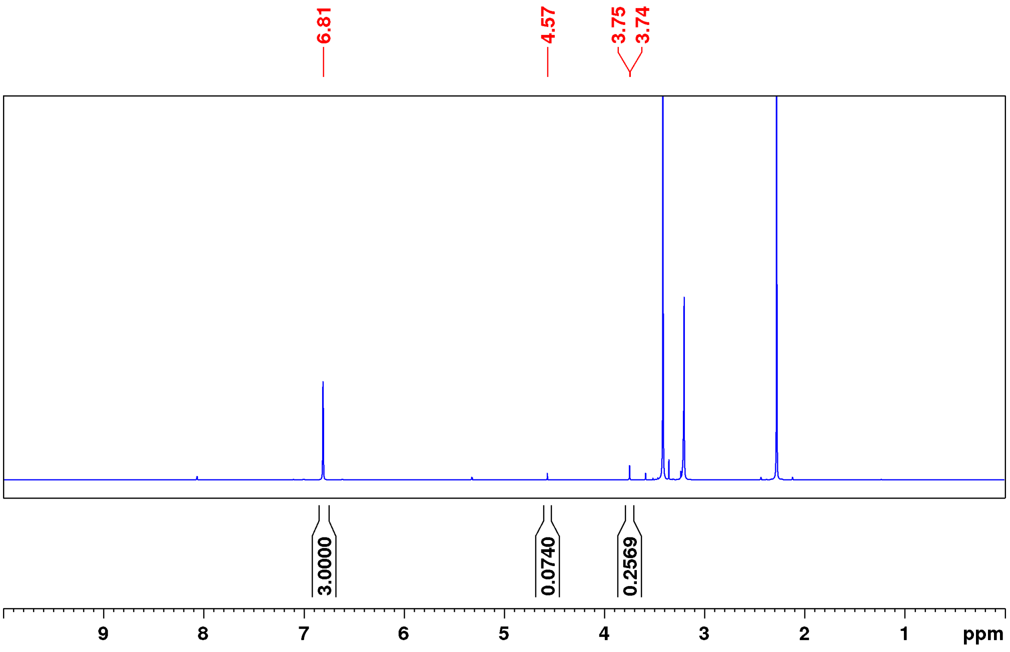

Supplementary Figure 52: Experiment 3.

5.6 Second Relaxation

5.6.1 Replication: Best Hit Second DoE

$$T = 100\,°\mathrm{C} \qquad n_{cat} = 0.075\ \mu\mathrm{mol}$$
$$p_{H2} = 100\,\mathrm{bar} \qquad n_{add} = 3.576\ \mu\mathrm{mol}$$
$$p_{CO2} = 15\,\mathrm{bar} \qquad \mathrm{add} = \mathrm{Al(OTf)_3}$$
$$t = 20\,\mathrm{h} \qquad V_{MeOH} = 0.55\ \mathrm{mL}$$

Supplementary Table 18: Catalysis results of the replication study.

	$\mathrm{Integral_{DMM}}$	$\mathrm{TON_{DMM}}$	$\mathrm{Integral_{MF}}$	$\mathrm{TON_{MF}}$	Ratio
Experiment 1	0.1519	2803	0.1813	2230	1.26
Experiment 2	0.1306	2410	0.1973	2427	0.99
Experiment 3	0.1343	2478	0.2071	2547	0.97
Average	-	2563(210)	-	2401(160)	1.07(0.16)

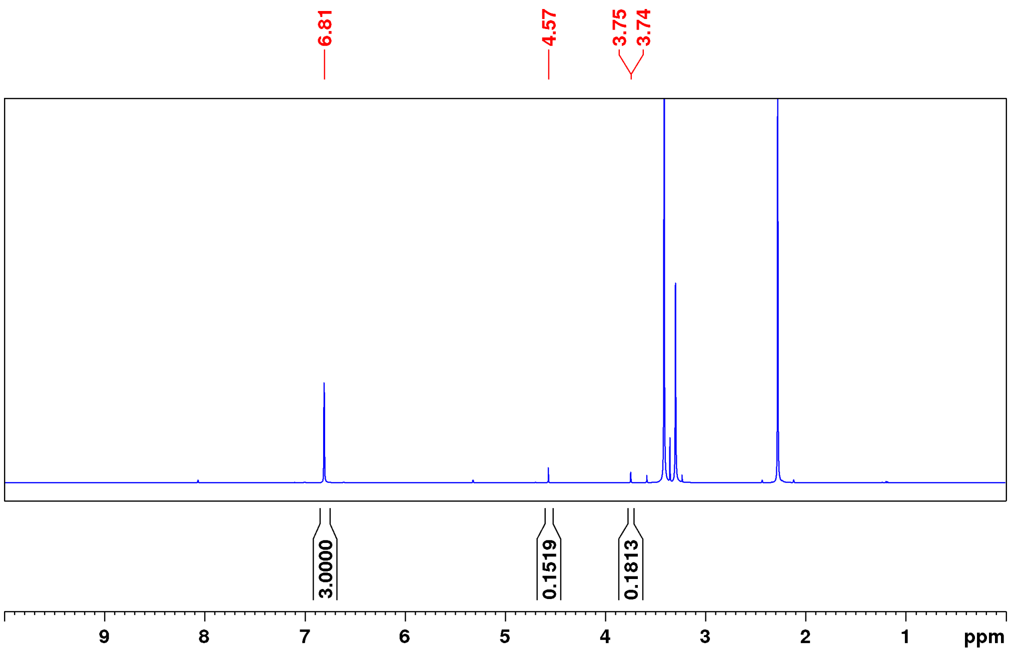

Supplementary Figure 53: Experiment 1.

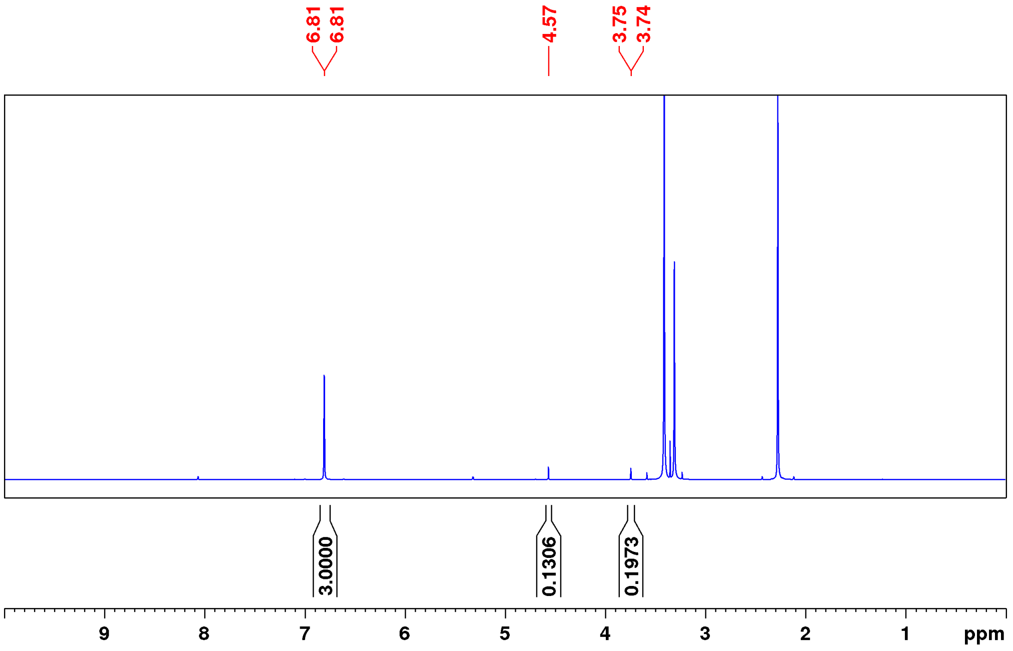

Supplementary Figure 54: Experiment 2.

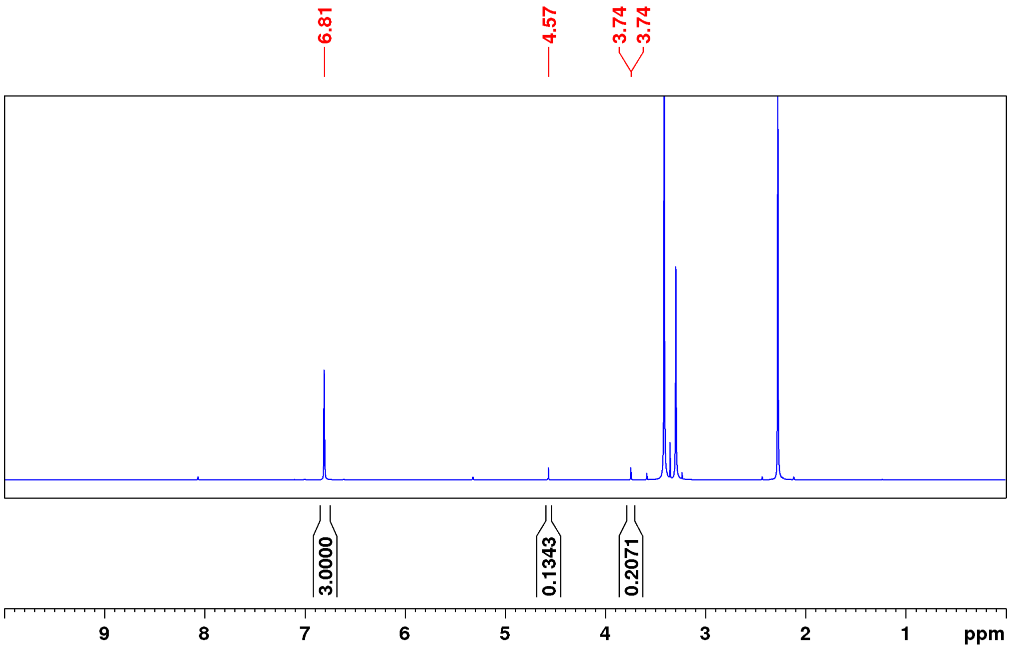

Supplementary Figure 55: Experiment 3.

5.6.2 25 % Relaxation

$$T = 105\,°C \qquad n_{cat} = 0.075\ \mu mol$$
$$p_{H2} = 105\,bar \qquad n_{add} = 3.576\ \mu mol$$
$$p_{CO2} = 12.5\,bar \qquad add = Al(OTf)_3$$
$$t = 21\,h \qquad V_{MeOH} = 0.55\ mL$$

Supplementary Table 19: Catalysis results of the 25 % relaxation trials.

	$Integral_{DMM}$	TON_{DMM}	$Integral_{MF}$	TON_{MF}	Ratio
Experiment 1	0.1406	2594	0.1489	1832	1.42
Experiment 2	0.1470	2712	0.1390	1710	1.59
Experiment 3	0.1613	2976	0.1435	1765	1.69
Average	-	2761(196)	-	1769(61)	1.56(0.14)

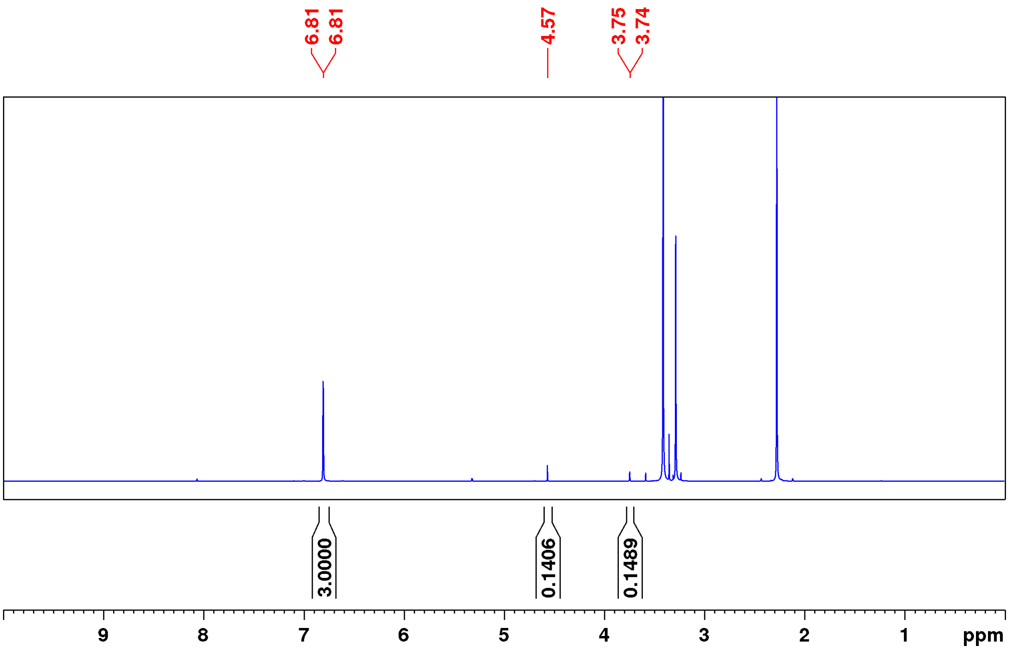

Supplementary Figure 56: Experiment 1.

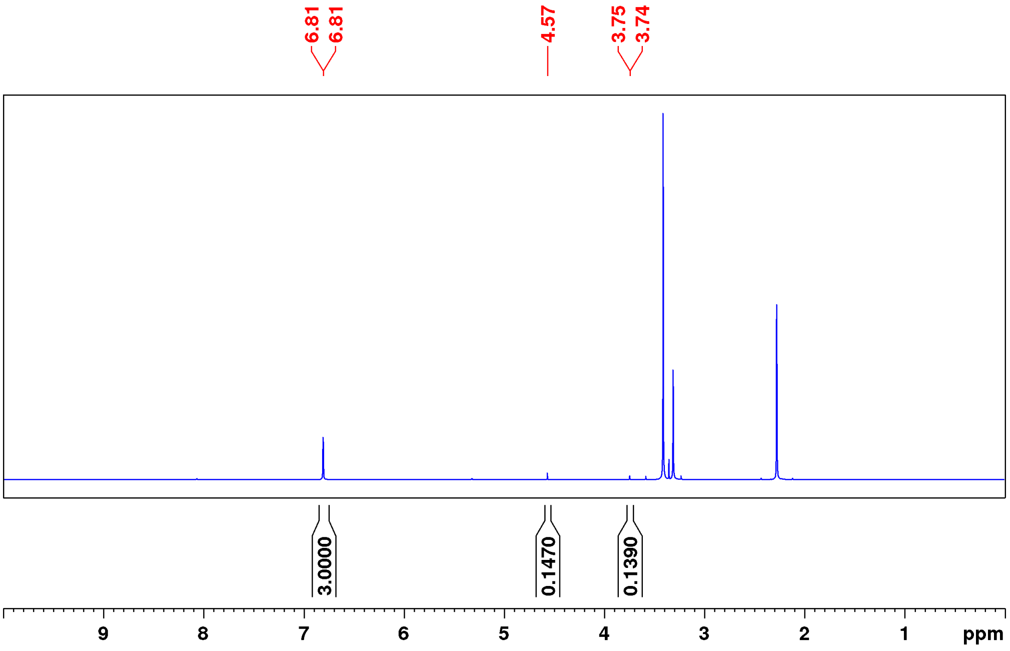

Supplementary Figure 57: Experiment 2.

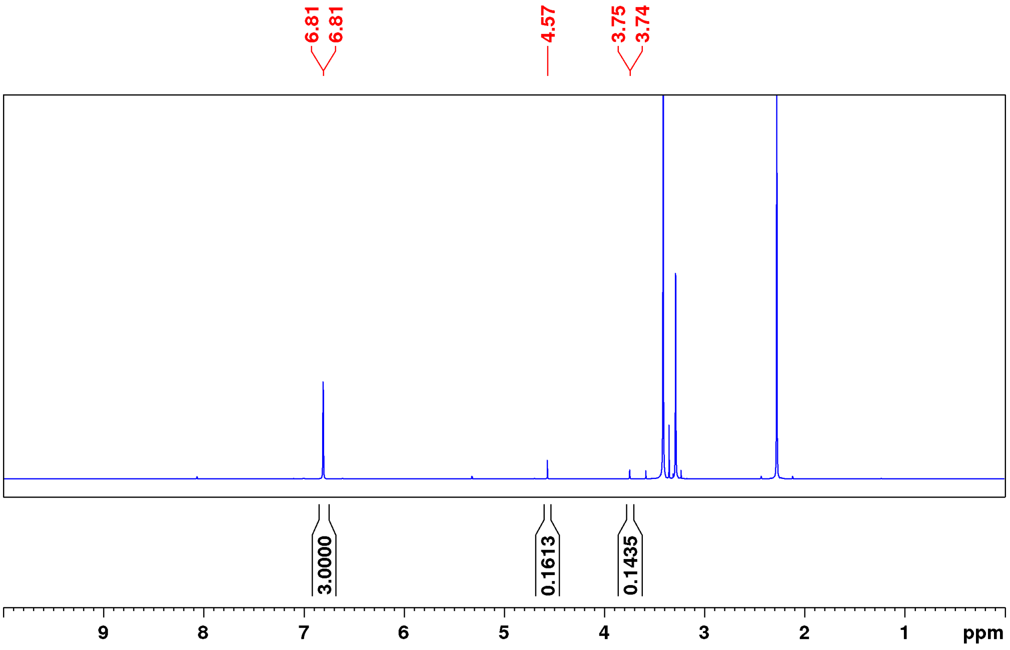

Supplementary Figure 58: Experiment 3.

5.6.3 50 % Relaxation

$$T = 110\,°C \qquad n_{cat} = 0.075\ \mu mol$$
$$p_{H2} = 110\,bar \qquad n_{add} = 3.576\ \mu mol$$
$$p_{CO2} = 10\,bar \qquad add = Al(OTf)_3$$
$$t = 22\,h \qquad V_{MeOH} = 0.55\ mL$$

Supplementary Table 20: Catalysis results of the 50 % relaxation trials.

	$Integral_{DMM}$	TON_{DMM}	$Integral_{MF}$	TON_{MF}	Ratio
Experiment 1	0.1165	2150	0.1069	1315	1.63
Experiment 2	0.1245	2297	0.1080	1328	1.73
Experiment 3	0.1181	2179	0.0962	1183	1.84
Average	-	2209(78)	-	1276(80)	1.74(0.10)

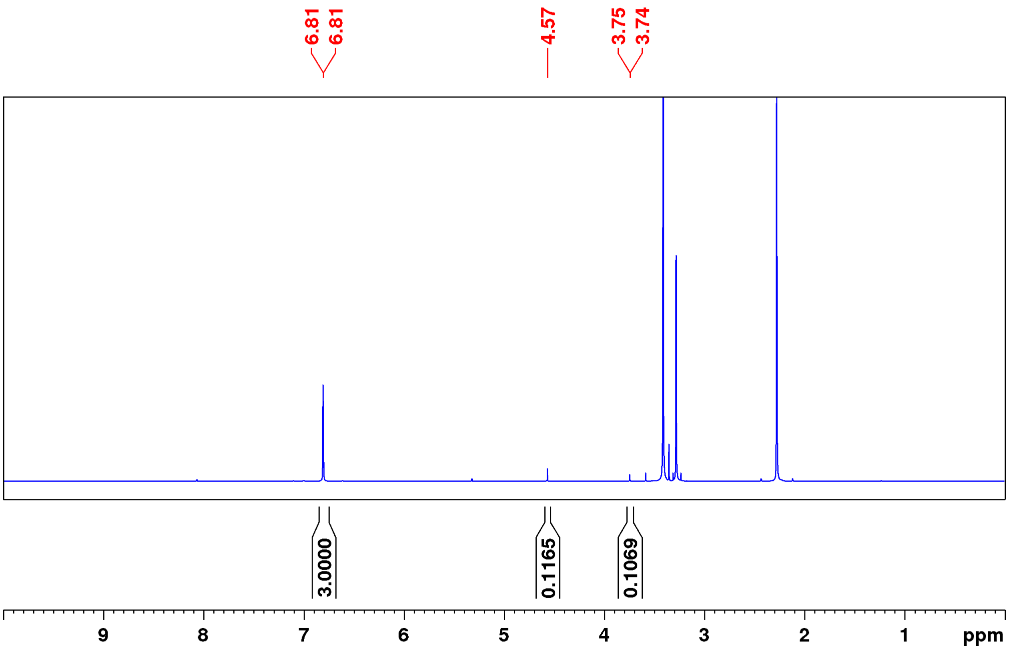

Supplementary Figure 59: Experiment 1.

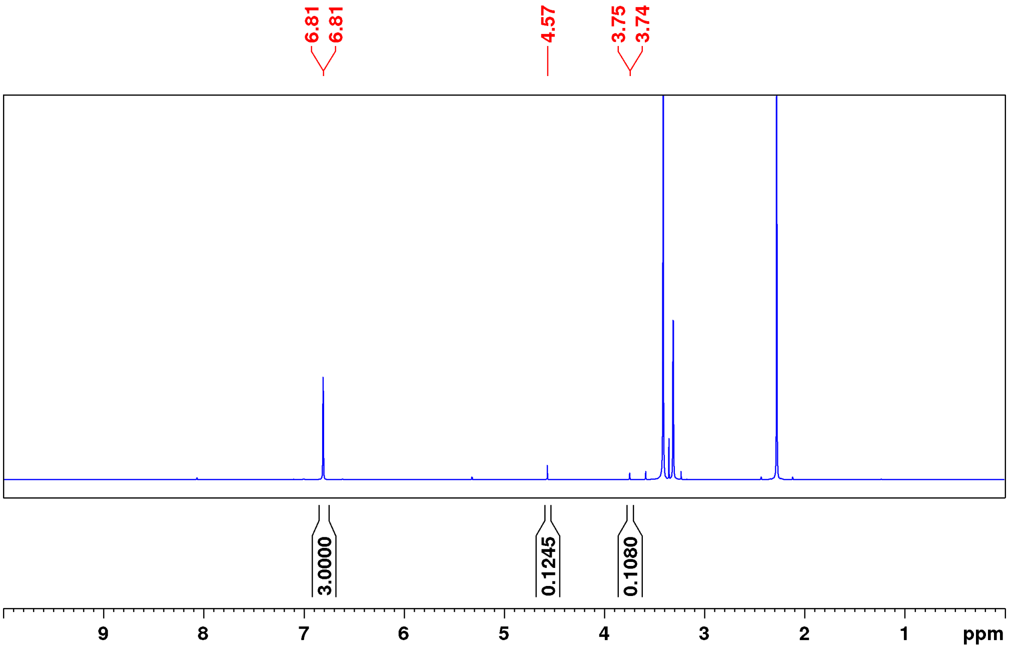

Supplementary Figure 60: Experiment 2.

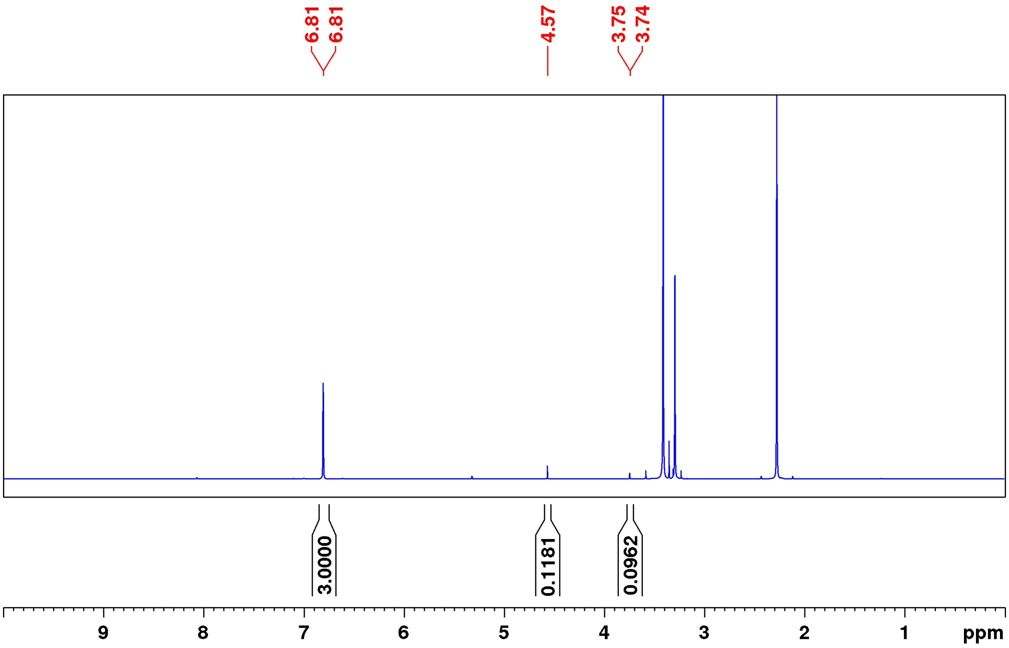

Supplementary Figure 61: Experiment 3.

5.7 Upscale Reactions

$$T = 100\,°\text{C} \qquad n_{cat} = 0.75\,\mu\text{mol}$$
$$p_{H2} = 100\,\text{bar} \qquad n_{add} = 35.76\,\mu\text{mol}$$
$$p_{CO2} = 15\,\text{bar} \qquad \text{add} = \text{Al(OTf)}_3$$
$$t = 20\,\text{h} \qquad V_{MeOH} = 5.5\,\text{mL}$$

Supplementary Table 21: Catalysis results of the upscale reactions.

	$Integral_{DMM}$	TON_{DMM}	$Integral_{MF}$	TON_{MF}	Ratio
Experiment 1	0.2082	3841	0.1353	1664	2.31
Experiment 2	0.2117	3906	0.0996	1225	3.19
Average	-	3874(46)	-	1445(311)	2.75(0.62)

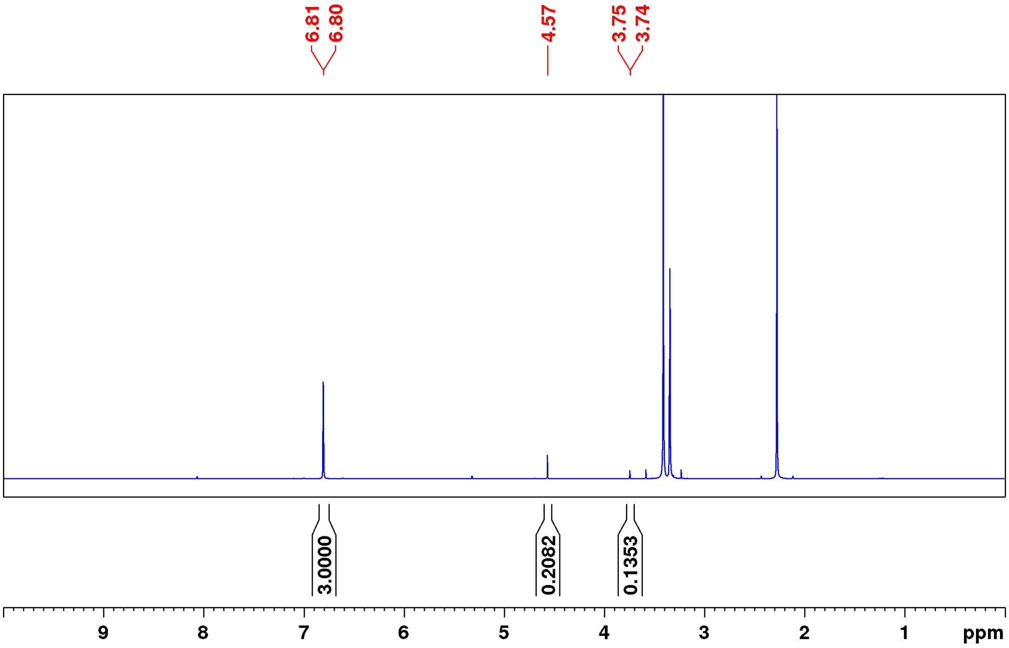

Supplementary Figure 62: Experiment 1.

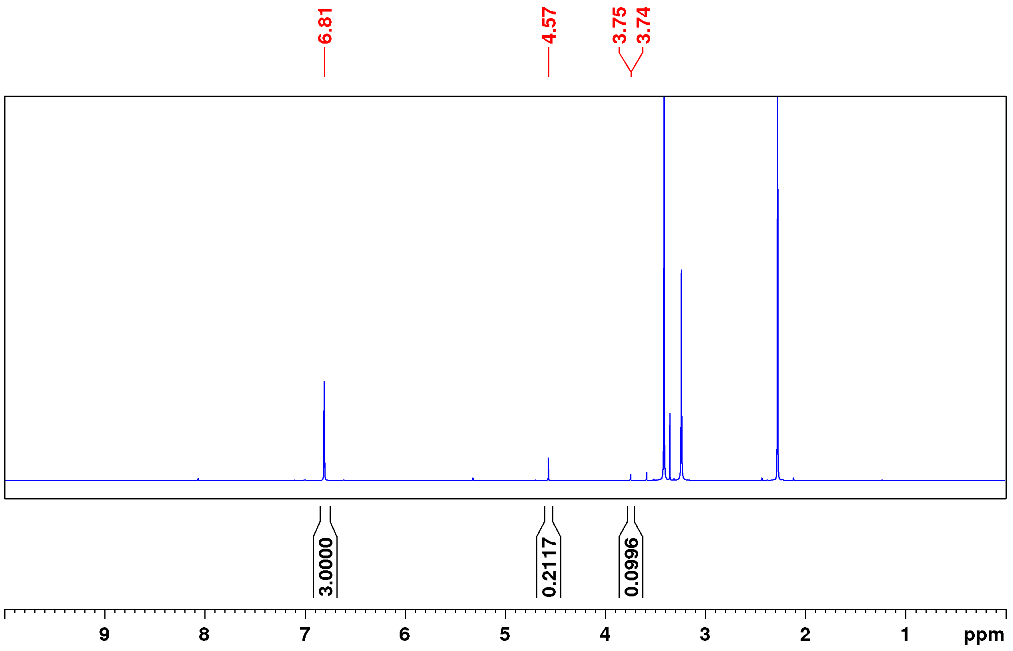

Supplementary Figure 63: Experiment 2.

5.8 Background Measurements

Run without Catalyst and Co-Catalyst[17]

$$T = 90\,°\text{C} \qquad n_{cat} = 0.00\ \mu\text{mol}$$
$$p_{H2} = 90\,\text{bar} \qquad n_{add} = 0.00\ \mu\text{mol}$$
$$p_{CO2} = 20\,\text{bar} \qquad \text{add} = \text{none}$$
$$t = 18\,\text{h} \qquad V_{MeOH} = 0.5\ \text{mL}$$

Supplementary Table 22: Run without catalyst and co-catalyst.

	Integral_{DMM}	n_{DMM} (µmol)	Integral_{MF}	n_{MF} (µmol)	Ratio
Experiment 1	0.0001	0.13	0.0051	4.28	0.03
Experiment 2	0.0003	0.38	0.0022	1.85	0.20
Average	-	0.25(0.18)	-	3.06(1.72)	0.12(0.12)

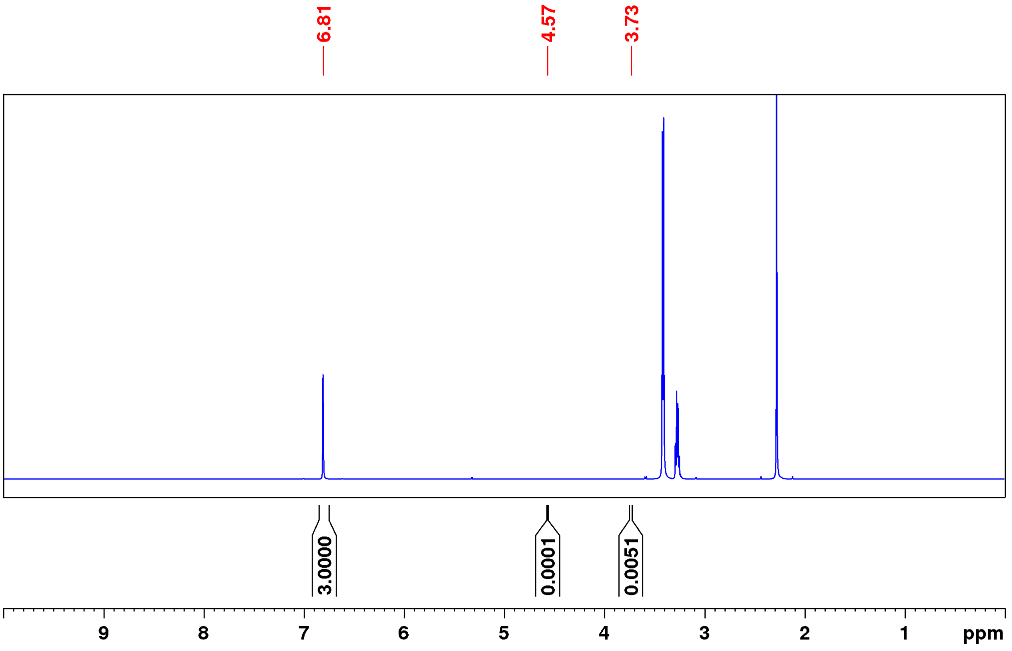

Supplementary Figure 64: Experiment 1.

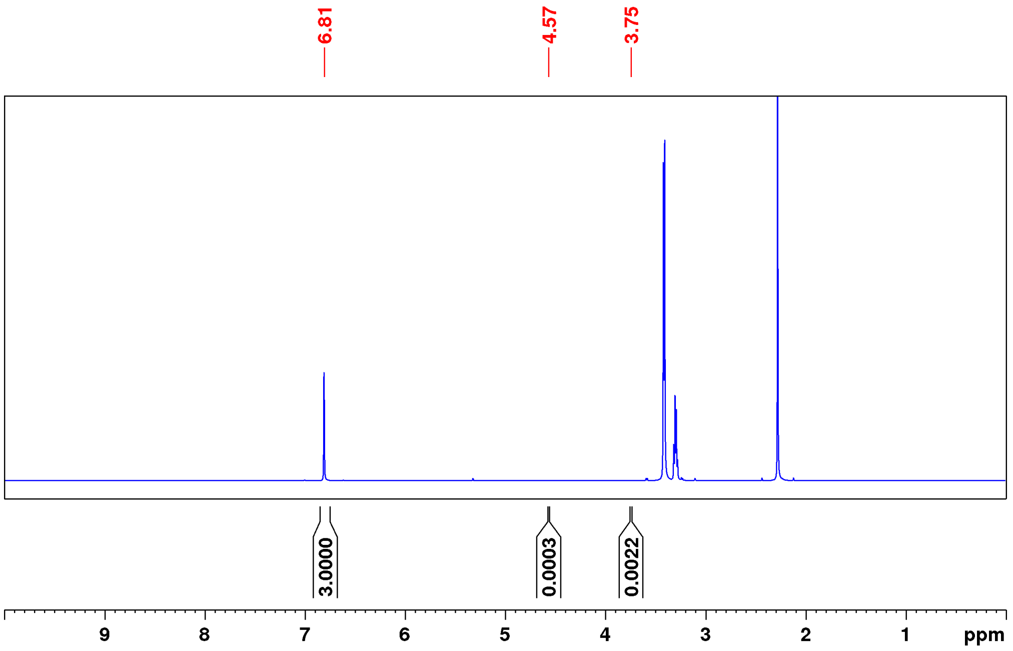

Supplementary Figure 65: Experiment 2.

Run without Co-Catalyst[17]

$$T = 90\,°\mathrm{C} \qquad n_{cat} = 1.50\ \mu\mathrm{mol}$$
$$p_{H2} = 90\,\mathrm{bar} \qquad n_{add} = 0.00\ \mu\mathrm{mol}$$
$$p_{CO2} = 20\,\mathrm{bar} \qquad \mathrm{add} = \mathrm{none}$$
$$t = 18\,\mathrm{h} \qquad V_{MeOH} = 0.5\ \mathrm{mL}$$

Supplementary Table 23: Run without co-catalyst.

	$Integral_{DMM}$	TON_{DMM}	$Integral_{MF}$	TON_{MF}	Ratio
Experiment 1	0.0000	0	0.0069	4	0.00
Experiment 2	0.0004	0	0.0100	6	0.06
Average	-	0(0)	-	5(1)	0.03(0.04)

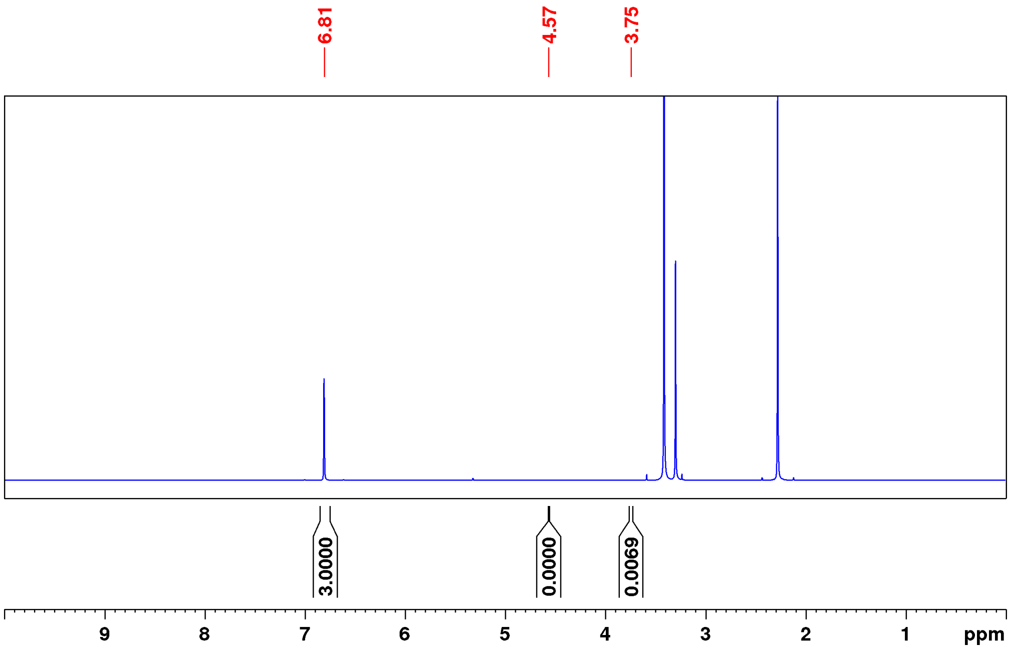

Supplementary Figure 66: Experiment 1.

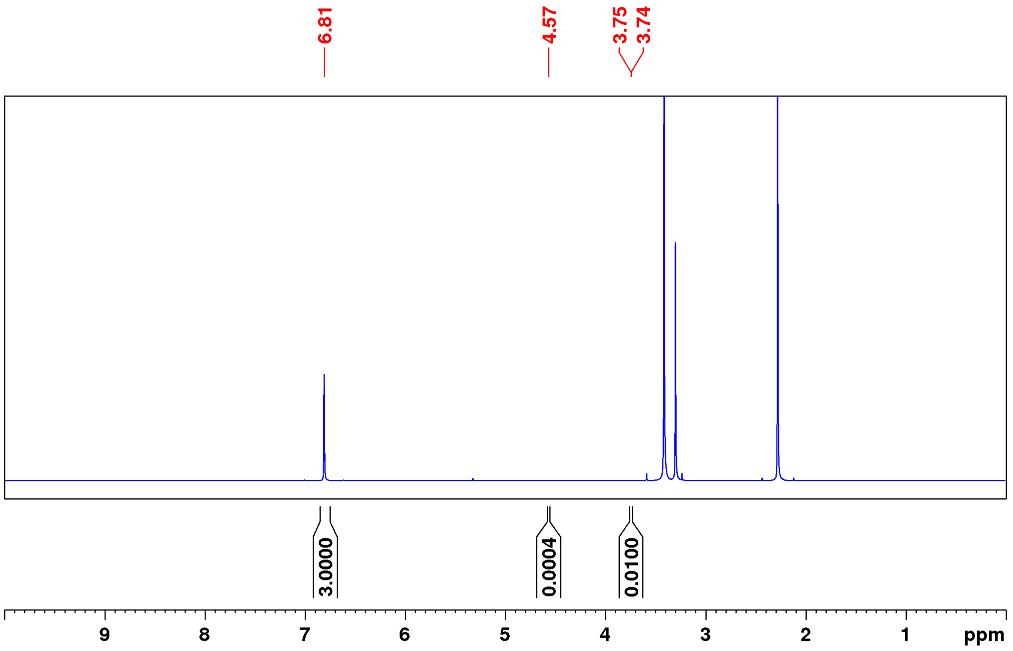

Supplementary Figure 67: Experiment 2.

Run without Catalyst[17]

$$T = 90\,°C \qquad n_{cat} = 0.00\ \mu mol$$
$$p_{H2} = 90\,bar \qquad n_{add} = 6.25\ \mu mol$$
$$p_{CO2} = 20\,bar \qquad add = Al(OTf)_3$$
$$t = 18\,h \qquad V_{MeOH} = 0.5\ mL$$

Supplementary Table 24: Run without catalyst.

	$Integral_{DMM}$	n_{DMM} (µmol)	$Integral_{MF}$	n_{MF} (µmol)	Ratio
Experiment 1	0.0000	0.00	0.0041	3.44	0.00
Experiment 2	0.0003	0.38	0.0049	4.11	0.09
Average	-	0.19(0.27)	-	3.77(0.47)	0.05(0.06)

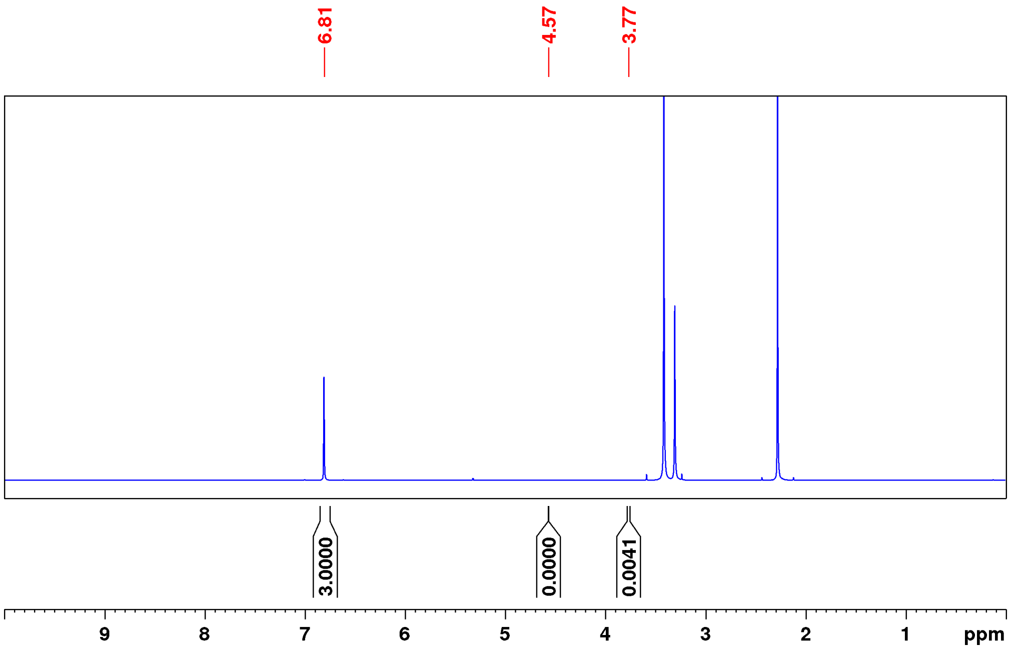

Supplementary Figure 68: Experiment 1.

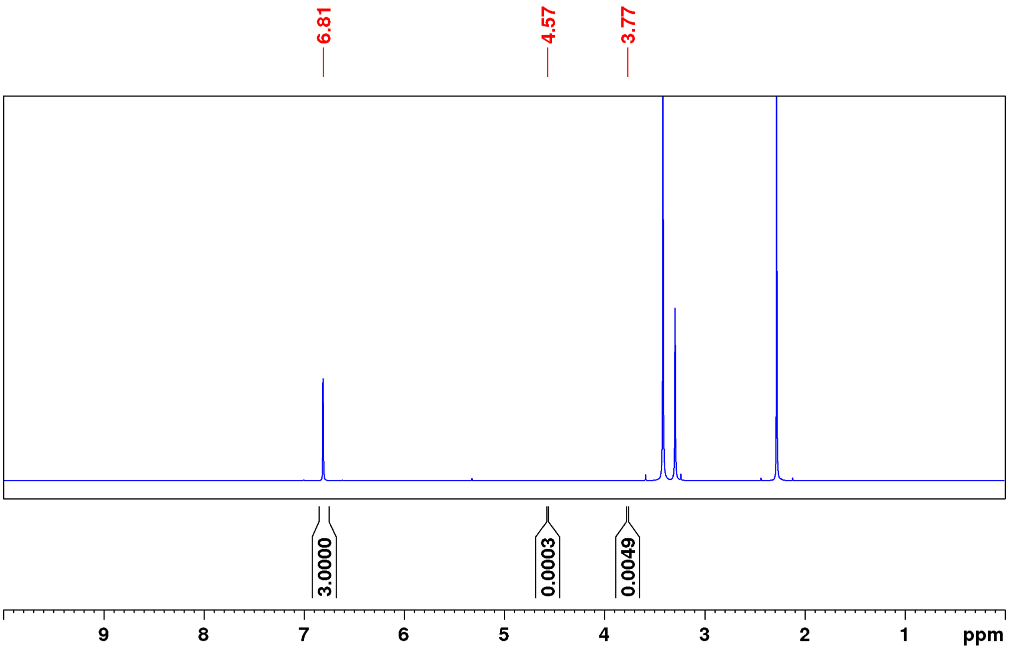

Supplementary Figure 69: Experiment 2.

5.9 Overview of Catalysis Results

All catalysis results from the first DoE (Section 5.3), the first relaxation (Section 5.4), the second DoE (Section 5.5), the second relaxation (Section 5.6) and the reactions performed in the tailor-made experimental setup (Section 5.7) are given in Supplementary Table 25.

Supplementary Table 25: Overview of the catalysis results.

	entry	Catalysis Process Parameters								Responses	
		T (°C)	p_{H2} (bar)	p_{CO2} (bar)	t (h)	additive	n_{cat} (µmol)	n_{add} (µmol)	V_{MeOH} (mL)	TON DMM	TON MF
First DoE	1	90	90	20	18	$Al(OTf)_3$	0.75	0.78125	0.25	195	89
	2	90	90	20	18	$Al(OTf)_3$	0.75	0.78125	0.25	196	85
	3	90	90	20	18	$Al(OTf)_3$	0.75	0.78125	0.25	220	95
	4	90	90	20	18	$Al(OTf)_3$	0.75	0.78125	0.5	124	210
	5	90	90	20	18	$Al(OTf)_3$	0.75	0.78125	0.5	114	191
	6	90	90	20	18	$Al(OTf)_3$	0.75	0.78125	0.5	121	212
	7	90	90	20	18	$Al(OTf)_3$	0.75	1.953125	0.375	337	122
	8	90	90	20	18	$Al(OTf)_3$	0.75	1.953125	0.375	335	110
	9	90	90	20	18	$Al(OTf)_3$	0.75	1.953125	0.375	355	116
	10	90	90	20	18	$Al(OTf)_3$	0.1875	3.125	0.25	1162	437
	11	90	90	20	18	$Al(OTf)_3$	0.1875	3.125	0.25	1147	415
	12	90	90	20	18	$Al(OTf)_3$	0.1875	3.125	0.25	1040	458
	13	90	90	20	18	$Al(OTf)_3$	0.75	3.125	0.25	310	78
	14	90	90	20	18	$Al(OTf)_3$	0.75	3.125	0.25	308	80
	15	90	90	20	18	$Al(OTf)_3$	0.75	3.125	0.25	323	80
	16	90	90	20	18	$Al(OTf)_3$	0.46875	3.125	0.375	628	194
	17	90	90	20	18	$Al(OTf)_3$	0.46875	3.125	0.375	673	213
	18	90	90	20	18	$Al(OTf)_3$	0.46875	3.125	0.375	550	195
	19	90	90	20	18	$Al(OTf)_3$	0.1313	3.3594	0.525	1475	1706
	20	90	90	20	18	$Al(OTf)_3$	0.1313	3.3594	0.525	1071	1431

Continued on next page...

Supplementary Table 25: Overview of the catalysis results. (continued)

	entry	Catalysis Process Parameters								Responses	
		T (°C)	p_{H2} (bar)	p_{CO2} (bar)	t (h)	additive	n_{cat} (µmol)	n_{add} (µmol)	V_{MeOH} (mL)	TON DMM	TON MF
First relaxation	21	90	90	20	18	Al(OTf)$_3$	0.1313	3.3594	0.525	1574	1543
	22	90	90	20	18	Al(OTf)$_3$	0.075	3.576	0.55	1546	2796
	23	90	90	20	18	Al(OTf)$_3$	0.075	3.576	0.55	1517	2868
	24	90	90	20	18	Al(OTf)$_3$	0.075	3.576	0.55	1061	2621
	25	90	90	20	18	Al(OTf)$_3$	0.0188	3.6594	0.575	38	1026
	26	90	90	20	18	Al(OTf)$_3$	0.0188	3.6594	0.575	8	995
	27	90	90	20	18	Al(OTf)$_3$	0.0188	3.6594	0.575	492	1098
Second DoE	28	90	90	20	18	Al(OTf)$_3$	0.075	3.576	0.55	1415	2715
	29	90	90	20	18	Al(OTf)$_3$	0.075	3.576	0.55	1367	2741
	30	90	90	20	18	Al(OTf)$_3$	0.075	3.576	0.55	1199	2962
	31	80	100	25	20	Al(OTf)$_3$	0.075	3.576	0.55	838	3190
	32	80	100	25	20	Al(OTf)$_3$	0.075	3.576	0.55	924	3226
	33	80	100	25	20	Al(OTf)$_3$	0.075	3.576	0.55	906	3426
	34	100	80	25	20	Al(OTf)$_3$	0.075	3.576	0.55	1078	2935
	35	100	80	25	20	Al(OTf)$_3$	0.075	3.576	0.55	1046	2881
	36	100	80	25	20	Al(OTf)$_3$	0.075	3.576	0.55	1421	2991
	37	80	80	15	16	Al(OTf)$_3$	0.075	3.576	0.55	426	1943
	38	80	80	15	16	Al(OTf)$_3$	0.075	3.576	0.55	319	1726
	39	80	80	15	16	Al(OTf)$_3$	0.075	3.576	0.55	651	2319
	40	100	100	15	20	Al(OTf)$_3$	0.075	3.576	0.55	2450	2450

Continued on next page. . .

Supplementary Table 25: Overview of the catalysis results. (continued)

	entry	Catalysis Process Parameters								Responses	
		T (°C)	p_{H2} (bar)	p_{CO2} (bar)	t (h)	additive	n_{cat} (µmol)	n_{add} (µmol)	V_{MeOH} (mL)	TON DMM	TON MF
	41	100	100	15	20	Al(OTf)$_3$	0.075	3.576	0.55	2301	2356
	42	100	100	15	20	Al(OTf)$_3$	0.075	3.576	0.55	3079	2263
	43	100	100	25	16	Al(OTf)$_3$	0.075	3.576	0.55	1522	2947
	44	100	100	25	16	Al(OTf)$_3$	0.075	3.576	0.55	2048	3305
	45	100	100	25	16	Al(OTf)$_3$	0.075	3.576	0.55	1795	3314
	46	90	90	20	18	Al(OTf)$_3$	0.075	3.576	0.55	1168	2404
	47	90	90	20	18	Al(OTf)$_3$	0.075	3.576	0.55	1345	3167
	48	90	90	20	18	Al(OTf)$_3$	0.075	3.576	0.55	1365	3160
Second relaxation	49	100	100	15	20	Al(OTf)$_3$	0.075	3.576	0.55	2803	2230
	50	100	100	15	20	Al(OTf)$_3$	0.075	3.576	0.55	2410	2427
	51	100	100	15	20	Al(OTf)$_3$	0.075	3.576	0.55	2478	2547
	52	105	105	12.5	21	Al(OTf)$_3$	0.075	3.576	0.55	2594	1832
	53	105	105	12.5	21	Al(OTf)$_3$	0.075	3.576	0.55	2712	1710
	54	105	105	12.5	21	Al(OTf)$_3$	0.075	3.576	0.55	2976	1765
	55	110	110	10	22	Al(OTf)$_3$	0.075	3.576	0.55	2150	1315
	56	110	110	10	22	Al(OTf)$_3$	0.075	3.576	0.55	2297	1328
	57	110	110	10	22	Al(OTf)$_3$	0.075	3.576	0.55	2179	1183
Upscale	58	100	100	15	20	Al(OTf)$_3$	0.75	35.76	5.5	3841	1664
	59	100	100	15	20	Al(OTf)$_3$	0.75	35.76	5.5	3906	1225

6 Additional Information

6.1 Simple Spearman Rank Correlation Analysis

Supplementary Table 26 lists the simple Spearman rank correlation analysis between SD(TON_{DMM}), SD(TON_{MF}) and the process parameters X_i.

Supplementary Table 26: Simple Spearman rank correlation analysis.

SD	T	p_{H2}	p_{CO2}	t	n_{cat}	n_{add}	V
TON_{DMM}	0.33	0.04	-0.09	0.01	-0.76	0.64	0.73
TON_{MF}	-0.06	0.09	0.02	-0.17	-0.73	0.47	0.74

6.2 Replication Error

Supplementary Table 27 (summary of Supplementary Table 11 and Supplementary Table 17) lists the six replication experiments of the best hit of the first relaxation shown in Supplementary Table 28 (see Supplementary Table 9).

Supplementary Table 29 (see Supplementary Table 18) lists the three replication experiments of the best hit of the second DoE shown in Supplementary Table 30 (see Supplementary Table 15).

The comparison of Supplementary Table 27 and Supplementary Table 28 as well as of Supplementary Table 29 and Supplementary Table 30 illustrates the low replication error and thus the good repeatability of the catalytic reaction and the reliability of the system.

Supplementary Table 27: Replication study of the best hit of the first relaxation.

	TON_{DMM}	TON_{MF}	Ratio
Experiment 1.1	1415	2715	0.52
Experiment 1.2	1367	2741	0.50
Experiment 1.3	1199	2962	0.40
Average	1327(113)	2806(136)	0.48(0.06)
Experiment 2.1	1168	2404	0.49
Experiment 2.2	1345	3167	0.42
Experiment 2.3	1365	3160	0.43
Average	1293(109)	2910(439)	0.45(0.03)
Overall Average	1310(101)	2858(296)	0.46(0.05)

Supplementary Table 28: Catalysis results of the 20 % relaxation trials of the first relaxation.

	TON_{DMM}	TON_{MF}	Ratio
Experiment 1	1546	2796	0.55
Experiment 2	1517	2868	0.53
Experiment 3	1061	2621	0.40
Average	1375(272)	2762(127)	0.50(0.08)

Supplementary Table 29: Replication study of the best hit of the second DoE.

	TON_{DMM}	TON_{MF}	Ratio
Experiment 1	2803	2230	1.26
Experiment 2	2410	2427	0.99
Experiment 3	2478	2547	0.97
Average	2563(210)	2401(160)	1.07(0.16)

Supplementary Table 30: Catalysis results of the fourth triple of the second DoE.

	TON_{DMM}	TON_{MF}	Ratio
Experiment 1	2450	2450	1.00
Experiment 2	2301	2356	0.98
Experiment 3	3079	2263	1.36
Average	2610(413)	2356(93)	1.11(0.22)

7 Appendix

7.1 NMR Spectra of the Ligand Precursor

7.1.1 Bis(hydroxymethyl)diphenylphosphonium chloride SI-2

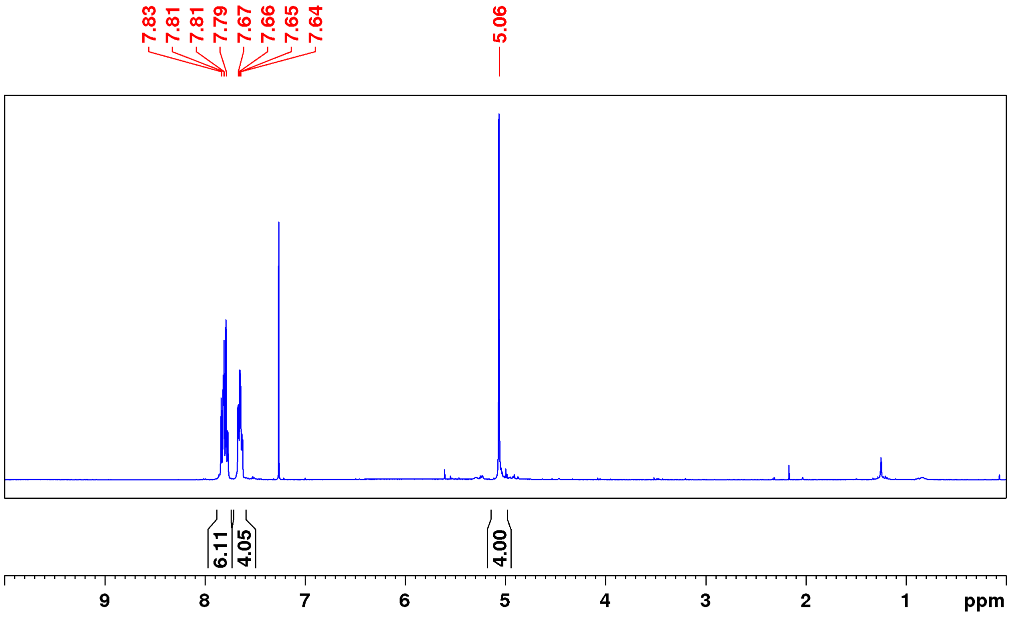

Supplementary Figure 70: ^{1}H NMR ($CDCl_3$, 400 MHz, 298 K) spectrum of compound **SI-2**.

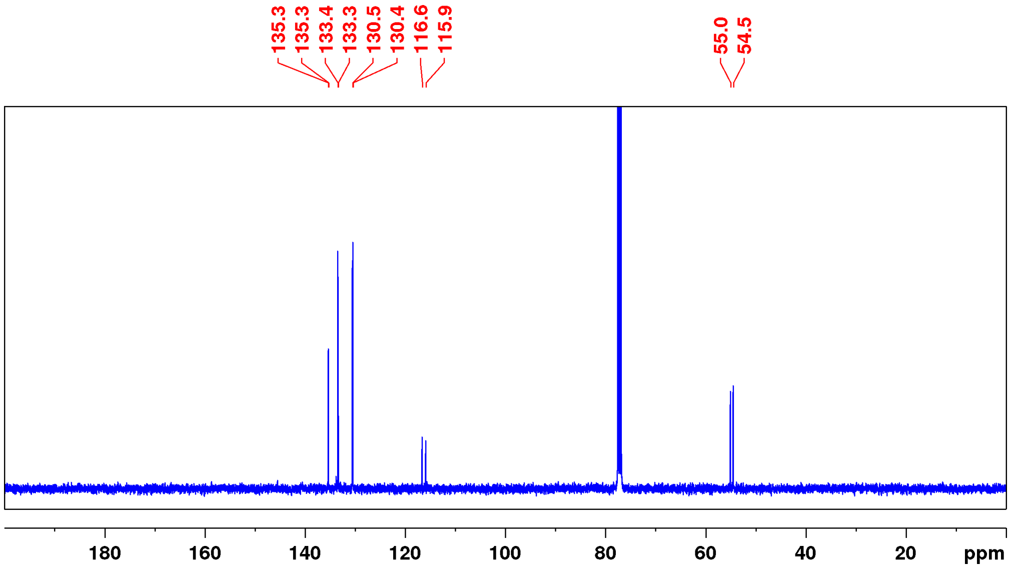

Supplementary Figure 71: $^{13}C\{^1H\}$ NMR ($CDCl_3$, 101 MHz, 298 K) spectrum of compound **SI-2**.

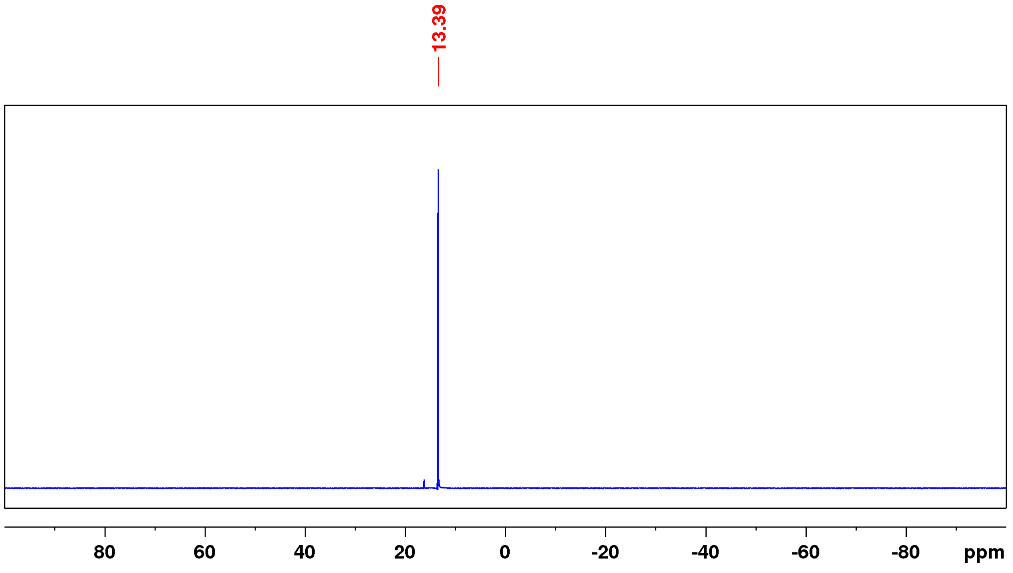

Supplementary Figure 72: $^{31}P\{^1H\}$ NMR ($CDCl_3$, 162 MHz, 298 K) spectrum of compound **SI-2**.

7.2 NMR Spectra of the Ligand

7.2.1 *N*-triphos^{Ph} SI-3

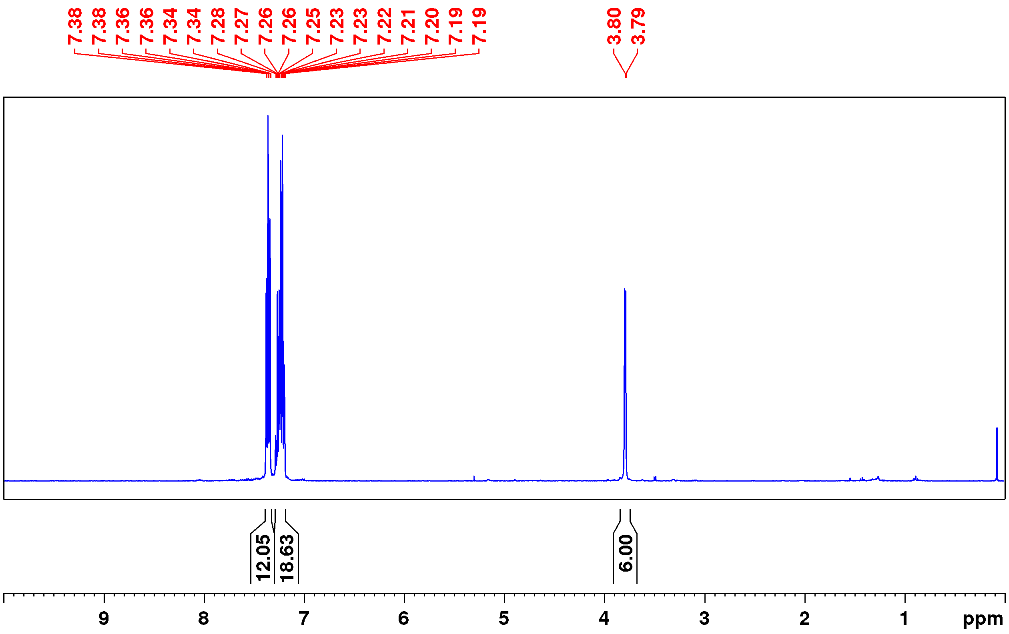

Supplementary Figure 73: ^{1}H NMR ($CDCl_3$, 400 MHz, 298 K) spectrum of compound **SI-3**.

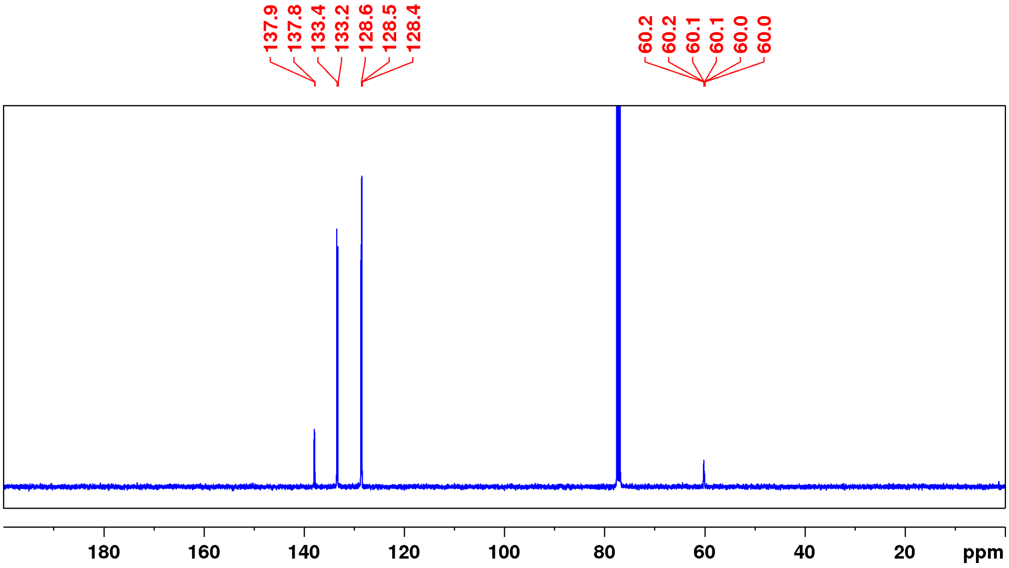

Supplementary Figure 74: $^{13}C\{^{1}H\}$ NMR ($CDCl_3$, 101 MHz, 298 K) spectrum of compound **SI-3**.

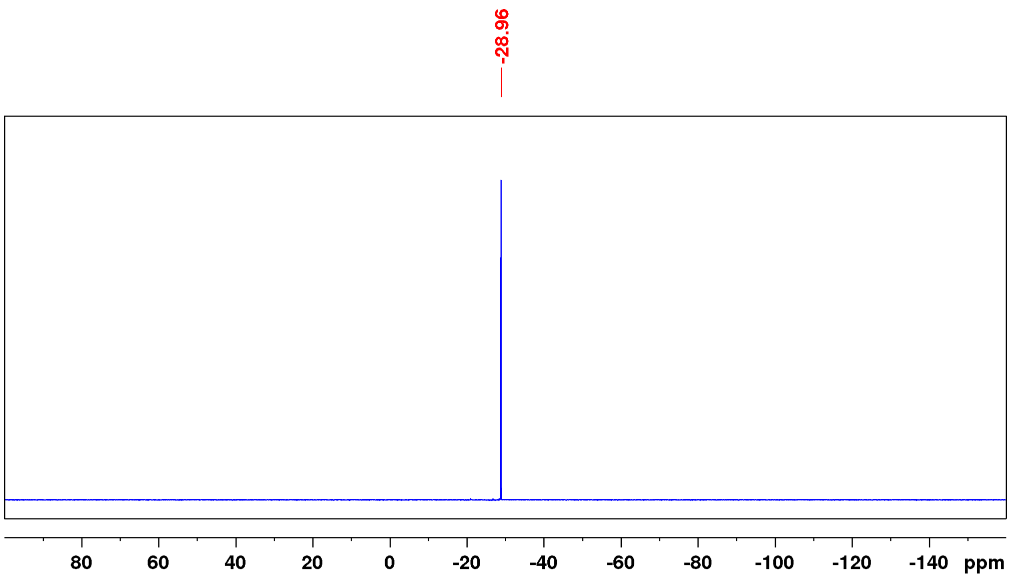

Supplementary Figure 75: $^{31}P\{^{1}H\}$ NMR ($CDCl_3$, 162 MHz, 298 K) spectrum of compound **SI-3**.

7.3 NMR Spectra of the Complex

7.3.1 [Ru(*N*-triphos^{Ph})(tmm)] SI-4

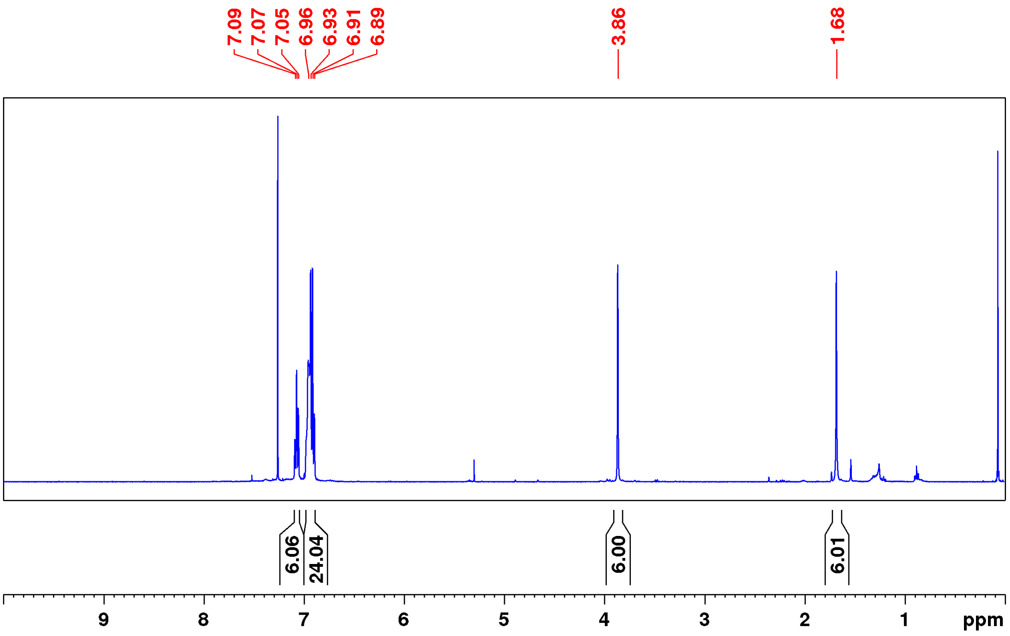

Supplementary Figure 76: ^{1}H NMR ($CDCl_3$, 400 MHz, 298 K) spectrum of compound **SI-4**.

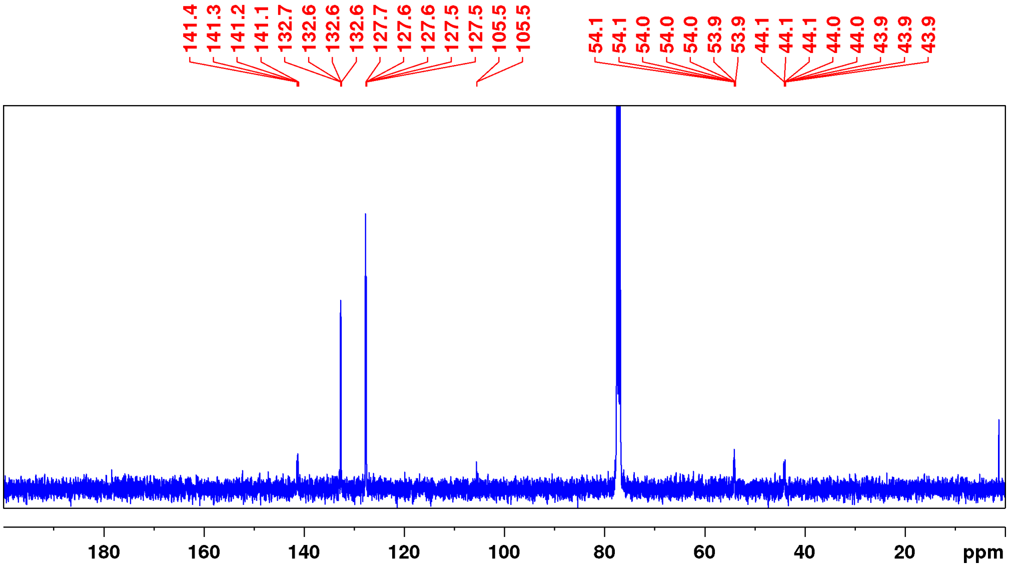

Supplementary Figure 77: $^{13}C\{^1H\}$ NMR ($CDCl_3$, 101 MHz, 298 K) spectrum of compound **SI-4**.

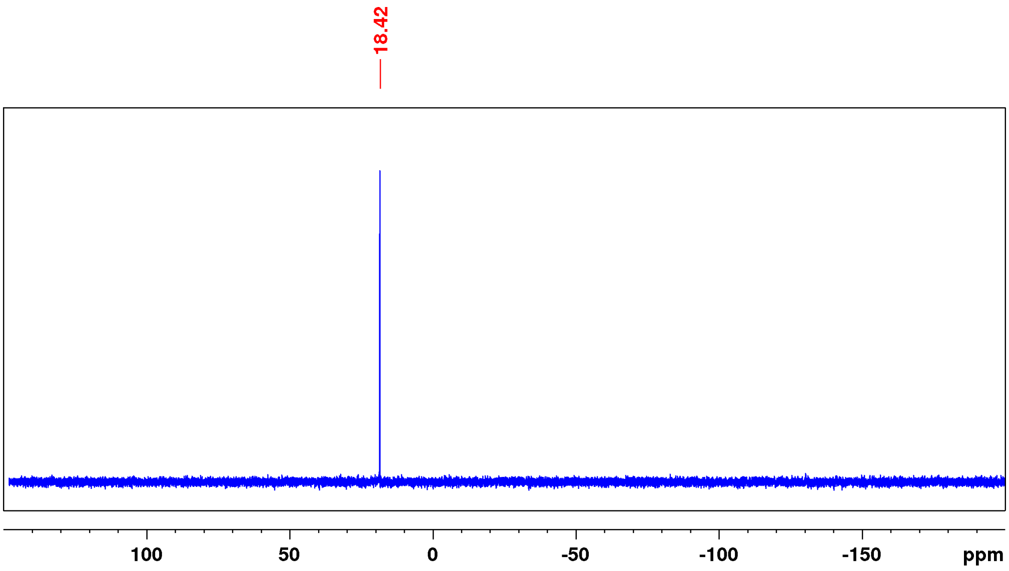

Supplementary Figure 78: $^{31}P\{^{1}H\}$ NMR ($CDCl_3$, 162 MHz, 298 K) spectrum of compound **SI-4**.

List of Abbreviations

Abbreviation	Full Name
add	Additive
ACN	Acetonitrile
ATR	Attenuated Total Reflection
b	Broad
BIC	Bayesian Information Criterion
cat	Catalyst
COSY	Correlation Spectroscopy
d	Doublet
DCM	Dichloromethane
DMM	Dimethoxymethane
DoE	Design of Experiments
EA	Elemental Analysis
ESI	Electrospray Ionization
Et	Ethyl
eq.	Equivalents
FIA	Flow Injection Analysis
FT	Fourier Transformation
FT-ICR	Fourier Transform Ion Cyclotron Resonance
HMBC	Heteronuclear Multiple-Bond Correlation
HR	High Resolution
HSQC-ME	Heteronuclear Single-Quantum Correlation Multiplicity-edited
IR	Infrared
IUPAC	International Union of Pure and Applied Chemistry
LTQ	Linear Ion Trap Quadrupole

Abbreviation	Full Name
m	Multiplet
m/z	Mass-to-charge ratio
M	Molecular ion
Me	Methyl
mes	Mesitylene
MF	Methyl Formate
MS	Mass Spectrometry
n	*normal*
NMR	Nuclear Magnetic Resonance
OLS	Ordinary Least Squares
Ph	Phenyl
ppm	Part Per Million
rt	Room temperature
s	Singlet
SI	Supporting Information
SPS	Solvent Purification System
THF	Tetrahydrofuran
tmm	Trimethylenemethane dianion
TON	Turnover Number
triphos	1,1,1-Tris(diphenylphosphinomethyl)ethane

Bibliography

1. G. R. Fulmer, A. J. M. Miller, N. H. Sherden, H. E. Gottlieb, A. Nudelman, B. M. Stoltz, J. E. Bercaw and K. I. Goldberg, *Organometallics*, 2010, **29**, 2176–2179.
2. J. Fawcett, P. A. T. Hoye, R. D. W. Kemmitt, D. J. Law and D. R. Russell, *J. Chem. Soc., Dalton Trans.* 1993, 2563–2568.
3. A. Phanopoulos, N. J. Brown, A. J. P. White, N. J. Long and P. W. Miller, *Inorg. Chem.* 2014, **53**, 3742–3752.
4. G. Märkl and G. Y. Jin, *Tetrahedron Lett.* 1981, **22**, 1105–1108.
5. J. Lloret Fillol, A. Kruckenberg, P. Scherl, H. Wadepohl and L. H. Gade, *Chem. Eur. J.* 2011, **17**, 14047–14062.
6. R Core Team, *R: A Language and Environment for Statistical Computing*, https://www.R-project.org, 2017.
7. A. Liaw and M. Wiener, *R News*, 2002, **2**, 18–22.
8. L. Breiman, A. Cutler, A. Liaw and M. Wiener, *randomForest: Breiman and Cutler's Random Forests for Classification and Regression*, https://CRAN.R-project.org/package=randomForest, 2018.
9. B. Wheeler, *AlgDesign: Algorithmic Experimental Design*, https://CRAN.R-project.org/package=AlgDesign, 2014.
10. A. Ghalanos and S. Theussl, *Rsolnp: General Non-linear Optimization Using Augmented Lagrange Multiplier Method*, https://CRAN.R-project.org/package=Rsolnp, 2015.
11. D. Sarkar, *Lattice*, Springer-Verlag, New York, 2008.
12. D. Sarkar, *lattice: Trellis Graphics for R*, https://CRAN.R-project.org/package=lattice, 2018.

13. K. Wright, *pals: Color Palettes, Colormaps, and Tools to Evaluate Them*, https://CRAN.R-project.org/package=pals, 2018.
14. S. Garnier, *viridis: Default Color Maps from 'matplotlib'*, https://CRAN.R-project.org/package=viridis, 2018.
15. H. Wickham, J. Bryan, RStudio, M. Kalicinski, K. Valery, C. Leitienne, B. Colbert, D. Hoerl and E. Miller, *readxl: Read Excel Files*, https://CRAN.R-project.org/package=readxl, 2019.
16. B. Ripley, B. Venables, D. M. Bates, K. Hornik, A. Gebhardt and D. Firth, *MASS: Support Functions and Datasets for Venables and Ripley's MASS*, https://CRAN.R-project.org/package=MASS, 2018.
17. M. Siebert, M. Seibicke, A. F. Siegle, S. Kräh and O. Trapp, *J. Am. Chem. Soc.* 2019, **141**, 334–341.
