# Supplementary material for: Identifying high-performance catalytic conditions for carbon dioxide reduction to dimethoxymethane by multivariate modelling
Source: Chem Sci. 2019 Oct 24;10(45):10466–74. doi: 10.1039/c9sc04591k (PMC7012071; doi:10.1039/c9sc04591k)

Electronic Supplementary Material (ESI) for Chemical Science.
This journal is © The Royal Society of Chemistry 2019

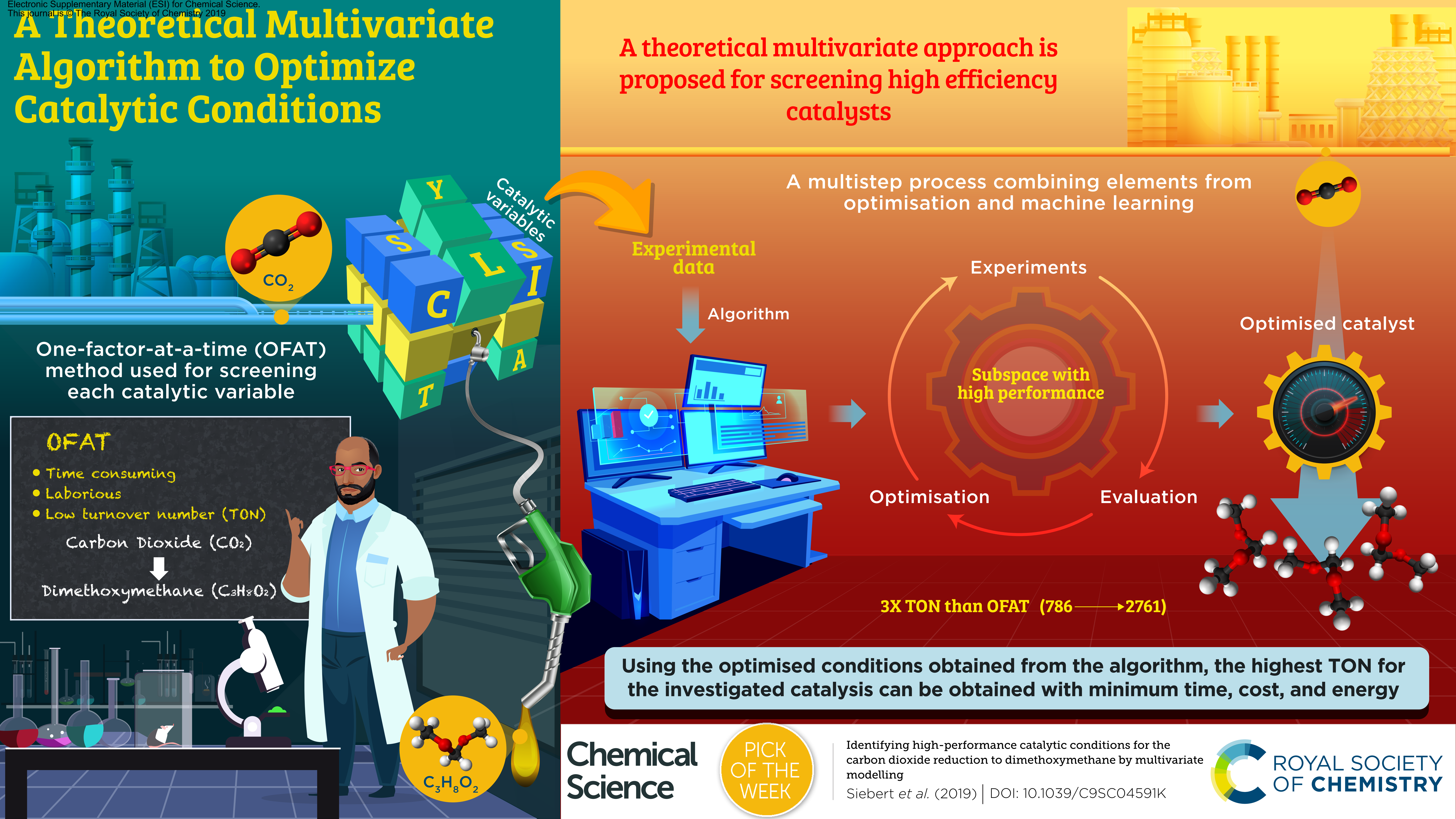

Supplement: Supplementary file 4 [file SC-010-C9SC04591K-s004.pdf]
